# Abortion stigma among people seeking abortion care in high-income countries: a mixed-methods systematic review

**DOI:** 10.1080/26410397.2026.2709964

**Published:** 2026-07-27

**Authors:** Jana Niemann, Marie Bernard, Dennis Jepsen, Laura Weinhold, Céline Miani, Nadja Freymüller, Claudia Luck-Sikorski

**Affiliations:** a Research Associate, Institute of Medical Sociology (IMS), Medical Faculty, Martin Luther University Halle-Wittenberg, Germany.; d Research Assistant, Institute of Medical Sociology (IMS), Medical Faculty, Martin Luther University Halle-Wittenberg, Germany; e Professor for Gender and Social Epidemiology, Department of Epidemiology and International Public Health, School of Public Health, Bielefeld University, Bielefeld, Germany; f Research Associate, Institute for Rehabilitation Medicine, Medical Faculty, Martin Luther University Halle-Wittenberg, Germany; Research Associate, Department of Sustainable Environmental Health Sciences, Medical School OWL, Bielefeld, University, Bielefeld, Germany; g Professor of Mental Health and Psychotherapy, SRH University of Applied Health Sciences, Gera, Germany

**Keywords:** abortion, pregnancy termination, social stigma, social discrimination, abortion seekers, reproductive health

## Abstract

Abortion is a common reproductive healthcare process that is often stigmatised. Research on abortion stigma has grown significantly since the last major review over a decade ago, and there is a pressing need for an updated, comprehensive systematic review. The purpose of this review is to examine the extent and subjective experiences of abortion stigma among those seeking an abortion in high-income countries. We aim to explore the theoretical conceptualisations of abortion stigma in relevant studies. We conducted a mixed-method systematic review following the JBI and PRISMA guidelines. PubMed, CINHAL, PsychINFO, LIVIVO and the Cochrane Library were searched for peer-reviewed articles. Quantitative studies were summarised narratively. Qualitative studies were synthesised using the JBI meta-aggregative approach. We included 41 qualitative, nine quantitative and three mixed-methods studies. Most studies lacked a substantial theoretical conceptualisation of abortion stigma. Quantitative studies reported prevalence rates of perceived abortion stigma ranging from 37% to 60%, suggesting that stigma remains a common experience among abortion seekers. Findings also indicate associations between abortion stigma and various sociodemographic factors (e.g. religion, race and age), as well as adverse mental health outcomes. In the qualitative studies, people seeking abortion care reported experiencing and anticipating judgment from healthcare professionals, anti-abortion activists and their close social circle. Their experiences also centred on the internalisation of shame and guilt. Some studies highlighted the mitigating effect of social support. Longitudinal and mixed-methods approaches with consistent assessment would be useful to better understand the developmental pathways of abortion stigma. This understanding is necessary to provide individual and structural support for people seeking abortion care. *DOI: 10.1080/26410397.2026.2709964 Trial registration number*: https://doi.org/10.17605/OSF.IO/2XPJD.

## Plain language summary

Abortion is a common healthcare procedure, but it often faces stigma. Despite many recent studies on this topic, there hasn't been an updated overview since 2016. We reviewed 53 research articles to understand how and to what extent abortion stigma is experienced in high-income countries. Quantitative studies mainly looked at how common abortion stigma can be and how it relates to mental health and decision-making. Qualitative studies provided a more profound insight. They showed that people seeking abortions often feel judged by healthcare workers, anti-abortion activists and their friends and family. Qualitative studies underline how people who seek an abortion feel ashamed and guilty. However, having support from loved ones could help lessen these feelings. In conclusion, we found that abortion is a stigmatised process. Future research should explore the support systems available to people seeking abortions. Understanding these supports can help improve structural-level care and ensure better assistance for people navigating abortion access.

## Résumé

L’avortement est une pratique courante de santé reproductive qui est souvent stigmatisée. Les recherches sur la stigmatisation de l’avortement se sont multipliées depuis la dernière revue d’envergure, il y a plus de dix ans, et il est urgent de disposer d’un examen systématique complet et actualisé. Cet examen vise à explorer l’ampleur et les expériences subjectives de la stigmatisation de l’avortement chez les personnes souhaitant interrompre leur grossesse dans les pays à revenu élevé. Nous cherchons à étudier les conceptualisations théoriques de cette stigmatisation dans les études pertinentes. Nous avons mené un examen systématique à méthodes mixtes, conformément aux lignes directrices de JBI et PRISMA. Les bases de données PubMed, CINHAL, PsychINFO, LIVIVO et Cochrane Library ont été consultées afin d’identifier les articles évalués par les pairs. Les études quantitatives ont fait l’objet d’un résumé narratif. Les études qualitatives ont été synthétisées à l’aide de l’approche méta-agrégative de JBI. Nous avons inclus 41 études qualitatives, neuf études quantitatives et trois études à méthodes mixtes. La plupart des études ne présentaient pas une conceptualisation théorique approfondie de la stigmatisation de l’avortement. Les études quantitatives ont rapporté des taux de prévalence de la stigmatisation perçue de l’avortement allant de 37% à 60%, ce qui suggère que la stigmatisation reste une expérience courante chez les personnes souhaitant avorter. Les résultats indiquent aussi des associations entre la stigmatisation de l’avortement et plusieurs facteurs sociodémographiques (par exemple la religion, la race, l’âge) ainsi que des conséquences néfastes sur la santé mentale. Dans les études qualitatives, les personnes souhaitant avorter ont signalé qu’elles avaient rencontré et anticipé des jugements de la part des professionnels de santé, des militants anti-avortement et de leur entourage immédiat. Leurs expériences se concentraient également sur l’intériorisation de la honte et de la culpabilité. Certaines études ont mis en lumière l’effet bénéfique du soutien social. Des méthodes mixtes et longitudinales, avec une évaluation cohérente, seraient utiles pour mieux comprendre l’évolution de la stigmatisation de l’avortement. Cette compréhension est nécessaire pour prodiguer un appui structurel et individuel aux personnes souhaitant avorter.

## Resumen

El aborto es un proceso común de los servicios de salud reproductiva que a menudo es estigmatizado. Las investigaciones sobre el estigma del aborto han aumentado considerablemente desde la última revisión importante realizada hace más de una década, y existe la necesidad apremiante de una revisión sistemática, integral y actualizada. El propósito de esta revisión es examinar el grado y las experiencias subjetivas de estigma del aborto entre personas que buscan un aborto en países de altos ingresos. Pretendemos explorar las conceptualizaciones teóricas del estigma del aborto en estudios pertinentes. Realizamos una revisión sistemática de métodos mixtos siguiendo las directrices de JBI y PRISMA. Buscamos artículos revisados por pares en PubMed, CINHAL, PsychINFO, LIVIVO y *Cochrane Library*. Resumimos los estudios cuantitativos de manera narrativa. Sintetizamos los estudios cualitativos utilizando el enfoque de meta-agregación de JBI. Incluimos 41 estudios cualitativos, nueve cuantitativos y tres de métodos mixtos. La mayoría de los estudios carecían de una conceptualización teórica sustancial del estigma del aborto. Los estudios cuantitativos informaron tasas de prevalencia de estigma de aborto percibido de entre 37% y 60%, lo que indica que el estigma continúa siendo una experiencia común entre las personas que buscan un aborto. Además, los hallazgos indican asociaciones entre el estigma del aborto y diversos factores sociodemográficos (p. ej., religión, raza, edad), así como resultados adversos de salud mental. En los estudios cualitativos, las personas que buscan servicios de aborto informaron experimentar y anticipar ser juzgadas por profesionales de salud, activistas antiaborto y personas cercanas en su círculo social. Sus experiencias se centraban en la internalización de vergüenza y culpa. Algunos estudios destacaron el efecto mitigador del apoyo social. Los enfoques longitudinales y de métodos mixtos con una evaluación sistemática serían útiles para entender mejor las vías de desarrollo del estigma del aborto. Esta compresión es necesaria para brindar apoyo individual y estructural a las personas que buscan servicios de aborto.

## Introduction

Access to abortion is a fundamental human right and an essential component of sexual and reproductive health.^1–4^ It includes not only the provision of safe abortions but also access to information about the procedure and post-abortion care to ensure people's physical and mental well-being.^1,5^ However, the ability to exercise this right has historically been and continues to be shaped and constrained by shifting political, social and cultural forces.^4^ The liberalisation of abortion laws is often the result of long-standing advocacy and legal reform, but even once established, such rights remain highly contested and vulnerable to reversal. Abortion-related stigma and discrimination can challenge sexual and reproductive rights, as they are social constructs rooted in efforts to control the sexuality and related behaviours of women and gender minorities, enforcing norms considered acceptable by the majority.^6^ The American Psychological Association has identified stigma as a major risk factor for the mental health of women who have had abortions, which can lead to numerous consequences, including anxiety, increased physiological distress, social withdrawal and avoidance, and depression.^7^

In line with our study protocol,^8^ we understand abortion stigma as a social process that is always context-dependent and can be viewed as a form of exercised power^9,10^ manifesting at the micro (individual), meso (societal) and macro (structural) levels.^11^ Our theoretical conceptualisation of abortion stigma among people seeking abortion care draws on the work of Rai et al.^12^ and Strong.^11^ Rai et al.^12^ emphasise that stigma is not merely an individual attribute but rather emerges through social processes shaped by power dynamics. Strong's^11^ analysis illustrates how individuals experience support or lack thereof in abortion-related care across various social levels. Our theoretical framework conceptualises abortion stigma as a multilevel phenomenon that operates across intra- and interpersonal, institutional and structural levels. These are shaped by and mutually reinforce one another through social interactions, health care practices, laws and broader cultural norms.^11^

Abortion stigma can be understood from two complementary perspectives: the perspective of those who stigmatise and the perspective of those who are stigmatised. ***Enacted stigma*** refers to the stigmatising attitudes, behaviours and practices directed towards people who are associated with abortion.^10,13^ From the perspective of people seeking abortion care, stigma may manifest in different forms, including experienced, perceived, internalised and anticipated stigma. ***Experienced stigma*** refers to direct encounters with devaluation, discrimination or hostility, such as belittlement by healthcare providers, condemnation by people in one’s social environment or hostility from protesters. ***Perceived stigma*** refers to the awareness or belief that abortion is morally condemned or socially disapproved of within one’s community or society.^14^ This perception may exist regardless of whether an individual has actually *experienced stigmatisation*. ***Internalised stigma*** occurs when individuals adopt negative societal beliefs about abortion and apply these beliefs to themselves, resulting in shame, guilt or self-blame.^11,15^ This may reduce their perceived entitlement to health care services. ***Anticipated stigma*** refers to the expectation of being judged, mistreated or discriminated against. This expectation may deter individuals from engaging with formal healthcare systems or disclosing their abortion experience to people in their social environment, thereby limiting access to professional care and social support.^16^

Experienced, perceived, internalised and anticipated stigma interact with each other and can result in individuals delaying or avoiding abortion care. They may travel long distances to maintain anonymity or withhold information from providers, compromising timely and safe access to care.^15^ Abortion stigma can also lead to silence during medical consultations, concealment of abortion history, avoidance of post-abortion care or follow-up services and resorting to unsafe or informal abortion methods due to mistrust of the health system.^17^ Together, these interrelated forms of abortion stigma create significant barriers to timely, comprehensive and non-discriminatory abortion care.

The most recent systematic review of existing research on abortion stigma among abortion seekers was conducted by Hanschmidt et al. in 2016. The authors included three quantitative and four qualitative studies that examined stigma among individuals who had sought abortions.^18^ In their review, the authors examined the sources of abortion-related stigma and analysed how and to what extent abortion seekers perceive, internalise and experience abortion stigma. They also explored the potential moderators of abortion stigma, strategies used to manage it and the consequences experienced by people seeking abortion. They found that abortion seekers perceive abortion-related stigma from society, the community and significant others, as well as abortion and general healthcare providers.^18^ Many women also anticipated negative judgment from society, their communities and close social circles, including family and friends. This often led to secrecy surrounding their abortion, which was associated with psychological distress and emotional isolation. Internalised stigma was also prevalent, as women expressed feelings of shame, guilt and self-blame, particularly those with strong religious affiliations or limited social support. However, the authors also found that some people who obtained abortion services refused to accept these negative feelings. Although experienced stigma was less frequently documented, some women reported subtle forms of discrimination, such as judgmental attitudes from healthcare providers or social exclusion by peers.

Hanschmidt et al. acknowledged the important limitations of their review. Notably, not all quantitative instruments used were validated, and in some cases, they assessed other components of stigma, such as anticipated stigma, or diverged from established definitions, particularly in the case of internalised stigma. Since Hanschmidt et al.^18^ published their review of abortion stigma, the body of literature on this topic has increased substantially. Therefore, an updated and critical review of the current state of knowledge is required. Moreover, we aimed to apply a more refined conceptualisation of stigma, specifically distinguishing between experienced, perceived, internalised and anticipated stigma. In addition, significant societal shifts, evolving public discourse, and changing legal and policy landscapes around abortion, for example, more permissive legislation in Ireland (2018)^19^ and Australia (2019)^20^ or more restrictive legalisation in the USA (2022)^21^ and Poland (2020),^22^ may have influenced both the experience and representation of abortion stigma in recent studies. These contextual developments underscore the need to revisit and reassess the current evidence-based research.

We believe that the following factors are important when establishing the context of our review. Access to sexual and reproductive healthcare, including abortion services, differs tremendously between high-income countries (HICs) and low- and middle-income countries.^2,23^

Access to legal abortion without restrictions is significantly higher in high-income countries (HICs) than in low- and middle-income countries (LMICs), at 81% versus 29%.^23^ Women in HICs generally experience more consistent access to a broad range of sexual and reproductive health services, including abortion.^2^ Although many LMICs have safe and accessible legal abortion services, the overall variability in legal frameworks and social context can complicate direct comparisons of abortion stigma across these settings. To ensure a more contextually coherent analysis, we focus exclusively on HICs, which share more comparable health system capacities, legal environments, and SRH service access. This focus allows for a clearer exploration of how stigma manifests across diverse high-income settings, minimising confounding factors related to service availability and safety that may differ substantially between LMICs.^24^ However, it is important to acknowledge that HICs are not homogeneous. Differences in abortion legislation, healthcare system organisation, public discourse and religious or cultural norms can lead to substantial variations in how abortion stigma is experienced. For example, the availability of abortion services varies among countries.^4^ Therefore, we aimed to capture this heterogeneity in our mixed-methods systematic review (MMSR), which investigated the extent and subjective experiences of abortion stigma among people seeking abortion care in HICs.

Against this backdrop, our MMSR seeks to provide a comprehensive understanding of the extent and nature of abortion stigma in HICs. To guide our investigation, we formulated the following research questions:

**RQ1**: What definition(s) did the authors employ for their theoretical conceptualisation of abortion stigma?

**RQ2**: To what extent do people seeking abortion care experience abortion stigma in HICs from a quantitative perspective?

**RQ3**: What are the experiences concerning abortion stigma among people seeking abortion care in HICs from a qualitative perspective?

Therefore, we aimed to summarise the quantitative and qualitative findings and provide updated evidence for research, policy and practice.

## Methods

This systematic review followed the PRISMA guidelines^25^ and JBI guidelines^†^ for mixed-methods systematic reviews.^26^ The protocol^8^ for this review has been previously published.

### Inclusion criteria

*Participants:* This systematic review included studies on abortion-related stigma among abortion seekers. We excluded studies that specifically examined abortion-related stigma among minors who needed to overcome more barriers, such as consent from a legal guardian to obtain abortion services.^27^

*Phenomena of interest:* We considered studies that examined the extent of abortion stigma (prevalence or covariate) and/or explored the subjective experiences of people seeking abortion care.

*Context:* We only included studies from HICs.^24^

*Types of studies:* We included peer-reviewed publications with quantitative, qualitative and mixed-method research designs. The quantitative research design included both clinical and non-clinical studies. Qualitative research designs included but were not limited to in-depth interviews, group discussions, (social) media analysis and autobiographic work.

### Search strategy

We conducted a systematic literature search between January and February 2023 (updated 27 February 2024) for peer-reviewed qualitative, quantitative and mixed-methods studies reporting abortion stigma in HICs. We searched relevant databases, including MEDLINE (EBSCOhost), CINHAL (EBSCOhost), PsychINFO (EBSCOhost), LIVIVO and Cochrane Library for appropriate studies. Grey literature was not included in the scope of this review. The search was independently conducted by two reviewers (JN and MB) using the following keywords adapted from a previous systematic review:^18^ [abortion OR termination of pregnancy OR voluntary interruption of pregnancy] AND [stigma* OR discrimination*]. The search was restricted to the titles and abstracts. This systematic review aimed to update the existing work by Hanschmidt et al.;^18^ thus, we only included studies published from March 2015 onwards.

### Study selection

After completing the search, all records were imported into Rayyan, a web-based application designed to support the screening and selection of studies for systematic reviews. Duplicate entries were identified and removed within the platform.^28^ Two researchers (JN and MB) independently screened the titles and abstracts for the assessment of our inclusion criteria. Citations of eligible studies were transferred to Excel for full-text screening. They were independently screened by two researchers (JN and MB). The reasons for the exclusion of full texts were recorded and reported. Disagreements during the study selection process were resolved through discussions. The stages and results of the search and study selection processes are shown in Figure 1.

**Figure 1.**
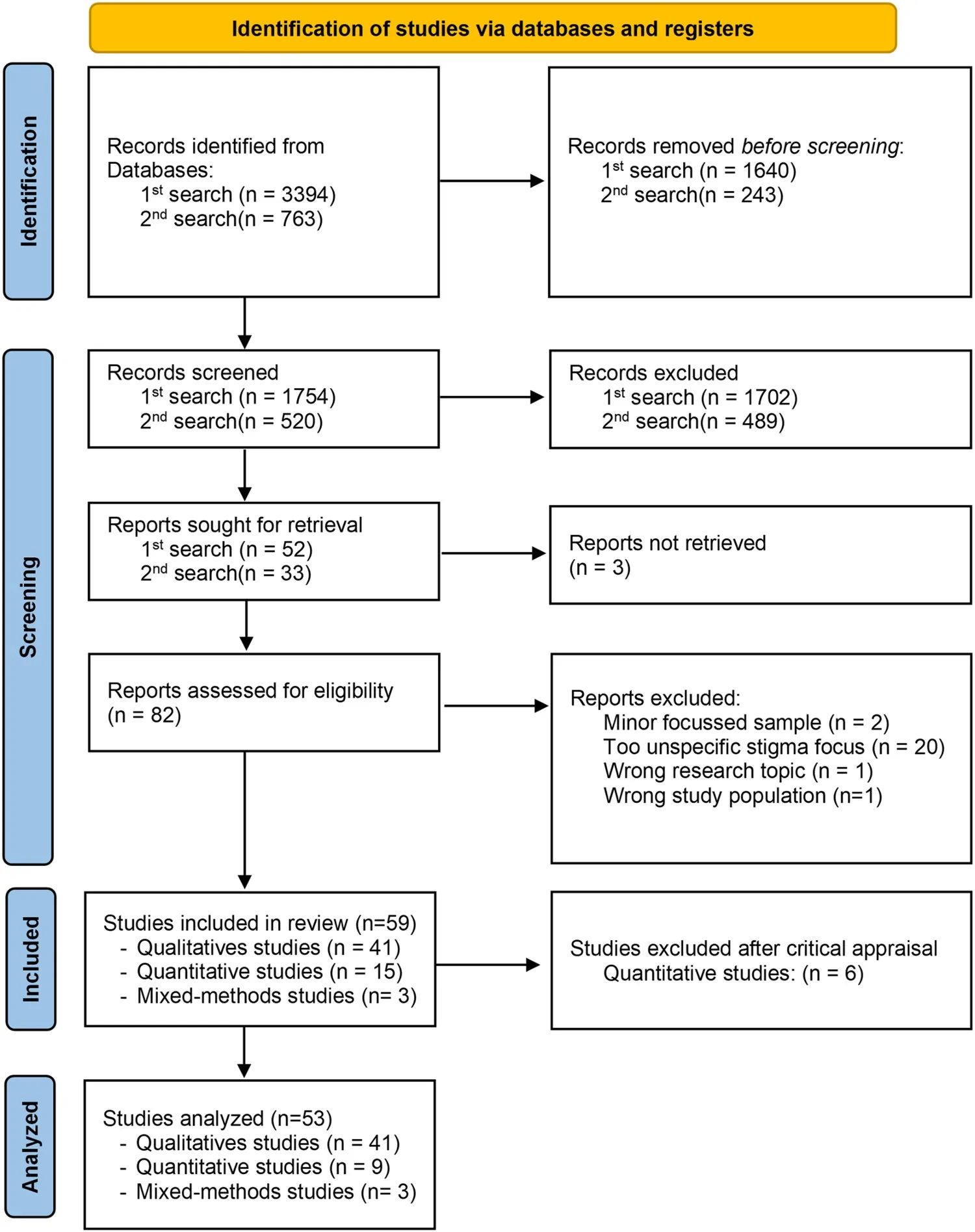
PRISMA flow diagram of the selection process

This figure shows our selection process for the included studies. The first search was conducted from January to February 2023. This search was updated on 27 February 2024 (second search).

### Critical appraisal

#### Quantitative studies

We used the ROBINS-E tool (Risk Of Bias In Non-randomised Studies of Exposures) to assess the risk of bias in the included quantitative studies.^29,30^ Two researchers (DJ and JN) critically appraised quantitative studies for methodological quality using the ROBINS-E tool.^29^ Any disagreements that arose during this process were resolved by discussion.

#### Qualitative studies

One researcher (JN) critically appraised qualitative studies for methodological quality using the JBI-QARI^‡^ checklist for interpretive and critical research.^31^ To ensure rigour, 20% of the included studies were assessed by a second researcher (LW) (Krippen-Dorff’s alpha reliability coefficient^32^ = 0.945). Inconsistencies were discussed until consensus was achieved.

### Data extraction and synthesis

Following the JBI guidelines for MMSR, we chose a convergent separated approach to synthesise quantitative and qualitative data from the studies. We analysed the quantitative and qualitative data separately and integrated them. Data were extracted by coding in MAXQDA 2022 for both strands of research.

#### Quantitative studies

The data were extracted by three researchers (DJ, JN and MB) with the following categories: definition of (abortion) stigma described in the article, type of abortion stigma as outcome and/or predictor, geographical location, sample size, validity, reliability, prevalence of abortion stigma and explored associations with independent variables.

Due to the diversity in the measurement of abortion stigma in quantitative studies, we decided against a meta-analysis. We opted for a narrative synthesis,^26^ which was performed by DJ.

#### Qualitative studies

We used the JBI meta-aggregation approach for qualitative studies^31^ (see Figure 2). This involved extracting findings (cf. Supplementary Table 1) from the included studies, developing categories for these findings and synthesising them into comprehensive statements (cf. Supplementary Table 2). One author (JN) extracted the data using the standardised JBI data extraction tool,^33^ which was adapted according to the phenomenon of interest of this study. Subsequently, the findings were synthesised and supported by illustrations. Following the ConQual approach, two researchers (JN and LW) assigned a level of credibility as unequivocal, credible or not. Our coding process was structured through our theoretical conceptualisation of abortion stigma among people seeking abortion care. Hence, we formed four deductive categories: 1. Experienced stigma, 2. Perceived stigma, 3. Internalised stigma and 4. Anticipated stigma. Inductive subcategories were generated during the coding process.

**Figure 2.**
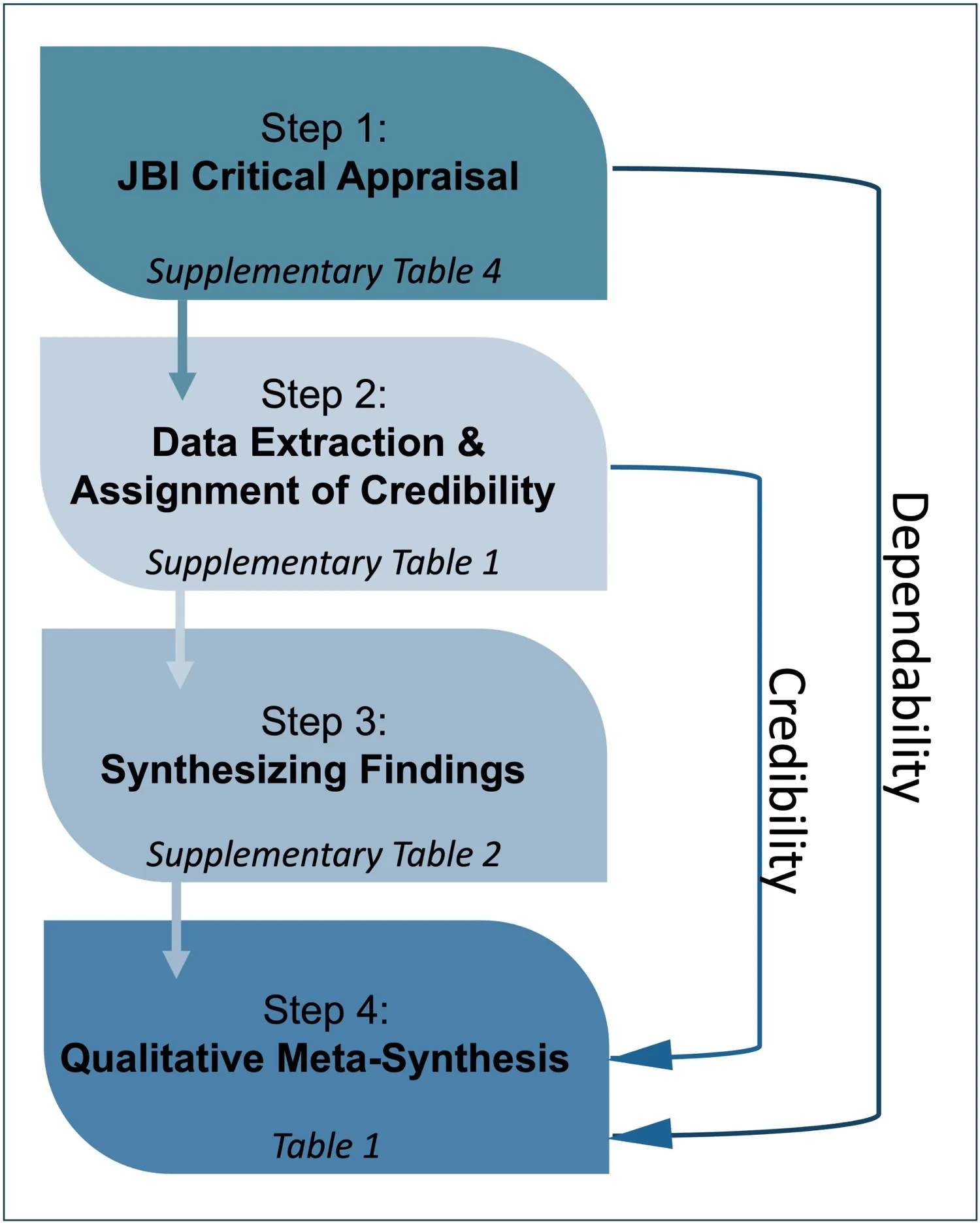
JBI meta-aggregation approach for qualitative studies overview

We used the ConQual approach^§^ to assess confidence in the qualitative research synthesis findings. Dependability was determined by analysing the JBI critical appraisal tool, while credibility was assessed based on the quality and details of the findings. Four researchers (JN, LW, DJ and MB) confirmed the appraisal, extraction and synthesis of these findings.

#### Integration of quantitative and qualitative evidence

After extracting and synthesising the separate strands of quantitative and qualitative research, we utilised configurative analysis to integrate the evidence. This analysis involved juxtaposing the synthesised evidence from both sources to determine whether the findings complemented each other. To achieve this, we repeatedly compared the results of the quantitative synthesis with the findings derived from the qualitative synthesis. We followed the guidelines provided by the JBI MMR Methods.^26^ Two researchers (DJ and JN) performed the integration. The integration is summarised in an illustration based on the considerations of Rai et al.^12^ and Strong.^11^

### Author positionality

The author team comprised researchers from diverse disciplinary backgrounds, including sociology, public health, psychology, social epidemiology and nutritional sciences. All team members identified as cisgender and white. We acknowledge that our personal and disciplinary backgrounds may have influenced various aspects of this study, from its conceptualisation to the writing process.

As a team, we approach abortion as a fundamental sexual and reproductive health right essential for upholding human dignity. We view abortion-related stigma as a major barrier to realising this right and achieving universal access to health care. In conducting this review, we engaged not only with the voices and experiences of study participants but also with the interpretations and narratives offered by the original study authors, both included and excluded from our final analysis. A more detailed account of personal and disciplinary reflexivity is provided in Supplementary Table 3.

## Results

Of the 4,157 records identified, 59 were critically appraised and summarised in our MMSR (41 qualitative, 15 quantitative and 3 mixed-methods studies).

### Critical appraisal

#### Quantitative studies

Out of 15 quantitative studies considered, six^34–39^ were excluded based on the preliminary considerations of the Robins-E tool, due to inappropriate outcome measurement. Among the remaining nine studies, four^40–43^ were at high risk of bias, one^44^ raised concerns and four^45–48^ were deemed to have a low risk of bias. We also included three mixed-methods studies. However, given the insufficient focus on abortion stigma within their quantitative components, we excluded these from the analysis and considered only the qualitative data.

#### Qualitative studies

Overall, we scored most studies with a moderate level of dependability using the JBI critical appraisal tool.^31^ All but one study^49^ adequately represented participants’ voices concerning our research question (Q8). A more detailed description of our critical appraisal can be found in Supplementary Table 4.

### Main description

After critical appraisal, 53 studies were included in data extraction. Of these, 41 used qualitative, nine quantitative, and three mixed-methods approaches. Due to the lack of focus on abortion stigma in their quantitative components, we included only the qualitative research strands of the mixed-methods studies and, therefore, counted them as qualitative in the following sections. Forty-eight studies included women as participants, one study each included participants identified as “female and non-binary” or “cisgender female and gender fluid”^50,51^ and three studies included participants identified as “cisgender female, transgender, non-binary”.^50,52,53^ Supplementary Table 5 describes the characteristics of all the included studies. Among the quantitative studies, six were from the United States (US),^40–43,46,47^ two from Germany^45,48^ and one from Ireland.^44^ Of the qualitative studies, 20 were conducted in the USA,^51,54–72^ eight in the United Kingdom,^73–80^ five in Australia,^52,53,81–83^ three in Canada,^84–86^ six in other countries^50,87–91^ and one US-based website.^92^

### Definitions

Across the studies included in this review, the most frequently applied conceptual framework for abortion-related stigma was developed by Kumar, Hessini and Mitchell,^93^ with additional reference to the models proposed by Norris et al.^94^ and Cockrill and Nack.^15^

A total of 17 studies^40–42,44,47,55,59,61,65,70,73,76,77,81,83,88,90^ cited the definition provided by Kumar, Hessini and Mitchell,^93^ who describe abortion stigma as “a negative attribute ascribed to women who seek to terminate a pregnancy that marks them, internally or externally, as inferior to ideals of womanhood” (p. 628). This perspective builds upon Goffman’s foundational concept of stigma^95^ as a “deeply discrediting” attribute and integrates elements from Link and Phelan’s framework,^10^ emphasising labelling, stereotyping and social exclusion. Expanding on this work, Norris et al.^94^ identified contributing factors, such as fetal personification, legal constraints and discursive constructions of gender and morality, all of which influence stigma at the social, institutional and political levels. In this review, five studies^40,55,61,65,70^ referred to Norris et al.'s^94^ definition of abortion stigma. In contrast to these more structural perspectives, Cockrill and Nack^15^ focused on distinguishing various forms of stigma experienced by individuals who have undergone abortion. They conceptualised abortion stigma as a multidimensional phenomenon encompassing internalised stigma (self-judgment), felt stigma (anticipated judgment by others) and enacted stigma (actual experiences of discrimination or judgment). Nine studies^41,45,47,48,61,63,70,76,83^ included in this review referenced the approach by Cockrill and Nack.

Beyond these widely adopted frameworks, only a small number of studies in this review employed alternative conceptualisations. For example, Hanschmidt et al.^18^ developed a definition that integrates both the individual and structural aspects of stigma, with particular emphasis on the context of Germany. This definition was used by one study.^40^ Four studies^40,41,45,76]^ utilised Herek's^96^ definition of sexual stigma, one study^69^ exclusively used generic stigma definition(s) and 31 studies^43,46,50–54,56–58,60,62,64,66–68,71,74,75,78–80,82,84–87,89,91,91,92^ did not explicitly state any definition of stigma (cf. Supplementary Table 6). Two studies^44,83^ referred to other definitions in the background and explicitly stated a self-definition.

### Quantitative studies

Supplementary Table 7 presents the background and results of the included quantitative studies. All the included studies focused on abortion stigma among people seeking abortion care, either as independent or dependent variables. Seven studies used a cross-sectional design,^42,43,46–48^ one a longitudinal,^40^ and one was a randomised controlled study.^41^ Five^41,44–47^ of the included studies measured abortion stigma with the Individual Level Abortion Stigma (ILAS) scale, two^42,48^ with just two different subscales of the ILAS scale, and two^40,43^ studies used other single-item measures.

The ILAS is a 20-item measure that consists of four subscales: worries (seven items), isolation (six items), self-judgement (five items) and community condemnation (two items), with higher scores indicating a higher level of abortion-related stigma (range 0–4). Three studies reported total ILAS mean scores of 1.02^41^, 1.21^47^ and 1.35^46^, while other studies reported mean scores for specific ILAS subscales. Across studies, self-judgment was consistently among the highest-rated subscales, with mean scores ranging from 1.54^44^ to 2.00.^46^ Community condemnation also showed comparatively high scores, ranging from 0.54^45^ to 2.39^44^, with US-based studies reporting means of 1.40^42^, 1.47^47^ and 1.84.^46^ Worries about judgment were generally lower, ranging from 0.65^45,47^ to 1.74^44^, with Mehta et al^46^ reporting a mean score of 0.86. Stigma-related isolation ranged from 0.67^45^ to 1.27^44^, with additional studies reporting means of 0.81^47^ and 1.22.^46^ Kerns et al.,^42^ who reported only two ILAS subscales, found mean scores of 1.70 for self-judgment and 1.40 for community condemnation, respectively. Overall, the ILAS subscale findings suggest that internalised stigma (self-judgment subscale) was more pronounced than anticipated stigma (i.e. worries about judgment or isolation subscale).

Biggs et al.^40^ reported a 60% prevalence rate of perceived abortion stigma by the community and 56% by people close to the respondents. Furthermore, one study^43^ that used a single-item measure found a 37.4% prevalence of perceived abortion stigma.

#### Sociodemographic variables

One study indicated various sociodemographic factors related to higher values of felt abortion stigma, such as being married or Catholic/ Protestant religious affiliation.^40^ A single study showed that Black people were less likely to report felt abortion stigma than White people.^40^ Regarding the relationship between younger age and felt abortion stigma, one study found a significant positive association,^40^ whereas another did not.^45^ A study also found no significant associations between ILAS score and sociodemographic factors such as age, race, income and body mass index.^47^

#### Pregnancy status

An association was found between higher gestational age and higher scores on the ILAS subscale of self-judgment of abortion seeking.^45^ In pregnancies with a diagnosed fetal abnormality, perceived low survival chance was associated with lower values in both ILAS subscales of worry about judgment and self-judgment.^45^ In the same study, an association was found between the potential protective role of intimate partner support and lower ILAS scores.^45^ In another study focusing on people seeking abortion in the second trimester, no relationship was identified between ILAS score and parity, gestational age at the time of the abortion or reasons for ending the pregnancy, such as health problems of the fetus, relational problems and pressures, pregnancy as a result of rape/ incest, economic hardship or concerns about the pregnancy timing, spacing or child rearing.^47^

#### Mental health and somatic symptoms

Most studies investigated the link between felt abortion stigma and mental health. Previous experiences with depression and anxiety,^40^ depressive symptoms, low self-esteem and grief and trauma^48^ were identified as potential factors associated with increased felt abortion stigma. Furthermore, correlations were identified between ILAS score and positive screening for depression and anxiety among women in the second trimester.^47^ Higher levels of perceived abortion stigma^40^ and internalised abortion stigma^44,48^ have been linked to an increased risk of (long-term) psychological distress following abortion seeking. Again, intimate partner support was identified as a mitigating factor for mental stress.^48^ In another study, only self-judgment (ILAS subscale) was considered a significant risk factor of post-abortion grief.^42^ No corresponding relationship was found between other ILAS subscales and grief, post-traumatic stress or poorer mental health.^42^ Additionally, the study conducted by Steinberg et al.^43^ indicated that perceived abortion stigma might be a predictor of symptoms of depression, anxiety and psychological stress before an abortion.

The sole investigation into the potential link between felt abortion stigma and somatic symptoms was conducted by O’Donnell et al.^44^ They demonstrated that internalised abortion stigma had a direct impact on somatic symptoms moderated by higher levels of stigma-related isolation.

### Qualitative studies

Our meta-aggregated qualitative findings are based on studies that collectively represent the perspectives of approximately 1,958 individuals. Overall, we generated meta-synthesised findings from qualitative studies: (1) experienced stigma, (2) perceived stigma, (3) internalised stigma, (4) anticipated stigma and (5) mitigating factors. The meta-synthesised findings and their ConQual-score are presented in Table 1.

**Table 1.** Qualitative meta-synthesised findings

Meta-synthesised finding	Dependability	Credibility	ConQual	Comments
*1. Experienced abortion stigma:* People seeking abortion care experienced abortion stigma through encounters and events involving healthcare providers, anti-abortion protesters, and members of their social networks. Participants described situations in which they were directly judged, pressured, dismissed, unsupported, harassed or confronted with stigmatising comments and behaviour. These experiences included emotionally upsetting encounters with protesters, negative or distancing reactions from family members or others close to them, and interactions with healthcare providers who were experienced to be rude, judgmental, coercive, or unhelpful. In some cases, healthcare workers created barriers to care by withholding information, delaying referrals, denying support or failing to respond to participants’ emotional and informational needs. Legal and structural conditions also became tangible in participants’ experiences when criminalisation, restricted access, lack of clear information or the need to travel shaped abortion care as isolating, burdensome and morally marked. Together, these findings show that abortion stigma was not only experienced as a general social climate but was lived through specific interactions, institutional practices and structural barriers that directly affected participants’ abortion experiences. • Studies^50,51,54,57–61,63,65,66,69,71,73,75,78,81,83–85,87,90^	Moderate	Moderate	Moderate	Dependability: Downgraded one level. Majority of studies scored 3/5 for 5 criteria. Credibility: Downgraded one level due to a mix of unequivocal (U) and credible (C) findings. U = 37, C = 5
*2. Perceived abortion stigma:* People seeking abortion care perceived abortion as socially, morally, institutionally and legally devalued, even when they did not necessarily report direct verbal attacks or explicit discrimination. This perception was shaped by disapproval in family and social networks, anti-abortion climates in communities and public spaces, stigmatising healthcare and abortion-related services, restrictive legal contexts and the gendered norms of motherhood and sexuality. Participants described their social networks as unsupportive or broadly disapproving of abortion, and some perceived their community and physical environments as saturated with anti-abortion messages. In healthcare contexts, perceived stigma emerged when participants felt criticised, unsupported or not taken seriously by providers, when abortion was framed as an undesirable outcome, or when a lack of information, referral pathways and emotional support made abortion care feel different from ordinary healthcare. Legal and structural conditions also contributed to perceived stigma: secrecy, criminalisation, punitive laws and limited access to information or services created an atmosphere of doubt and conveyed that abortion was morally questionable. Perceived stigma was further shaped by gendered expectations, including the idea that abortion represented a rejection of motherhood or violated cultural norms regarding sexuality and pregnancy outside marriage. Overall, perceived abortion stigma emerged as an awareness of a disapproving social, institutional and legal climate in which abortion was treated as unsupported, morally contested, taboo or deviant behaviour. Studies^52,54,55,58,60,62,63,65,71,81,83^	Moderate	High	Moderate	Dependability: Downgraded one level. Majority of studies scored 3/5 for 5 criteria. Credibility: Downgraded one level due to mix unequivocal (U) and credible (C) findings. U = 17, C = 1
*3. Internalised abortion stigma:* People seeking abortion care frequently internalise stigma, in the form of shame and self-blame, particularly if they have undergone multiple abortions or belong to religious or marginalised communities. They may opt to avoid sharing their decision with others, leading to feelings of isolation and judgment. Misinformation and anti-abortion attitudes further exacerbate their negative emotions. The decision to undergo an abortion often depends on life-stage considerations and sound reasoning. However, stigma and mistrust of medical providers can pose difficulties in this process. Studies^52,58,61,63,66,76,80–82,86,88,90,92^	Moderate	High	Moderate	Dependability: Downgraded one level. The majority of studies scored 3/5 for the 5 criteria. Credibility: Not downgraded due to a majority of unequivocal (U) findings. U = 26, C = 1
*4. Anticipated abortion stigma:* Individuals seeking abortion anticipate stigmatisation and criticism from their communities, families, and HPs. They are anxious about unfavourable reactions, social exclusion and harm to their reputation. Many keep their abortions confidential and feel muted by their loved ones. Stigma is frequently interwoven with religion and law. Certain individuals prefer travelling for the procedure to avoid judgment. Lack of privacy and fear of protesters also serve as obstacles to accessing abortion services Studies^50,53,56,59,60,63,65,69,72,74,77,79,85,92^	Moderate	High	Moderate	Dependability: Downgraded one level. Majority of studies scored 3/5 for 5 criteria. Credibility: Not downgraded due to the majority of findings being unequivocal (U). U = 36, C = 2
*5. Mitigating factors:* People seeking abortion care reported several beneficial factors, including positive interactions with medical personnel, medication abortions to ensure privacy, supportive environments with fellows and the use of telemedicine to sidestep judgment and stigma. Moreover, participants discussed their strategies for coping with religious-based stigma and provided rationales for making the decision to undergo an abortion, such as the well-being of their existing children or their financial stability. Non-judgmental behaviour from HPs and support from family and friends were significant factors. Although there is a possibility of harassment, sharing abortion stories publicly was viewed as a positive experience. Studies^50,54,57,59–61,63,65–67,69–71,76–78,81–83,87,91^	Moderate	High	Moderate	Dependability: Downgraded one level. Majority of studies scored 3/5 for 5 criteria. Credibility: Not downgraded due to a majority of unequivocal (U) and credible (C) findings. U = 65, C = 1

#### Synthesised finding 1: experienced abortion stigma

The first finding was synthesised from six categories, comprising 42 extracts^50,51,54,57–61,63,65,66,69,71,73,75,78,81,83–85,87,90^ that were supported by illustrations representing the participants’ voices (cf. Supplementary Table 2). Individuals encountered direct social judgement and pressure.^63,69^

“*She didn’t want to talk to me, she was just like … I abandoned the baby … She was like, ‘yeah you are, you’re just killing a baby, it’s an innocent child‘, and just saying stuff like that. Saying stuff you wouldn’t just say. And it’s just like,’why would you say that to me?*’”^69^ (p. 5)

Those seeking abortion often faced stigmatising reactions from family and social network,^59–61,63,71,90^ including anti-abortion views,^59,63^ stigma^60,61^ and lack of support.^59–61^ This stigma was linked to religious^61,63,71^ and moral judgment.^59,60,63,71^

“*[God] gives you children because they're a blessing and a gift. You have a gift and you're going to throw it away? Don't kill it and let me adopt it. It'll have our last name … . He or she will know that you're their mom but you won't have to take no [sic] responsibility for it*.”^71^ (p. 112)

Women faced harassment from aggressive^57^ and persistent protesters who followed them, causing feelings of unsafety and stigmatisation.^57,73,81,84^ The protesters used moralising rhetoric.^50^ In one study, some participants noticed the presence of children in crowds^57^ and considered the protesters’ behaviour to be unacceptable.

“*Found it unsettling to hear them repeat phrases like ‘Mommy, I love you, don’t kill me’ and ‘Please don’t kill our friends’. Hearing protestors’ children ask, ‘You would kill me?’*”^57^ (p. 889)

People seeking abortion often encountered judgment^53,65,71,83,85,87^ and stigmatisation^51,58^ from healthcare practitioners (HPs), including negative assumptions about pregnancy and morality.^55,71^ For instance, the studies reported that HPs created barriers (e.g. gatekeeping, withholding information) to abortion services.^54,55,82,83^ Some opted for alternative healthcare options, such as seeking care directly from specialty clinics, owing to negative experiences with traditional medical care.^82^ Additionally, crisis pregnancy centres (CPCs) in the US context^54,65^ contributed to the stigma surrounding abortion and invoked religious judgments.^54^

“*[The doctor] just sort of told me the risks … Like, you know there’s a risk of infertility and all sort of stuff … So I think she was definitely trying to skew me away from it … But to me, that sort of reaffirms this opinion in my head that like, I’d really stuffed up and I was doing a really bad thing. And it just sort of reaffirmed all this negative. So it’s taken me so long to realise that actually what I did wasn’t a bad thing*.”^53^ (p. 3)

Studies also stated that their participants experienced stigma due to legal and structural barriers^55,75,83^ and online and public anti-abortion hostility.^78^

#### Synthesised finding 2: perceived abortion stigma

The second finding was synthesised from five categories, consisting of 18 extracts,^52,54,55,58,60,62,63,65,71,81,83^ supported by illustrations (see Supplementary Table 2).

People seeking abortion care perceived abortion as socially, morally, institutionally and legally wrong, even without experiencing explicit discrimination. In the included studies, this perception was predominantly shaped by disapproval from HP and abortion-related service providers.^54,55,58,62,65,71,81,83^ For instance, they described implicit communication that was perceived as judgemental or insufficient provision of information for the abortion itself or aftercare.

“*Everybody in that clinic was cold […]. It was horrible. From the moment I got there to the moment I left*”.^83^ (p.6)

Further, the criminalised^52,62^ nature and lack of information^62^ contributed to feelings of doubt and deviance around abortion.

“*Yeah, [the community organisation that helped me find services] said where I was going to [have my abortion] was a legal place, but what I was doing was illegal … They said just not to speak too much about it*”.^52^ (p.4)

Further, one study found that their participants perceived disapproval in family and social networks. And the feeling that women can’t make reproductive decisions.^81,82^

“*Out bush – there is still a lot of stigma about getting information in the first place and certainly something that is not talked about … and I reckon there’s a higher rate of teenagers having births because of the stigma.*”^82^ (p.5)

#### Synthesised finding 3: internalised abortion stigma

The second finding was derived from four categories, which encompassed 36 extracts^52,58,61,63,66,76,80–82,86,88,90,92^ supported by illustrations (cf. Supplementary Table 2).

People seeking abortion care frequently experienced internalised shame and guilt, leading to feelings of secrecy and disapproval towards abortions.^63,66,76,80–82,86,88,90,92^ Individuals reported experiencing conflicting emotions such as loneliness^66,88,92^ and self-blame due to moral and religious conflict.^58,63,80,81,90,92^ Abortion stigma played a significant role in the disclosure of their pregnancy status.^52,81,88,92^

“*You go there already you think with some guilt, and you’re blaming yourself*.”^81^ (p. E)

For certain people seeking abortion, religious beliefs^61,63^ or moral obligations^58,76,80,89^ could result in internalised shame and guilt.

“*Because I have a very strong faith in God, I am ashamed. … And that’s something that I have to deal with forever. That’s my biggest fear and regret*.”^63^ (p. 9)

Some women believed that doing multiple abortions is morally wrong, shameful and guilty.^80,85^

“*I guess, for me the thing that I felt sort of weird about was the fact that I had three [abortions]. I have some shame around that, sort of like, you think that I could get it together better on the birth control front*,”^85^ (p. 4)

Single studies found internalised norms of motherhood and femininity,^88^ concerns about one’s own reproductive health^60^ and stigmatising anti-abortion narratives.^65^

#### Synthesised finding 4: anticipated abortion stigma

The third finding synthesises eight categories of 35 findings^50,53,56,59,60,63,65,69,72,74,77,79,85,92^ supported by the illustrations in Supplementary Table 2. Some women expressed anticipation of judgment through HPs^75^ and protestors.^50,56,65,69,77,85^

“*I did think that there would be a lot more judgment and stigma from the medical people that were operating the center*.”^65^ (p. 183)

People seeking abortions often anticipated judgment and stigma from family, partners, and social networks.^50,53,56,58–60,63,65,68,72,79,79,92^ Their concerns included negative reactions,^50,58,59,63,92^ and they tended to value their privacy and secrecy.^53,56,59,63,68,92^ Some women felt silenced by loved ones because of feelings of shame and embarrassment associated with abortion:

“*Just the whole abortion thing is – it’s a touchy subject. It’s embarrassing … First being a Christian, I’m still battling some things like is God mad at me? Like is people gonna be mad at me if they found out? I keep a lot of things to myself anyway, so I knew I wasn’t going to share this*.”^59^ (p. 4)

Some participants feared exposure in their community and loss of privacy,^50,61,65,71^ for instance, due to living in smaller communities.^71^ Moreover, one study explored abortion experiences among women in the US military, revealing anticipated stigma in seeking abortion care due to concerns about judgment in the workplace and the potential impact on their military careers.^64^ Further, one study found that women seeking a second abortion anticipated greater judgment, reporting amplified feelings of shame and concern about negative treatment by healthcare providers based on prior experiences and the perception that a second abortion would be viewed more critically.^85^

#### Synthesised finding 5: mitigating factors

The fourth finding was derived from six categories encompassing 66 extracts^50,54,57,59–61,63,65–67,69–71,76–78,81–83,87,91^ supported by illustrations (see Supplementary Table 2).

Some participants received beneficial support from their social networks^59,60,69^ and openness^70,73,76–78,81^ regarding abortion, which might help to minimise outcomes such as stigma^65,70^ and shame.^59,73^ One study included women who shared their abortion experiences publicly, which relieved their feelings of shame or guilt.^70^

“*The first time I shared my abortion story on stage, I had women come up to me crying and hugging me. Several of them had never told anyone before that they had an abortion previously because they were too ashamed or felt guilty. They said my story helped them realise that they didn't need to feel shame or guilt*.”^70^ (p. 4)

Participants experienced the provision of non-judgmental^54,65,67,82,83,87^ and supportive care by HPs and services.^54,65,69,71,82,83,87^ For instance, they felt validated in their decision,^65,71^ experienced empathy^67,87^ and kind language by HPs.^50,87^

“*I had a doctor sitting across from me who was very understanding and very warm in her communication and then that was really only very pleasant for me. Because of that warmth and the amount of information, I could immediately make a well-considered choice*.”^50^ (p. 168)

Some studies suggest that contextualising one's abortion experience can aid in the decision-making process.^54,63,76^ For example, women's decisions to have abortions were influenced by financial concerns, poor health^76^ and existing children.^63,76^

“*I don’t really agree with abortion, because I personally do feel somewhat like it’s slight murder. But on the other hand, you have people who, you know, it might come in handy for. … You have people like me. I just had a baby, and I just can’t do another one right now*.”^63^ (p. 8)

Religion, which can heighten stigma in some circumstances, could play the opposite role for others.^54,61,69^ In their study on the management of religion and morals in the abortion process, Frohwirth et al. explored various strategies to cope with religious-based abortion stigma, such as personal exceptionalism, rejecting religion and revising beliefs.^61^

“*I’m pro-choice because I don’t feel like my decisions and my viewpoints on the world should have any impact on anybody else’s decisions*.”^54^ (p. 633)

“*You know blah, blah. I’m like, ‘Well, I’m sure I can work that out with God’. … There’s various reasons why I’m choosing this decision I have thought about, this is God’s plan, it was meant to be like this.*”^69^ (p. 5)

Furthermore, one study revealed that structural factors, such as distance from abortion protesters, supported their abortion process.^57^ Lastly, teleabortion was considered helpful in maintaining privacy by some women.^71,91^

### Integration of quantitative and qualitative evidence

A summary of the qualitative and quantitative findings is shown in Figure 3. Our review identified evidence on perceived, internalised and anticipated abortion stigma. Through this analysis, we identified structural and behavioural barriers that are interconnected with abortion stigma among people seeking abortion care. Moreover, qualitative studies have examined the potential factors that may mitigate abortion stigma. Social, psychological and acess-related consequences are the potential outcomes of stigma.

**Figure 3.**
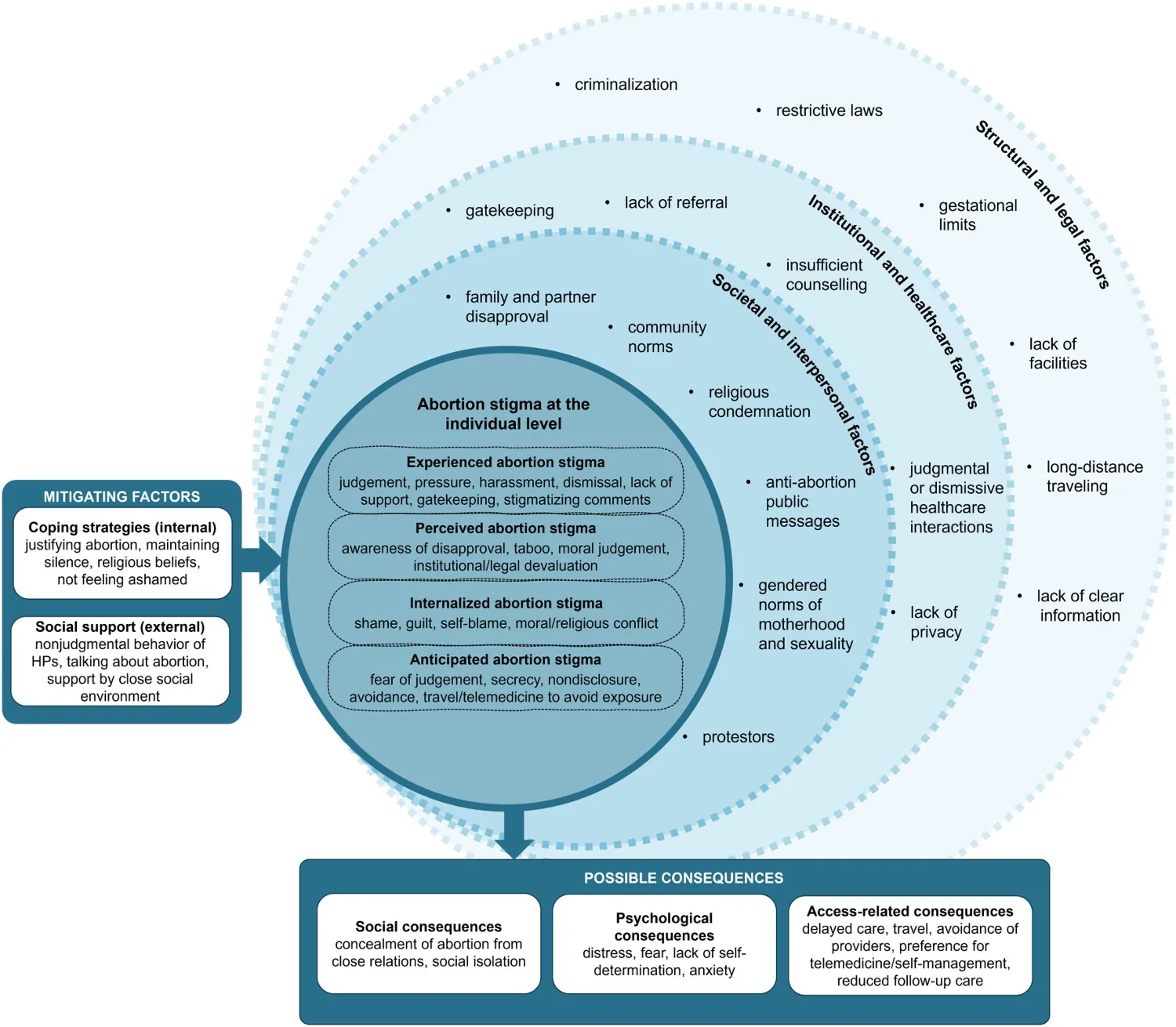
Working framework of abortion stigma

Figure 3 presents the framework of abortion stigma among people seeking abortion care based on our mixed-methods systematic review. It identifies experienced, perceived, anticipated and internalised stigma at the individual level, as well as societal, interpersonal, institutional, healthcare, structural and legal factors contributing to stigma. The consequences include social, psychological and access-related effects. Additionally, the framework synthesises internal and external mitigating factors identified in the literature.

#### Supportive evidence

Both empirical strands have investigated the experience of abortion stigma among people seeking abortion services. Social support appears to be crucial in reducing the effects of abortion stigma, as evidenced by a quantitative study that demonstrated potential benefits for intimate partners. Qualitative studies have demonstrated that having supportive individuals in one's close social environment and engaging in open communication about the topic of abortion can be effective in reducing stigma. Both quantitative and qualitative research have provided evidence of internalised judgment towards people seeking abortions.

#### Contradictory evidence

Comparing qualitative and quantitative findings, it seems that quantitative research tends to depict a more negative perspective on the stigma associated with abortions. This discrepancy might be attributed not only to the experiences of participants but also to inherent differences in sampling methods and research techniques. Quantitative research often relies on larger samples from clinics and employs standardised measures of stigma, which can highlight clinical intricacies and psychosocial challenges. Moreover, the instruments commonly used in quantitative research are designed to detect and measure negative experiences of stigma and often do not include questions about neutral or positive experiences, which may skew the overall picture. On the other hand, qualitative research typically involves smaller, intentionally selected groups and aims to delve into detailed, context-specific experiences that may highlight personal agency and resilience, even in stigmatising settings. Therefore, when interpreting stigma across different research methodologies, it is important to proceed with caution and acknowledge these structural and epistemological differences.

Furthermore, the meta-aggregation provided first reflections on the societal and structural factors of abortion stigma (cf. Figure 3), whereas quantitative studies did not. Subsequently, there is an empirical void concerning the structural and societal drivers of abortion stigma. The outcomes of qualitative studies provide initial leads for such an exploration.

Religion was linked to abortion stigma in the quantitative studies. However, qualitative studies suggest that religion could potentially serve as both a hindrance to and a facilitator of abortion stigma. One quantitative study^40^ demonstrated that Black people experience less abortion stigma than White people do. However, quantitative studies might not be able to capture this aspect of abortion stigma and are dependent on the context and the population under study. This study also lacked an intersectional analysis.

## Discussion

In this mixed-methods systematic review, we focused on abortion-related stigma among people seeking abortion care. Most of the studies were conducted in the USA, followed by England and Australia. There is a notable deficiency in the theoretical conceptualisation of abortion stigma, with a lack of consistency in the definitions used (RQ1). The predominant use of Kumar, Hessini, and Mitchell's definition^93^ reflects a view of stigma as an attribute, but there is a call to incorporate definitions that recognise abortion stigma as a social process,^9^ and as a mechanism of social control embedded in power relations into theoretical concepts.

We aimed to assess to what extent people seeking abortion care experience abortion stigma in HICs from a quantitative perspective (RQ2). We showed that various measurement methods were employed in studies that investigated abortion stigma, including ILAS scores, single-item measures, or self-developed items, which complicates comparisons of its prevalence at a population level. Studies that used ILAS were comparable to a limited extent, as some of them used only specific subscales or single items of the scale and investigated convenience samples and varying outcome variables. Abortion stigma is affected by factors such as abortion history, decision-making processes, and pregnancy characteristics. Associations exist between higher gestational age and the healthy development of the fetus, resulting in increased felt stigma surrounding abortions. Sociodemographic factors, such as marital status and religious affiliation, are linked with abortion stigma. Abortion stigma is associated with negative mental health outcomes; however, intimate partner support can potentially mitigate these effects. Further research is necessary to understand the impact of exposure to anti-abortion protesters.

From an intersectional perspective, these findings are consistent with existing literature. Garnsey et al.^97^ and Moore et al.^98^ demonstrate how economic hardship can compound abortion stigma, particularly when individuals lack the financial resources or social support to access care. Although excluded from the present review due to sampling limitations, one study by Mosley et al.^99^ explicitly explored intersectional experiences of stigma among Black and Latinx women, illustrating how abortion stigma intersected with racial and gendered forms of marginalisation. In addition, qualitative research from the US shows how young people experienced layered stigma: both for being pregnant at a young age and for seeking an abortion.^100^ Although the studies included in this review often collected or reported variables such as ethnicity, socioeconomic background and age, these factors were rarely analysed through an explicitly intersectional lens, revealing a significant gap in intersectional analysis across the body of research. Strong et al.^11^ have further criticised the abortion stigma literature for lacking intersectional measures, overlooking the macro level. They advocate for a framework that considers stigma not only at the individual and interpersonal levels but also at the structural and societal levels, where race, class, age and gender intersect. Future research should move beyond basic subgroup comparisons to adopt truly intersectional approaches that reflect the complex realities of abortion stigma. Also, employing a reproductive justice framework might broaden understandings of stigma and discrimination by highlighting how intersecting laws, policies, and institutional systems shape access to safe, respectful and equitable sexual and reproductive health services.^101^

We synthesised four qualitative findings from our meta-aggregation (RQ3): (1) perceived abortion stigma, (2) anticipated abortion stigma, (3) internalised abortion stigma and (4) mitigating factors. People seeking abortion care experience stigma from multiple sources, such as HPs, anti-abortion protesters and their social networks. Anti-abortion activists, online communities, and HPs subject abortion to judgment and stigmatisation, perpetuating the taboo. Anticipation and internalisation of such stigma led to unease, fear of negative responses, social isolation and damage to the public image among people seeking abortions. Mitigating factors included positive interactions with HPs, medication-induced abortions to maintain confidentiality, supportive environments and the use of telemedicine to avoid judgment and stigma.

Applying a sexual and reproductive justice framework, these results illustrate that abortion stigma is not simply a personal or interpersonal experience but a manifestation of intersecting structural inequalities. The criminalisation of abortion, provider gatekeeping, lack of access to accurate information, judgment from healthcare professionals, and cultural or religious condemnation reveal how systems of power and policy intersect to restrict access to safe, respectful, and equitable reproductive healthcare. People seeking abortion care are not a homogeneous group; their stigma experiences vary significantly depending on their contexts.^11^ In this way, stigma reflects and reproduces systemic injustices rather than being an individual failing or fixed condition.^9^ It is contextual, contested and embedded in broader societal hierarchies. At the same time, mitigating factors, such as non-judgmental care, social support and telemedicine, demonstrate how reproductive autonomy can be protected when abortion is delivered in supportive environments that respect individual decision-making and dignity.

### Implications for future research and measurement?

Hanschmidt^18^ examined stigma among abortion seekers, HPs, and the general public. Since 2016, the number of publications has markedly increased. This review concentrated solely on people seeking abortion care; however, a second review will address HPs and the public. Following the findings of Hanschmidt et al.,^18^ it can be stated that there is a deficiency in the theoretical conceptualisation of abortion stigma specifically. The studies that refer to definitions tend to utilise Kumar, Hessini and Mitchell's^93^ definition, which views stigma as an attribute. It is necessary to incorporate definitions of abortion stigma as a social process under the influence of exercised power^9^ into theoretical concepts. Only Mackleff et al. used a definition of abortion stigma as “a social process that reproduces unequal power relations”,^83^ referring to Millar.^9^ This also sets the tone for the theoretical conceptualisation and the goal of the study, which incorporates the “influence of macro-level abortion stigma and other structural factors”.^83^ Broussard also focuses on structural stigma; however, he only defines structural stigma itself and not structural abortion stigma.^91^ Many qualitative research studies do not provide an exact definition of abortion stigma. This can be problematic because it is unclear what theoretical considerations were at the heart of the study's conceptualisation and analysis. However, qualitative studies may have omitted a definition of abortion stigma since it was not their primary focus.

There has been limited progress in the development of suitable measurement tools for quantitative research. In our research, two studies employed modified (unvalidated) tools from the 1990s, while the others used the ILAS score or two subscales, such as in the review published in 2016. This is also in line with the results of a recent systematic review and meta-analysis by Ratcliffe and colleagues, in which they stress the need for improved conceptualisation and measurement of structural abortion stigma.^102^

In keeping with the findings of 2016,^18^ our research revealed that people seeking abortion care face stigma in various forms, including physicians, social circles, protestors, friends and family. Several qualitative studies described that participants perceive stigma surrounding abortion and experience fear of judgment. They also internalise feelings of self-blame, self-judgment and selfishness. In our comprehensive study, we uncovered mitigating factors related to the stigma surrounding abortion that were not previously reported by Hanschmidt et al.^18^ Our research revealed that the stigma of abortion is a real occurrence among people who seek abortion, but that there are ways to counteract it. Few studies have investigated mitigating factors as their primary research objective, but our findings suggest that this is an area with potential for future investigation.

Most quantitative studies have examined the association between mental health and abortion stigma. It is imperative to conduct more studies that examine mental health issues integrally, considering the duration of the symptoms and their relationship to the life circumstances of the respective person. Such research could provide more information on how mental health problems are associated with abortion and abortion stigma. Thus, future research should also address pathways of abortion stigma among people seeking abortion care by clarifying whether it is a potential cause or consequence of factors like psychological distress, somatic symptoms, or other negative psychosocial and health-related factors (which can be derived only limitedly from single measurements). This is crucial to provide beneficial psychosocial support within the context of abortion seeking, but also in general mental health care.

It is imperative to conduct longitudinal and mixed-method studies to further assess the strength and generalisability of this association. Additionally, quantitative studies should consider a wider array of determinants. To complement this with qualitative research, it is also necessary to conduct biographical interviews. The study findings indicate the need for more research that adopts intersectional methodologies to scrutinise abortion stigma as a social phenomenon. For instance, studies could consider the link between gender inequality/inequity^103^ and abortion as a potential driver of abortion stigma.^9^ In addition, we were only able to include three studies not focusing solely on cisgender women in this review. Future studies that are gender-inclusive and seek to include the experiences of persons other than cisgender women would be welcome. These individuals are likely to face even more stigmatisation.

Quantitative research should focus on a wider range of outcomes as well as mitigating factors, as they can be beneficial resources within the decision-making process as well as the prevention and reduction of abortion stigma, and compare them based on, for instance, the ILAS scores.^104^

Implementing these ideas would not only increase the validity of abortion stigma research but also provide concrete recommendations for professionals providing counselling and medical care to persons seeking abortion care.

### Strengths and limitations

The strength of this review lies in its use of MMSR to provide a thorough and detailed examination of both quantitative and qualitative data, effectively elucidating the effects and experiences associated with abortion stigma. Our research also provides a comprehensive overview of the existing research. However, this review has several limitations.

#### Critical appraisal of inclusion criteria

We only included peer-reviewed articles, which means that we may have missed experiences related to abortion stigma. We further limited the scope of this review to high-income countries, so our results may not apply to other contexts. Future research should therefore review current studies on abortion-related stigma in low- and middle-income countries.

#### Critical appraisal of qualitative studies

Describing the theoretical and methodological grounding as well as reflexivity and positionality are part of rigour in critical qualitative (health) research,^105,106^ which most included studies lacked. In the context of abortion stigma research, this can be especially problematic, as factors such as religious beliefs might influence how research questions, methods, and findings are constructed and interpreted.^107^ Reasons for not reporting might be the restricted word limit in journals in healthcare research (e.g. psychology, medicine and epidemiology).^105^ Additionally, most articles employed thematic or qualitative content analysis and techniques associated with qualitative descriptive approaches.^108^ Studies utilising qualitative descriptive methodology in healthcare research frequently fail to specify philosophical or methodological models.^109^ However, such specifications can enhance the rigour and reliability of research.^108,110^

#### Critical appraisal of quantitative measurements

In this review, we were able to apply our theoretical framework of abortion stigma, including the differentiation between experienced, perceived, internalised and anticipated stigma, to the qualitative findings and categorise them accordingly. However, this categorisation was more limited in quantitative studies, as the available instruments did not consistently or clearly distinguish between these different forms of stigma. Although the ILAS captures internalised stigma through the *self-judgment* subscale and anticipated stigma through the *worries about judgment* subscale, the remaining subscales, isolation and community condemnation, are less clearly attributable to a specific stigma form. This limits direct comparisons between qualitative and quantitative findings and highlights the need for more precise measurement tools.

#### Critical appraisal of methodology

It is also important to note that the meta-aggregation process relies on subjective categorisation, which may be influenced by personal experiences, opinions and perspectives. Lastly, the method of meta-aggregation has been critiqued for its process of generalisation because the results of qualitative studies have already been synthesised from the qualitative data. This abstraction distances the aggregated findings even more from the original context.^111^

This review underscores the value of a more detailed and differentiated reporting of study samples in future research. In several cases, it was not always clear whether participants reflected on their personal experiences of abortion and abortion-related stigma, or whether they spoke more generally about abortion and public stigma. For instance, two studies^100,112^ that were not included in the final analysis involved members of the public, some of whom had abortion experiences; however, neither study clearly distinguished in their results that participants had personally obtained an abortion. Greater clarity regarding participants’ abortion history and their relationship with abortion-related stigma would strengthen the interpretability and applicability of findings in future studies.

## Conclusion

Abortion stigma among people seeking abortion care is a dynamic social process deeply intertwined with legal, cultural and social factors that shape individuals’ experiences and access to care. Understanding stigma in this context is essential for developing effective support and interventions that address its structural and interpersonal dimensions. Future research should apply clearer conceptual distinctions between experienced, perceived, internalised and anticipated stigma to better capture how different forms of stigma operate and affect abortion care.

## Supplementary Material

Supplementary Table 2_revised_.docx

Supplementary Table 4_.docx

Supplementary Table 5_.docx

Supplementary Table 7_revised.docx

Supplementary Table 1_.docx

Supplementary Table 3_.docx

Supplementary Table 6_.docx

## Footnotes

† The Joanna Briggs Institute's Guidelines for Evidence Synthesis provide thorough instructions for the authors working on systematic reviews.

‡ The JBI-Qualitative Assessment and Review Instrument (JBI-QARI), a Critical Appraisal Checklist for interpretive and critical research was used to evaluate the qualitative paper.

§ The ConQual approach, or "summary of findings," is a structured method for assessing confidence in qualitative research outcomes based on the research type, study dependability and credibility of the findings
