# Supplementary material for: Abortion stigma among people seeking abortion care in high-income countries: a mixed-methods systematic review
Source: Sex Reprod Health Matters. 2026 Jul 27;33(1):2709964. doi: 10.1080/26410397.2026.2709964 (PMC13660548; doi:10.1080/26410397.2026.2709964)
Supplement: Supplementary file 1 [file ZRHM_A_2709964_SM9487.docx]

Supplementary Table 6. Abortion stigma definitions

| **Citation** | **Explicitly stated self-**  **definition** | **Cockrill and Nack (2013)** | **Hanschmidt et al. (2016)** | **Herek (2009)** | **Kumar, Hessini and Mitchell (2009)** | **Millar (2020)** | **Norris et al. (2011)** | **Generic stigma definition** | **Not**  **explicitly stated** |
| --- | --- | --- | --- | --- | --- | --- | --- | --- | --- |
| **Quantitative Studies** | | | | | | | | | |
| Biggs et al. (2020) |  |  |  |  |  |  |  |  |  |
| Chesnokova et al. (2024) |  |  |  |  |  |  |  |  |  |
| Hanschmidt et al. (2018) |  |  |  |  |  |  |  |  |  |
| Hanschmidt et al. (2017) |  |  |  |  |  |  |  |  |  |
| Kerns et al. (2022) |  |  |  |  |  |  |  |  |  |
| Metha et al. (2019) |  |  |  |  |  |  |  |  |  |
| O’Donnell et al. (2018) |  |  |  |  |  |  |  |  |  |
| Sonalkar et al. (2017) |  |  |  |  |  |  |  |  |  |
| Steinberg et al. (2016) |  |  |  |  |  |  |  |  |  |
| **Qualitative Studies** | | | | | | | | | |
| Aiken et al. (2018) |  |  |  |  |  |  |  |  |  |
| Aiken et al. (2017) |  |  |  |  |  |  |  |  |  |
| Altshuler et al. (2017) |  |  |  |  |  |  |  |  |  |
| Beynon-Jones (2017) |  |  |  |  |  |  |  |  |  |
| Broussard (2020) |  |  |  |  |  |  |  |  |  |
| Broussard (2024) |  |  |  |  |  |  |  |  |  |
| Carroll et al. (2022) |  |  |  |  |  |  |  |  |  |
| Cashman et al. (2021) |  |  |  |  |  |  |  |  |  |
| Chandrasekaran et al. (2023) |  |  |  |  |  |  |  |  |  |
| Chor et al. (2019) |  |  |  |  |  |  |  |  |  |
| Dickey (2022) |  |  |  |  |  |  |  |  |  |
| Doran & Hornibrook (2016) |  |  |  |  |  |  |  |  |  |
| Foster et al. (2020) |  |  |  |  |  |  |  |  |  |
| Frohwirth et al. (2018) |  |  |  |  |  |  |  |  |  |
| Gelman et al. (2017) |  |  |  |  |  |  |  |  |  |
| Grindlay et al. (2017) |  |  |  |  |  |  |  |  |  |
| Hatcher et al. (2018) |  |  |  |  |  |  |  |  |  |
| Heller et al. (2016) |  |  |  |  |  |  |  |  |  |
| Hermannsdóttir (2022) |  |  |  |  |  |  |  |  |  |
| **Citation** | **Explicitly stated self-**  **definition** | **Cockrill and Nack (2013)** | **Hanschmidt et al. (2016)** | **Herek (2009)** | **Kumar, Hessini and Mitchell (2009)** | **Millar (2020)** | **Norris et al. (2011)** | **Generic stigma definition** | **Not**  **explicitly stated** |
| Hoggart et al. (2017) |  |  |  |  |  |  |  |  |  |
| Hoggart (2017) |  |  |  |  |  |  |  |  |  |
| Holten et al. (2021) |  |  |  |  |  |  |  |  |  |
| Holten et al. (2023) |  |  |  |  |  |  |  |  |  |
| Hutchens (2023) |  |  |  |  |  |  |  |  |  |
| Ireland et al. (2020) |  |  |  |  |  |  |  |  |  |
| Kavanaugh et al. (2019) |  |  |  |  |  |  |  |  |  |
| Kerestes et al. (2021) |  |  |  |  |  |  |  |  |  |
| Kerestes et al. (2022) |  |  |  |  |  |  |  |  |  |
| Killinger et al. (2022) |  |  |  |  |  |  |  |  |  |
| Kimport & Rasidjan (2023) |  |  |  |  |  |  |  |  |  |
| Kimport (2019) |  |  |  |  |  |  |  |  |  |
| LaRoche & Foster (2018) |  |  |  |  |  |  |  |  |  |
| LaRoche & Foster (2020) |  |  |  |  |  |  |  |  |  |
| LaRoche & Foster (2021) |  |  |  |  |  |  |  |  |  |
| Lerma et al. (2023 |  |  |  |  |  |  |  |  |  |
| Makleff et al. (2023) |  |  |  |  |  |  |  |  |  |
| Mahanaimy et al. (2022) |  |  |  |  |  |  |  |  |  |
| Margo et al. (2019) |  |  |  |  |  |  |  |  |  |
| Monchalin et al. (2023) |  |  |  |  |  |  |  |  |  |
| Purcell et al. (2020) |  |  |  |  |  |  |  |  |  |
| Rafferty & Longbons (2021) |  |  |  |  |  |  |  |  |  |
| Warren et al. (2022) |  |  |  |  |  |  |  |  |  |
| Wilson-Lowe (2024) |  |  |  |  |  |  |  |  |  |
| Woodruff et al. (2020) |  |  |  |  |  |  |  |  |  |
