# Supplementary material for: Abortion stigma among people seeking abortion care in high-income countries: a mixed-methods systematic review
Source: Sex Reprod Health Matters. 2026 Jul 27;33(1):2709964. doi: 10.1080/26410397.2026.2709964 (PMC13660548; doi:10.1080/26410397.2026.2709964)
Supplement: Supplementary file 2 [file ZRHM_A_2709964_SM9488.docx]

Supplementary Table 3. Reflexivity statement.

| **Researcher** | **Statement** |
| --- | --- |
| Jana Niemann, MSc (European Public Health) | She identifies as a white, cisgender woman and mother. She conducts sexual and reproductive health research through a human rights and critical feminist lens. Her academic background is public health and social epidemiology, with methodological training in both quantitative and qualitative approaches. While she had prior experience in conducting reviews of quantitative studies, this project marked her first engagement with qualitative meta-aggregation. |
| Marie Bernard, PhD (Medical Sciences) | She is a white, cisgender woman and mother. Trained as a sociologist with a methodological focus on quantitative research, her work has centered on the stigma of chronic diseases and their consequences. She has also researched the determinants of stigmatizing attitudes, bringing this expertise to bear in the context of abortion stigma. This project offered an opportunity to critically reflect on how such attitudes form and manifest in the domain of reproductive health, and to contribute to a more nuanced understanding of stigma as a structural and relational phenomenon. |
| Dennis Jepsen, MA (Therapeutic Social Work) | DJ identifies as a white, cisgender man who conducts research on intimate partner violence, posttraumatic stress and psychiatric disease from a human rights and social injustice perspective. His academic background is therapeutic social work, using predominantly a quantitative approach. |
| Nadja Freymüller, MSc (European Public Health) | She is a white, cisgender woman with an academic background in Public Health. She primarily conducts qualitative research and focuses on rehabilitative care and care experiences, particularly those of people with disabilities. |
| Laura Weinhold, MSc (Nutritional Sciences) | She identifies as a white, cisgender woman with an academic background in nutritional science. Her experience lies in quantitative and qualitative research methods, and this project signified her inaugural engagement with qualitative meta-aggregation. |
| Céline Miani, Prof (Gender Epidemiology) | CM is a white, cisgender woman and mother. Her research focuses on gender norms and relations and their impact on reproductive health and healthcare (e.g., contraception, birth, abortion). In her work, she uses a feminist, critical approach to social epidemiology. |
| Claudia Luck-Sikorski, Prof | CLS is a psychologist and psychotherapist by training. She has researched the stigma of chronic diseases but has yet to reflect on stigmatized behaviors such as the decision for abortion. As a therapist, she acknowledges the potential burden of a grave decision in particularly if it is affected by anticipated and felt stigmatization. Documenting the nature of stigmatization, therefore, felt like a necessary prerequisite to raise awareness among therapists dealing with patients who experience suffering after an abortion. |
