# Supplementary material for: Abortion stigma among people seeking abortion care in high-income countries: a mixed-methods systematic review
Source: Sex Reprod Health Matters. 2026 Jul 27;33(1):2709964. doi: 10.1080/26410397.2026.2709964 (PMC13660548; doi:10.1080/26410397.2026.2709964)
Supplement: Supplementary file 3 [file ZRHM_A_2709964_SM9489.docx]

Supplementary Table 1. Findings of the included qualitative studies.

| **Study: Aiken *et al. (2018).* Experiences of women in Ireland who accessed abortion by travelling abroad or by using abortion medication at home: a qualitative study** | |
| --- | --- |
| Finding | Stigma engendered by the law. (C) |
| Illustration | “The whole way people think about abortion in Ireland stems back to it being illegal. Abortion is illegal, so it’s automatically bad. My parents are not even that religious. But they’re from Ireland, they’re going on 60 and that’s the world that they’ve grown up in.” ^(p.6)^  “I had to illegally have an abortion in my own country, and it’s not right that I had to do that at 19 on my own without telling anyone out of fear of either being arrested or even just being judged. I don’t think I will ever tell anyone about it because of the stigma around it.” ^(p.6)^ |
| **Study: Aiken *et al. (2017).* Barriers to accessing abortion services and perspectives on using mifepristone and misoprostol at home in Great Britain** | |
| Finding | Many women wished to keep their abortion secret because of either perceived or experienced stigma around abortion (U). |
| Illustration | "I’m ashamed and embarrassed to return to clinic as I’ve been for an abortion before and know I will be judged for having another one. The stigma of having to walk into any face-to-face setting is too much.” ^(p.5)^ |
| **Study: Altshuler *et al. (2017).* A good abortion experience: A qualitative exploration of women's needs and preferences in clinical care** | |
| Finding | Although the doctor did not actually tell her she was “nothing,” she felt that his behavior was judgmental and dehumanizing. (U) |
| Illustration | “I felt like the doctor was judging the person, my character … he didn't treat me like a person, an individual. He treated me like, ‘Get on up on the table. Let's get this over with because you ain't  nothing.’ You know, ‘Look at you … you're paying $500 to get rid of something you made.’ That's the way I felt …. At least [he could have] assured me that he know[sic] that what I'm going through is not easy. I think that's what I was looking for.” ^(p.112)^ |
| Finding | The doctor’s negative response to her age and the lack of compassionate care led her to conclude that she was, indeed, doing something morally wrong. (U) |
| Illustration | “[The doctor] looks at me and he looks at my chart and he's like, “How old are you?” And I was like, “15.” He's like, “Wow.” That was just like a horrible experience. It was already a bad enough day, and what an insensitive thing for a doctor to say to some-one, a young girl who's obviously already completely like freaked out and upset …. Nobody at any time did or said any-thing that made me feel like [having an abortion] was okay, like other women go through this, like you're not a bad person. It was just the opposite. I felt judged … felt like everything I was doing was wrong.” ^(p.112)^ |
| Finding | Sofia's parents pleaded with her to allow them to adopt this potential child, as she recounted. (U) |
| Illustration | “[God] gives you children because they're a blessing and a gift. You have a gift and you're going to  throw it away? Don't kill it and let me adopt it. It'll have our last name …. He or she will know that you're their mom but you won't have to take no [sic] responsibility for it.” ^(p.112)^ |
| Finding | Sofia was grateful that her decision to end the pregnancy was validated and understood as moral by her providers. (U) |
| Illustration | “They didn't make you feel bad that you were doing it …. They were like supporting you.” ^(p.112)^  “You're not doing a horrible thing. I know why you're doing it …. Do not feel bad. Like this is for your life, to better your life.” ^(p.112)^ |
| Finding | Helpful interaction with a medical assistant prior to the abortion. (U) |
| Illustration | “I was crying and [the medical assistant] was just like, ‘Don't, you're not doing anything wrong …. You're not a bad person.’ And I had even told her … ‘I have a kid and I can't believe I'm doing this.’” ^(p.112)^ |
| Finding | Feeling judged outside the abortion clinic by anti-abortion protesters and she continued to feel uncomfortable after she entered the clinic. (U) |
| Illustration | “It's an abortion clinic, so you go there and you know that that person is getting an abortion … From the time you make it there … I was pretty embarrassed by it, because I walked into the clinic and I feel like all eyes on me, and I feel like everybody knows … and who knows what they're thinking …. it's some stuff that I'm very discreet about …. So, when … those types of situations arise, I just don't like that feeling … there's no privacy at all.” ^(p.114)^ |
| Finding | Living in smaller communities often posed the risk of seeing familiar people, which compounded fears of judgment. (U) |
| Illustration | “It's embarrassing. Because … I knew a lot of people in there …. And I feel irresponsible. Because that's something you want to be discreet, not [involve] everybody in the neighborhood.” ^(p.114)^ |
| Finding | To decrease the possibility of feeling exposed, some participants […] had a medication abortion instead of a procedure. (U) |
| Illustration | “I don't want anyone to know about this, besides the person that gives me the pill. And I want to  take the pill, go home and be alone …. I was so ashamed of everything going on, that I had even gotten pregnant.” ^(p.114)^ |
| Finding | They described the supportive environment created when women could all be together in one room, before and after the abortion. (U) |
| Illustration | “I felt that I had a support group …. I'd rather be in the room with other people that's[sic] going through the same thing …. I think I would've been uneasy just laying there in the room by myself  and thinking about what I just done. So, with the other ladies in the room with me … helped me through that process.” ^(p.114)^ |
| **Study: Beynon-Jones (2017). Untroubling abortion: a discourse analysis of women’s accounts** | |
| Finding | Women position themselves as making a personal choice/decision, which does not impact on the agency of other people. (U) |
| Illustration | "... [a friend] told a lot of people so now I’m getting a lot of stick [...] for it. That’s probably the only reason why I said I’m definitely doing this interview now is just because I’ve had so much stick for my decisions and it’s got to a point now where I’m getting so infuriated with how people react to abortion. It’s just like, it was my personal choice. It’s not anything that you had to do. I didn’t force you to have an abortion of your child or anything like that so there’s no reason I should get people’s hate." ^(p. 233-234)^  "My [relative] isn’t supportive of it [abortion] but at least he turned round and said, ‘‘If it’s the right decision for you then I will support you.’’ [...] He’s done it the way that I’d want. He’s not said that he’s supportive of my decision but he’s supportive of me. Whereas she’s [another relative] done it in the way that, ‘‘No, this is wrong.’’ [...] I will never speak to her again for what she said to me." ^(p.234)^ |
| Finding | Navigating anti-abortion protests outside of clinics. (U) |
| Illustration | " I just kind of like doing it I guess. I think it helps. And it’s like really annoying when those people stand outside the clinic all the time. At the [clinic] there was the people that are really against abortion. And I just don’t think – like obviously they’re Christian or religious - but I don’t think they really have the right to be there because obviously at the end of the day it’s your choice. I think that kind of made me do it because I was like you don’t really understand the reasons why people do it. It’s not like I got pregnant and then just decided, ‘‘Oh yeah, I’ll just have an abortion for fun sort of thing.’’ That kind of annoyed me which is why I also think I took part because I don’t think that you should give people who have terminations a bad name. I don’t think that’s really fair because you don’t understand other people’s circum-stances. [...] I think the worst thing is that obviously it’s going to be a hard decision for some people anyway. I just don’t think – like there should be, I don’t know, some rule against it and they shouldn’t – like obviously I don’t mind if they want to express their views but not outside the place where it’s going to happen. And especially if  you’ve got the pictures like the [inaudible] weeks and everything, that doesn’t help either. They’re there constantly throughout the day and then they were there the next day as well and I was like I just don’t want to deal with this really.” ^(p.234)^ |
| Finding | Openness about abortion is positioned as entirely possible within the context of close friendships, but it is not something that can reasonably be mentioned in casual interaction. (U) |
| Illustration | "Sian: And is it something that you sort of talk about with other people, your experiences of it or?  Participant: Yeah, I’ve talked about it with probably three of my closest friends but not - maybe four even – but not, not flippantly. But I have shared my experiences with friends, yeah.  Siaˆn: Yeah, ok. So when you say not flippantly do you mean sort of it’s something that you’d have a more of an in-depth conversation about as opposed to something you would mention in passing kind of thing?  Participant: Yeah. No, ‘‘Oh yeah, I had one of those,’’ [laughs], ‘‘Oh I had two of them in fact!’’ [parody] More like I guess a couple of them – how did I talk about it actually? I think because I’m at an age where we talk about children and maybe in conversations I guess it came up where like, ‘‘Yes, I’ve been pregnant and I’ve had an abortion.” ^(p.236)^ |
| Finding | Women’s positioning of themselves as ‘‘open’’ has to be carefully navigated in relation to admissions of non-disclosure in which they risk positioning themselves as ‘‘ashamed’’ or ‘‘embar-rassed.’’. (U) |
| Illustration | "I wouldn’t talk about it at a dinner party but if I’m with some close girlfriends and it comes up then I’ll. Because it’s not something I’m embarrassed about, I think people understand the situation, but you obviously gauge your crowd and you know the reactions that you would want to, you know." ^(p.236)^  "Interviewer: [...] is it something you talk to other people about or? Participant: I think I told maybe sort of friends-wise only maybe one or two people. And if it comes up in conversation normally I just kind of say that we lost the baby. But then that kind of - I feel a bit ashamed to lie about it but I’m not lying it’s just, it’s such a big conversation to have and to try and explain that I just tend to say that we just lost the baby. But I think my gut instinct is that I would just say but it’s just that it’s such a big conversation. And then like we said, it’s the timing. If your friend is pregnant it’s not a conversation that you’re going to enter into." ^(p.236-237)^ |
| **Study: Broussard (2020). The changing landscape of abortion care: embodied experiences of structural stigma in the Republic of Ireland and Northern Ireland** | |
| Finding | Apprehension at “the thought of having to go to a clinic and speak to a nurse.” (C) |
| Illustration | "For me, there’s something appealing about the online process, and being at home… I didn’t really want anybody helping me, or comforting me, particularly someone I didn’t know. The idea of actually being able to be on your own and just get it done was appealing to me. " ^(p.12)^ |
| Finding | Found that telemedicine offered support and information while simultaneously protecting her from judgment and social stigma. (U) |
| Illustration | "I liked the way they [WoW] were supportive, that you could talk to them, you could send them an email whenever you wanted. And you’re not physically talking to some person face to face, so they don’t know what you look like or who you are, you don’t feel judged the way that you do when it’s face to face." ^(p.12)^ |
| **Study: Broussard (2024). Strategic (Non)Disclosure: Activation and Avoidance of Social Ties among Women** | |
| Finding | For these women, nondisclosure was a tool to manage stigma and prevent what they perceived to be irreparable damage to the most important relationships in their lives. |
| Illustration | “[My mother] told me that if one day I decided to do that, I had to forget that I had a family and that I had her, like, ‘If you do that, I’m dead to you.’ So, the fear of that was what was instilled in my mind.” ^(p.12)^  “I didn’t want to...it’s something that I’m going to take to the grave....I’m the little girl of the house, the young-est. There would always be a shadow over me for telling them this.” ^(p.12)^  “The thing is, nobody knew it was an abortion. I said that it was my appendix removed....So yes, I felt supported because there were people around taking care of me and showing care. Even the bad guy [her abusive partner] showed up. He was in the honeymoon phase! It was good” ^(p.12)^ |
| **Study: Carroll *et al.* (2022). Abortion patient experiences with protestors while accessing care in Mississippi** | |
| Finding | Several participants commented that physical distance from protestors would have provided them with the privacy they desired. (U) |
| Illustration | “I had come there to do one thing, and they’re in my business. That’s something that you want to do and to be discreet about, and not to deal with in the street.” “If it [city ordinance] would have took place at the time, I would’ve been more at ease. You don’t have to worry about all that, [people] get close to you. Because then, it was horrible. Just a nightmare.” ^(p.891)^ |
| Finding | Most participants said that they had to find street parking for at least one of their visits, and (as noted above) they frequently reported that protestors would follow patients’ cars down the road and then approach them as they started walking toward the clinic. (U) |
| Illustration | “when I looked in the mirror, he [male protestor] was behind the car… The protestors were standing out there, and they would come be in your face and just try to talk to you.” ^(p.890)^  “I would’ve not been scared to drive by because they were really close. They were way less than 15 feet, right? … The way they were positioned, it was right by your car.” ^(p.891)^ |
| Finding | Many participants described being ‘bombarded,’ ‘bum rushed’ and feeling overtaken by protestors, who were standing in the road and on the sidewalk leading to the parking lot as patients turned on to the street where the clinic is located. (U) |
| Illustration | “I was so anxious, it was harder for me to breathe. I had my inhaler with me, but it wasn't helping. It took him [boyfriend], even then, to cover my ears to get me out." ^(p.889)^  “‘Oh my gosh. I think I want to find a different clinic.’ … I was so scared to drive my car. Even being in my car and they're outside of my car, I still felt like I was being attacked.” “[there’s] not that many options here [for abortion].” ^(p.889)^ |
| Finding | They overwhelmingly described protesters as “persistent,” “pushy and determined,” and “aggressive” as they made their way to and from the clinic entrance. (U) |
| Illustration | “The women, they try to talk to you, I guess, in a calm way in a maternal voice. The men [are] a little more aggressive. Just in the way they’re talking and … if you say you don’t want the pamphlet, they’re just like ‘Here, take it. Just look at it.” ^(p.889)^  “They don’t back of, even after numerous of times of being told to back of.” A Black 28-year-old echoed this saying, “You’re just like ‘If I tell you I don’t want the pamphlet, just respect that I don’t want the pamphlet. Don’t try to make me take the pamphlet.” ^(p.889)^ |
| Finding | Participants reported feeling unsafe in the presence of protestors, particularly when protestors surrounded the car and they could hear protestors yelling even though they were inside the vehicle. (U) |
| Illustration | “how aggressive they’re gonna get.” ^(p.890)^ |
| Finding | Some participants were also bothered to see children in the crowd of protestors. (U) |
| Illustration | "Found it unsettling to hear them repeat phrases like “Mommy, I love you, don’t kill me” and “Please don’t kill our friends.” Hearing protestors’ children ask, “You would kill me?”" ^(p.889)^ |
| Finding | Found their encounters with protestors more emotionally difficult. (U) |
| Illustration | ”when they arrived at the initial consultation visit. This participant went on to say, “It made me kind of feel sad because I pretty much have just—I asked for forgiveness, and for that to be thrown back in my face made me feel sad about the whole situation, but neither one of them are going to help me take care of a child for the rest of my life, so I have to do what’s best for me.” ^(p.890)^ |
| Finding | Credited clinic escorts and security with preventing more involved encounters with protestors. (U) |
| Illustration | “If it was not for the security they had really blocking you and walking you inside, the protestors will be bombarding you.” ^(p. 890)^ |
| Finding | Clinic escorts also played music and covered the fence around the clinic perimeter with a dark tarp to further shield patients from hearing and seeing protestors […] participants found these strategies to be helpful. (U) |
| Illustration | “I mean, they’re saying things to you, but, like I said, they have the music out there as well, and the music is toning it out, so you really don’t hear their exact words. You can’t make [them] out. That was very helpful.” ^(p.891)^  "Several others described wearing headphones or overing their ears as they made their way to the clinic or "turning my music up [in the car] until they [escorts] let me go inside” as additional strategies to block out protestors’ comments." ^(p.891)^ |
| Finding | Most protestors’ behaviors were not acceptable. (C) |
| Illustration | “We don’t need nobody trying to down-grade us, belittle us, or try to make us feel some type of way because we’re making a decision that works better for us at that moment.” ^(p.891)^ |
| **Study: Cashman *et al*. (2021). Women’s experiences of accessing a medical termination of pregnancy through a Queensland regional sexual health service: a qualitative study** | |
| Finding | Women’s experiences of obtaining an ultrasound were varied and depended on the individual provider’s assumptions about the pregnancy and scan. (U) |
| Illustration | "He didn’t ask anything, he just assumed [he said] ‘so baby’s this long, and you’re this far ahead, do you want to see do you want some photos?’ Surely the first thing you should have said, is do you want to see... I came out in an absolute ball of tears.’  "I think because they saw on the ultrasound form that it was pretermination. It felt a bit awkward, I did ask to see the ultrasound, but they said they’d prefer not to, so it was a bit – I never got to see it which I know a lot of people would prefer, but, for me, I wanted to." ^(p.E)^  "Everybody was lovely, they asked if I needed the screen on or off. Which was very sensitive, I thought that was lovely.’"^(p.E)^ |
| Finding | Reported a non-judgemental approach by both clinical and non-clinical staff at CSHS. (U) |
| Illustration | "‘it’s very easy to go there...they don’t judge you, [they’re] really helpful. I think they’re very close to women... The fact they are not judgemental I think has been the most positive thing in here, in my experience." ^(p.E)^ |
| Finding | One woman decided to go directly to CSHS after a previous experience with a local GP. (U) |
| Illustration | "I was actually marched out of a GP’s office one time who wouldn’t see me because he didn’t, he didn’t believe in that. So, I got ushered out into the waiting room... you feel a bit mortified, you know, it’s difficult enough as it is without being scolded for it." ^(p.E)^ |
| Finding | Conflicted thoughts on having an abortion while acknowledging that proceeding was the best choice for them. (U) |
| Illustration | "you go there already you think with some guilt, and you’re blaming yourself."  "...you know, I was against it [abortion] myself... I was thinking ‘Oh wow, shit, what are these people going to think of me?” ^(p.E)^ |
| Finding | The appointment for the MToP procedure was overall a positive, personal experience. (U) |
| Illustration | “She [the doctor] just very slowly and delicately went through what would happen, the medication,  things like that. And then I did have a moment where I sort of ended up bursting into tears...and  she just sort of let me have a cry and she just let me talk. It was just like, no, we’re going to stop and we’re going to pause... I didn’t feel rushed.” ^(p.E)^ |
| **Study: Chandrasekaran *et al.* (2023). The role of community and culture in abortion perceptions, decisions, and experiences among Asian Americans** | |
| Finding | Many participants also explained that sex before marriage was unacceptable in their family and  community and often equated the stigma of getting pregnant out of wedlock with the stigma of having an abortion. |
| Illustration | “you know, anything that deals with sex, to be honest, it’s very, very like hush-hush, taboo. . . . a girl gets pregnant outside of marriage it always is—like the girl is always the one who takes the blame. She’s the one who is shamed. And it’s just a huge stigma. And so I know that that is, even today, how Korean culture views pregnancy or, you know, abortion and sex, and I think a lot of those kind of beliefs still—it’s still around, even in Korean-American cultures” ^(p.06)^ |
| Finding | Participants often felt internal stigma and worried that others would view them as irresponsible. |
| Illustration | “It’s just like I feel like super embarrassing that I had an unintended pregnancy with someone that I didn’t want to create like a family with. I thought it was just very shameful. And I just—it was just really embarrassing, and so I hated myself because I was like I already—I had one, right? And then it was like I got myself into the same situation again and it could have been prevented if I was being smart about it, but like—yeah. It was just really embarrassing for me, for more of like having an unintended pregnancy than actually having the abortion I think.” ^(p.06)^ |
| Finding | Other reasons participants chose not to tell their family about their abortion included: fear of judgement, stigma related to having sex outside of marriage and getting pregnant, as well as stigma surrounding abortion. |
| Illustration | “But the community views about having—like getting pregnant while not married I think is what drove- that had more impact, I think. I was more concerned about what my family would think, especially my grandparents in India. It would have been awful. I just feel like it would not have gone well if they ever found out about that. So I think more of that kind of perspective of like getting pregnant like that, I wasn’t really thinking about views about abortion, I wasn’t thinking about it like that.” ^(p.06)^ |
| Finding | Some respondents shared feeling stigmatized by healthcare professionals when seeking an abortion, for getting pregnant, choosing to abort, and/or having had prior abortions. |
| Illustration | “I wish that when I first went to the clinic I wasn’t so judged by that doctor and like basically shamed by him. That was—it was already at the time shameful enough that I was requesting for abortion pills, so the fact that he was just double shaming me by, you know, giving me dirty looks and then saying like we can’t help you here was really disappointing and just made the process like even more stressful.” ^(p.07)^ |
| Finding | One respondent described being stigmatized by a provider who, like this respondent, was also South Asian. |
| Illustration | “It would have been really nice to have someone from the same background as me, but I say that with caution because my current OB/GYN is also South Asian and it’s also like a weird dynamic as well with that. . . I also feel like I’ve received judgment from her as well, because I said on my chart that I’ve had two pregnancies and I just feel like she judged me for that. . . at times, I felt like being talked down to, like kind of like saying, oh, you should already know this type of thing. . . I feel like as my doctor, you should be helping me learn to make healthy decisions instead of making me feel stupid that I wasn’t aware or whatever.” ^(p.08)^ |
| **Study: Chor *et al.* (2019)*.* Factors shaping women’s pre-abortion communication with members of their social network** | |
| Finding | Some women perceived that people writ large would judge them negatively about their choice to have an abortion. (U) |
| Illustration | “Some people would like be cruel, and like bring it up in the past, I mean bring it up in the future, and they’ll throw it at you and say, oh you know I thought you did it too. . . They might throw it in your face.” ^(p.4)^  “Like people that knows you or think they have the right opinion of you, they want to judge you. . . So I don’t need no one there to be judging me, or acting like they there to help when really they looking at something from a whole different point of view.” ^(p.4)^ |
| Finding | One such participant felt that she would have disappointed her family if they knew she was having an abortion. (U) |
| Illustration | “Uh, yeah my family, like my grandfather, and some, people that know I had abortions before, I didn’t want to find out because I didn’t want them to like judge me and stuff.” ^(p. 4)^ |
| Finding | Concerned about the opinions of others in her community. (U) |
| Illustration | “Just the whole abortion thing is - it’s a touchy subject. It’s embarrassing… First being a Christian, I’m still battling some things like is God mad at me? Like is people gonna be mad at me if they found out? I keep a lot of things to myself anyway, so I knew I wasn’t going to share this.” ^(p. 4)^ |
| Finding | Avoided discussing her difficult decision with her counselor due to concerns about her counselor’s religious convictions. (U) |
| Illustration | “Like my counselor that I had over the past two years like, been there for me through a lot of stuff, I didn’t even tell her what was going on… She wouldn’t have thought nothing bad of me, she just would have saw her religion in the situation and [inaudible], I just didn’t want to hear nothing about that.” ^(p. 4)^ |
| Finding | Concerns about being judged, women also expressed a desire to maintain privacy. (U) |
| Illustration | “I mean, there’s too much talking outside of my family. Like, no friends I could trust. At the end of the day, everyone talks, you know. So I just kept it in the family. I wouldn’t go outside my family and tell a friend, because when you tell a friend and your friend tells a friend and another friend tell a friend, tell a friend, tell a friend, and then the whole world knows.” ^(p. 4-5)^  “No, I didn’t feel comfortable telling anybody about it, so I was just like I’m just gonna keep this all to myself. I don’t want anybody to know at all.” ^(p. 5)^ |
| Finding | people’s reactions to hearing about a woman’s decision to have an abortion ranged from positive/supportive. (U) |
| Illustration | “. . . I thought she [a friend] will probably understand like my situation so you know, I dunno, she made me feel better about it. Even though you know it’s not something to be happy about, she made me feel a little better.” ^(p. 6)^  "Yeah, I talked to, um, my mom. You know my mom and dad are a great support system in my life, so… with them like growing old right now they want some grandkids. So I had to tell them, like, “Yea, you want grandkids, but I’m going to be stuck with the baby the majority of the time, you know, not you guys.” . . . I told them, “I’m just not ready right now. It’s not a good time.” . . . Yeah, my mom came with me. You know, my dad came to pick me up, my brothers was in the car. Like I say, my family, they’re behind me 100%, you know, they have my back. So they don’t make me feel like, oh I made a bad decision or nothing, they just want me to be happy, and OK with what I did." ^(p. 6)^ |
| Finding | Some who disclosed their decision to family or friends, however, received upsetting, negative responses. (U) |
| Illustration | "I mean I had some people say like I’m going to go to hell, and all this, but like, why you judge me?" ^(p.6)^  "After I paid I was sitting there waiting for them to come get me, I talked to my mom on the phone, and that’s the first thing that came out of her mouth. She was like, “You can die like that” and “You messing your body up.” And it was just stuff that- negative stuff- and bad luck [inaudible]. After her conversation I really didn’t want to hear no more…  I: And how did you feel about having to tell her and feel about her reaction?  R: I just, I was angry, it made me want to, me at the time I didn’t care no more, just wanted to get it over with, that’s how I felt, and then she’s shouting at me and doubting me, and [inaudible] going through my head. Like I was just ready to get it over with." ^(p.7)^  “Yes, my grandma told me, that you know, that’s not good. I think those things are cancerous. You know, you get different opinions about it but it’s something you gotta do.” ^(p.7)^ |
| **Study: Dickey *et al.* (2022)*.* “They’re forcing people to have children that they can’t afford”: a qualitative study of social support and capital among individuals receiving an abortion in Georgia** | |
| Finding | Women described receiving some form of social support during their abortion. (U) |
| Illustration | "“were helping me in my decision actually to have an abortion,” ^(p. 4)^  “called after, just to make sure I was okay” ^(p. 5)^  “whatever I decided to do, [they] would be there.” ^(p. 5)^ |
| Finding | Most women described abortion stigma – and their social network not being “supportive of [my decision]” (Destiny, 20, Black) – as a barrier to support and access to abortion services. (C) |
| Illustration | “supportive of [my decision]” ^(p. 5)^ |
| Finding | Many women had reduced social support because they were unable to tell individuals in their network, or their entire network, about their abortion due to concerns of stigmatization. (U) |
| Illustration | “everybody don’t believe in abortion, so you can’t go to everybody and just ask, ‘can you help me with this?’” ^(p. 5)^  “of course you’ll be judged [for getting an abortion].” ^(p. 5)^  "having a “good support system” in general, but she “couldn’t even trust them” due to their beliefs about abortion and lamented, “I wanted to tell my mom … I wish I could have told my best friends … I wanted that comfort.”” ^(p. 5)^ |
| Finding | Describing abortion as being tabooer than any other health condition. (U) |
| Illustration | "Described abortion as “more taboo than any other health condition.”” ^(p.5)^ |
| Finding | Faced stigmatization when they disclosed intentions to terminate their pregnancy.(U) |
| Illustration | "her “[older brother] wasn’t willing to help, so [younger brother] stepped in and helped. [Older brother] didn’t agree with my decision, so he didn’t feel comfortable helping.”" ^(p. 5)^  “when I told them what I was gonna do, two of them, they didn’t want me to go forward.” ^(p. 5)^  "“didn’t tell [her] aunt and [her] mom until after the abortion was over” because they viewed it as “taking a life;” when she went to them for emotional support afterward, “they were sad … [mom] was mad.”" ^(p. 5)^ |
| **Study: Doran and Hornibrook (2016). Barriers around access to abortion experienced by rural women in New South Wales, Australia** | |
| Finding | External stigma was exacerbated by protestors. (C) |
| Illustration | “While strangers are still waving placards, telling me what to do with my body there is stigma. Well, can you buy RU486 [for medical abortion]? Can you get it from your GP? I think there’s lots of stigma." ^(p.5)^ |
| Finding | Consequences of stigma and the lack of respect or women to make reproductive decisions concerning their own bodies. (U) |
| Illustration | "Out bush – there is still a lot of stigma about getting information in the first place and certainly something that is not talked about … and I reckon there’s a higher rate of teenagers having births because of the stigma." ^(p.5)^  "Stigma is linked to the expectation of society for a woman to be loving and nurturing and to think of others. When a woman chooses to have an abortion, she is making that decision for herself. I think if women were really respected as free thinking individuals, people who are allowed to make decisions for themselves, then that stigma wouldn’t be as strong." ^(p.5)^ |
| Finding | Internalised stigma was linked to feelings of shame and secrecy. (U) |
| Illustration | "[...] comment that abortion is 'still a taboo topic’’" ^(p.5)^  "it’s not something you explain to people, you just kind of have this mysterious absence" ^(p. 5)^ |
| Finding | When they shared their experience it became evident how many other women had not only had an abortion but had kept it secret. (U) |
| Illustration | "It was an eye opener when everyone I had disclosed to had also told me they’ve had at least one, so why is it so difficult? It’s not a general topic of conversation: it’s all a bit hush hush though everybody does it, so to speak." ^(p. 5)^ |
| **Study: Foster *et al*. (2020). ‘‘I didn’t doubt my choice, but I felt bad”: A qualitative exploration of Canadian abortion patients’ experiences with protesters** | |
| Finding | Many participants found encounters with protesters to be emotionally upsetting and stigmatizing. (U) |
| Illustration | "I was already nervous. I guess there’s just literally people that do that [protest] and it makes me feel bad. Like, I didn’t doubt my choice but I felt bad." ^(p.311)^  ‘‘They were just out there, it was outside the hospital gates but I saw them on my way there and I was just like oh my god. I felt threatened and I felt guilty too.” ^(p.311)^  "having an abortion was ‘‘so bad” but the protesters made her feel ‘‘like it was way worse than I ever could have imagined.” She described her experience as emotionally upsetting, ‘‘hearing them in the clinic...yelling things outside [made me] so uncomfortable." ^(p.311)^  ‘‘If anything it made me feel a little bit more ashamed, but I already sort of felt that way anyways”. ^(p.311)^  ^“^That was the hardest decision I’ve ever had to make, and they are making that harder.” ^(p.311)^ |
| **Study: Frohwirth *et al.* (2018)*.* Managing religion and morality within the abortion experience: qualitative interviews with women obtaining abortions in the U. S** | |
| Finding | Religion Within the Experience of Internalized Abortion Stigma . (U) |
| Illustration | "at church, yeah, we are really against, it’s more… once I talk about it, it makes me feel bad [crying], like […] having the baby but giving to adopt, not killing it. So it’s something really hard for me right now." ^(p.7)^  "So you know, it’s just something that haunts you and you—I mean, everybody has different beliefs, some people are Atheist, some people are Protestant, Catholic, whatever. I mean, me personally I am spiritual and so I feel like if I go through with this again, how is God going to punish me, later on? You know, if—when we are ready and say we have our finances in place, we finally have our own house, everything is perfect, right?" ^(p. 7)^  "It’s really hard because I grew up Catholic, but probably after today, I won’t be." ^(p. 7)^  "I am just worried that, you know, like, my fiance and I wanted to start taking our kids to church every week, and I felt like, well am I going to be able to go now, and not feel guilty or wrong to be there, and, you know, will it really affect, like, what will happen when I die later on?" ^(p. 7)^  "I went to Catholic school and basically [they say that abortion is] just like the most horrible thing you could ever do, and you are taking away God’s life, and blah-blah-blah, and like, just exaggerated to the point of like craziness. So it didn’t really affect me, but those things always be in your head, even though you say, “I don’t believe in that,” but that little voice keeps coming on, like, “You are killing your kid.” ^(p. 7)^  "…I would cry that, like, what have I done? I know this is not a morally good thing. I am not religious or anything, so it doesn’t affect me, like, religiously…" ^(p. 8)^  "Abortion, I always just thought was just not …just not right. So I never pictured myself being here. It made it that much harder to make this decision to be here today, because I never believed in this. This like, being here goes against everything that I’ve ever believed in, like I didn’t grow up religious. I don’t go to church. I don’t, I don’t pray. I don’t read anything. I don’t, I just … but I don’t believe that this should be okay… "^(p. 8)^  "…but it’s just, like, in certain circumstances, a child should not be brought into the world I feel. I’m a Christian, so it completely goes against, like, what I believe, but, you know, that’s just what I think, and I think that certain religions shouldn’t hold you back of what you—how you feel. I don’t think that’s right." ^(p. 10 - 11)^ |
| Finding | Felt stigma was entwined with religion for our respondents; women specifically feared religiously based condemnation if they revealed that they had had abortions. (U) |
| Illustration | "I guess my parents are pretty … they are Catholic, so, I mean, of course, if I was to tell them, they would absolutely not allow it. " ^(p.8)^  "…my family is very Catholic so I am, like, terrified to even think about telling them."  "It’s kind of wrong because I never been, like, raised to do this. Like, [if] my family even knew I was doing this, they’d probably be upset with me ‘cause we don’t— they just don’t believe in it. So it’s all about your religion as well." ^(p. 8)^ |
| Finding | Fear of judgment extended to the larger community. (U) |
| Illustration | "The people in the town are all older, so it’s a very conservative town, so for people to know that I went out of town to have an abortion would just be like, ungodly to them, because it’s a small town and “our kids don’t do that,” you know, that kind of thing." ^(p. 8)^ |
| Finding | Experiences of enacted stigma were also explicitly linked with religion. (U) |
| Illustration | "…my father and my stepmother—I had a lot of family in [city], and they stopped speaking to me. My stepmother is a “Born Again,” I think is what she calls herself, and so I was immediately a murderer and going to hell and embarrassing the family." ^(p. 9)^  “…I have talked to my mom about it and I told her I wanted to have an abortion. She hates me. She doesn’t talk to me. She kicked me out of the house. I was staying with her. Right now I’m staying here and there, wherever I can, and with my son, and she says that I’m a bad mom, that I’m the worst person ever, that if I’m having an abortion I don’t love my son either, that God should have never gave me the gift to be a mom. That’s just so hard, because my whole family hates me. They don’t understand what it’s like to be in my shoes or why I don’t want to be pregnant.” ^(p. 9)^ |
| Finding | Eperienced enacted stigma […] in their communities and physical environments as well. (U) |
| Illustration | "I: In your opinion, how did people in your community view abortion?  R: … where I live it’s mainly Hispanics, and Hispanics are very religious, and so it’s like half and half, so you really don’t know. It’s kind of weird, like, if you could go down the street and you could see a billboard that says you know “Killing a baby from God is a sin or something” […]so it’s pretty much like the environment you are in, that is a big thing." ^(p.9)^ |
| Finding | Strategies for Managing Religiously Based Abortion Stigma: Personal Exceptionalism. (U) |
| Illustration | "I: How did the father [feel], the man involved with the pregnancy, what’s his reaction?  R: He wasn’t too happy that we have to have an abortion, but he knew that it has to be done, so we can be able to get our son [back from state custody], because he [the father] is a Roman Catholic, but his dad’s a Jehovah’s Witness, and I am a Christian, and his family doesn’t believe in it, I don’t believe, and my family don’t believe in it. But when I went and told them that I had to have it done, they kind of like went off on me, but then after I sat down and explained to them why I had to have it done, then they kind of realized, you know, okay it’s just this one time, right." ^(p. 10)^  “…but it’s just, like, in certain circumstances, a child should not be brought into the world I feel. I’m a Christian, so it completely goes against, like, what I believe, but, you know, that’s just what I think, and I think that certain religions shouldn’t hold you back of what you—how you feel. I don’t think that’s right.” ^(p. 10-11)^ |
| Finding | Strategies for Managing Religiously Based Abortion Stigma: Rejecting Religion. (U) |
| Illustration | “I: How does having an abortion fit in with your personal beliefs?  R: Actually, I don’t really have a special religious preference, so I believe it’s everybody’s decision, […] that is just how I feel. I am very open-minded about stuff, so, and then, I don’t have a religious preference, so just go on my own, really.” ^(p. 10)^  “In my church they don’t like it [abortion], but that’s the church rule. Everybody in church has their church rules, and then their own set of rules. It doesn’t go against my personal morals because I am a “whatever” type of person—whatever you want to do, whatever you feel comfortable doing….”^(p. 10)^ |
| Finding | Strategies for Managing Religiousl´y Based Abortion Stigma: Revising Beliefs.  (U) |
| Illustration | "I went to a Catholic school my entire life up until college so like, this decision also is, like, I have learned since I was in first grade that this is wrong. But I have friends that have gone through it so, and now that I am in college, it’s like, you know what? I have an open mind." ^(p.11)^  "Well, I was raised as a Catholic so it was always “abortion is wrong, gay marriage is wrong,” and now that I have …I actually have gay relatives and I have done … like in high school I did a little bit of research about like the Plan B pill, and you know I’ve kind of moved a little bit away from the Catholicism. Just because it’s so strict […] I went to like Catholic schools, Catholic churches, like my whole life, and so I was just done with it, and then I kind of got my own opinions, and I’m really glad I got my own opinions." ^(p. 11)^ |
| Finding | Strategies for Managing Religiously Based Abortion Stigma: Challenging a Stigmatized Status. (U) |
| Illustration | "… so they [people at church] think God wants you to be this, but I honestly think God knows we makes mistakes. […] I don’t think he expects us to be perfect… " ^(p.12)^  "Everybody that I’ve been around, they very big in church, so it’s like a negative and a positive side. They don’t try to judge me, but at the end of the day, they do want you to keep it. So it’s like, it’s all about the religion, basically. I believe in the same religion, but I feel like we all make mistakes and, you know, we ask for forgiveness, we move on. That’s how you do it. There’s nothing else you can do about it. We all do something we should not do, every once in a while at least. I know I do, shoot. So, yeah. Nobody’s perfect." ^(p. 12)^  "I mean, I go to church sometimes, but I am not [religious]. For some reason, I just don’t think they, like those people outside [protesters] were saying, like “Mother Mary” and all the stuff, and for some reason, the God that I believe in would not say that this is not okay. I am sure that he would like me to have it [the baby], but I don’t think that you are going to get punished because of something you thought you needed to do." ^(p. 12)^ |
| Finding | Pointing to the fact that they had had previous abortions and had not been punished by God. (U) |
| Illustration | "I am a Catholic, Catholic people don’t believe, supposedly, they don’t believe in abortions. […] And, you know, they believe that if you have an abortion you are going to hell. I don’t believe it. I mean, women way before my grandma’s been having abortion[s], you know. See, I kind of see it as if God was to punish us, like if you rob a bank, God is going to punish you by going to jail because you robbed a bank. So, I mean, I have had an abortion [referring to first abortion], but God didn’t punish me. Actually, he kind of blessed me, you know, because I ended up, you know, going to school […] I don’t believe that God punishes you for an abortion." ^(p. 12)^ |
| Finding | The only true judge of their behavior was God. (U) |
| Illustration | "God’s the only one that could judge me. […] To me, I have always said nobody can judge you, nobody is in your footsteps, nobody is there, nobody can judge you. […] I mean anybody can judge me, my parents, it doesn’t matter to me; the only person who can judge me is God." ^(p. 12)^ |
| **Study: Gelman *et al.* (2017)*.* Abortion Stigma Among Low-Income Women Obtaining Abortions in Western Pennsylvania: A Qualitative Assessment** | |
| Finding | Negative views in partners with whom they did not discuss the abortion. (U) |
| Illustration | A 41- year-old woman who had had an abortion explained why she had not discussed the decision with her partner: “He don’t believe in [abortion]. There’s no way he would’ve went for that.” ^(p. 5)^ |
| Finding | Family members had antiabortion views. (U) |
| Illustration | For example, a 22- year-old pregnant participant said, “I didn’t tell my mom because my mom wouldn’t even let me do it.” Other family members were also mentioned. A 41-year-old who had had an abortion said, “As I got further along, … two of my cousins was trying to get me to keep [the pregnancy].” ^(p. 5-6)^ |
| Finding | Perceived their broader social networks to be disapproving of abortion. (U) |
| Illustration | “if members of her community discovered that she had had an abortion, “I would probably be tarred, feathered and run out of town with pitchforks.” Several women indicated that others were “mad” at them, including a 22-year-old pregnant participant, who explained, “Everybody’s mad at me.” Direct exposure to “prolife” and “antiabortion” propaganda was infrequently mentioned as participants spoke about abortion views in their wider communities. ^(p. 6)^ |
| Finding | Participants themselves were the most common source of disapproving attitudes toward abortion. (U) |
| Illustration | “A 23-year-old pregnant woman explained, “I never really believed in [abortion].” A 24-year-old pregnant participant shared this view, stating, “I don’t believe in abortions at all. … And now I have to do something that I really don’t believe in.” Similarly, a 38-year-old who had had an abortion reported, “I was [against abortion] myself until I ended up having to have one.” Another third of participants were ambivalent about abortion. For example, a 33-year-old woman who had had an abortion said, “I’ve never been proabortion, you know. I’ve never been that kind of person. Neither am I antiabortion.” ^(p. 6)^ |
| Finding | Abortion was commonly framed as an immoral act and frequently depicted as “murder.” (social network) (U). |
| Illustration | “They look down on it because you’re killing a life.” A pregnant 19-year-old described how her fiancé’s mother had reacted to her decision to pursue an abortion: “She said … ‘You can’t kill my grandbaby. That wouldn’t be right.’” She then explained her own view prior to this pregnancy: “I was so against abortion … ’cause it’s wrong, it’s wrong. Before I got pregnant, I thought of it as murder.” Similarly, 22-year-old pregnant participant said, “Before, it was like … ‘Why is she killing a kid that’s innocent?’” ^(p. 6)^ |
| Finding | Abortion specifically violated their own or their family members’ religious belief. (U) |
| Illustration | “even knowing the health problems.” She explained that because of religious beliefs, her grandmother had kept “hoping that they could help and do something, because she did not want me to have an abortion.” Similarly, a 23-year-old participant who had had an abortion said, “My mom pretty much told me it’s a sin. She’s a Christian, and she goes to church.” ^(p. 6-7)^ |
| Finding | Abortion represented a rejection of motherhood. (U) |
| Illustration | “If you are pregnant, you have the baby. That’s it.” This description was similar to the one a 21-year-old pregnant woman gave of views in her social network: “Why don’t you just accept it and be that mother that you already are?” ^(p. 6)^  A 22-year-old participant who had had an abortion explained that her friends, who are “all young moms,” were “content having a baby so young [and] never thought about having an abortion or anything.” ^(p. 6)^  “All my family got kids, like seven to eight. They just [think], ‘If we did it, then you can do it.’” ^(p. 6-7)^ |
| Finding | Abortion was perceived as a rare and thus potentially deviant event. (U) |
| Illustration | “I thought [abortion] was that kind of like, every once in a while kind of thing. But when I was looking into it, … I was pretty wowed, ’cause that’s a lot of people.” ^(p. 7)^  “Before, I didn’t think it was so common, but when I was at the clinic, there were a decent number of other people. … I just thought it would be me and maybe like one or two other people.” ^(p. 7)^ |
| Finding | Misperceptions about abortion’s jeopardizing their future fertility. (U) |
| Illustration | “I wouldn’t do it again just for the simple fact that they say it messes up a woman’s insides. If I ever do decide that I want to have a baby, I probably already damaged everything in there.” ^(p. 8)^  “she thought it was unsafe to have multiple abortions and that women who do so are “not able to have kids [and have] complications with their pregnancies.” ^(p. 8)^  “[When] you’ve had so many abortions, it messes up your body.” ^(p. 8)^ |
| Finding | Distinguish the circumstances of their abortions from those of other women’s abortions. (U) |
| Illustration | ““an accident or whatever.” She went on to say, “This [abortion] was my ‘oops.’ … When you have two, three, four, I don’t think that’s right. I really don’t.” Similarly, several participants classified their own reasons for abortion as valid or acceptable, while criticizing women who use “abortion as birth control” or have abortions “because they feel like it.” A 33-year-old who had had an abortion, and who reported that her pregnancy had been the result of a condom’s breaking, distanced herself from women whose abortions were attributable to “just outright carelessness.” She continued, “I’m not proabortion. But in my case, I tried to avoid pregnancy, and it happened anyway.” ^(p. 8)^ |
| Finding | Not telling someone in their social circle about their abortion specifically to avoid arguments,  social isolation or damage to their reputation. (U) |
| Illustration | “I don’t really know how [people in my circle] feel about that issue, and I don’t really want to cause any unnecessary hard feelings or something like that.” ^(p. 8)^  A 41-year-old participant explained why she did not tell her partner about her abortion: “’Cause I didn’t want him to try to give me any kind of inclination that I should keep it.” ^(p. 8-9)^ |
| Finding | Valid reason they pursued one. (U) |
| Illustration | “I don’t really agree with abortion, because I personally do feel somewhat like it’s slight murder. But on the other hand, you have people who, you know, it might come in handy for. … You have people like me. I just had a baby, and I just can’t do another one right now.” ^(p. 8)^ |
| Finding | Inconsistency between their antiabortion views and their behavior, several women described negative emotions. (U) |
| Illustration | A 24-year-old pregnant woman, who said that she does not believe in abortion, explained, “I’m  really upset at myself. … I’m mad at myself.” A 38-year-old described herself as “very against abortion” and explained that she feels “sad, regret, guilt” about her recent abortion. A 41-year-old participant, who said that she had “really regretted” her first abortion many years ago, explained how she viewed her second abortion, which she had at a later gestational age: “I was further gone than I wanted. I felt like I was killing a kid this time.” ^(p. 9)^ |
| Finding | Religious beliefs shape her perspective of her recent abortion as shameful. (U) |
| Illustration | A 33-year-old participant shared how her religious beliefs shape her perspective of her recent  abortion as shameful: “Because I have a very strong faith in God, I am ashamed. … And that’s something that I have to deal with forever. That’s my biggest fear and regret.” ^(p. 9)^ |
| **Study: Grindlay *et al.* (2017)*.* Abortion knowledge and experiences among U.S. servicewomen: a qualitative study** | |
| Finding | Concerns about stigma and the impact on their career should anyone in the military find out about their pregnancy or abortion. (U) |
| Illustration | “I did not tell my chain of command at all. Part of that was because I’m only one of the very few females that works within my department…There’s a lot of like negative stigma that kind of goes along with that…I was recently promoted,…and this was right before my promotion.” ^(p.248)^  “didn’t even consider” going to a military treatment facility by saying, “It has been my experience with friends that they’ve had negative repercussions…for seeking help [for an abortion] through the military.” She further recounted, “I have seen people not necessarily get kicked out, but I have seen their careers affected by [abortion].” In a similar vein, an enlisted army servicewoman reported, “I didn’t want the army to know until after [the abortion] was over and done with, because I didn’t know what they would’ve done.” ^(p.248)^ |
| **Study: Hatcher *et al.* (2018)*.* If, when, and how to discuss available abortion services in the primary care setting** | |
| Finding | Participants emphasized the importance of a non-judgmental approach. (U) |
| Illustration | “[the provider] is not trying to impose anything on the patient, but that it is her obligation as a physician to provide all care.” ^(p. 10)^ |
| Finding | Personal experience with an unplanned pregnancy during which she felt judged. (U) |
| Illustration | “I didn't go back [to the provider]. I felt personally attacked - who is she to tell me what choices I should make in my life? …she thought, don't have kids too young…she was kind of communicating her personal opinion or what she would have done in my situation rather than asking me what I wanted.” ^(p. 11)^ |
| Finding | Emphasized the importance of using kind language and compassion to address the conversation in a sensitive manner. (U) |
| Illustration | “People just aren't comfortable enough with the subject, and so it needs to be handled very delicately… So just trying to treat it like it is serious and important, not to treat it like a joke, I guess, because I feel sometimes that's how that's taken, to like lighten the awkwardness.” (age group 19-24 years) One patient summarized the importance of being sensitive by stating, “regardless of someone's choice or their beliefs, just tread lightly.” ^(p. 10)^ |
| **Study: Heller *et al.* (2016). Barriers to accessing termination of pregnancy in a remote and rural setting: a qualitative study** | |
| Finding | Experiencing stress and anxiety relating to the cultural and temporal factors. (U) |
| Illustration | "I think it’s really quite a shame. Like because ...there’s going to be a lot of people who are going to have to come up with ridiculous scenarios to tell their parents, to go away and that. And then like what if there was like people like in the school? Because they’re going to be like “oh, I’m going to Inverness for a few days” and like where are you going to get the money for that?" ^(p.1689)^ |
| **Study: Hermannsdóttir (2022). Maneuvering in silence: abortion narratives and reproductive life histories from the Faroe Islands** | |
| Finding | Valued female citizen has children at the right time and in the right way. (U) |
| Illustration | "And it’s the same with abortion. The fact that you cannot talk about it. Like my friend – I don’t think she has any psychological problems due to it [abortion] – but just the fact that she can’t say “Yes, oh my God, it was good that I had that abortion.” Or just that she can’t talk about it, something that’s normal and something that is okay. Without feeling that she’s done something horribly wrong and that she is selfish." ^(p.817)^ |
| Finding | Spoke of emotions of loneliness and othering. (U) |
| Illustration | "I didn’t really talk to anyone about it, and I was just so, I literally thought I was going to be the first Faroese to have an abortion, because I’d never heard of anyone else." ^(p.817)^  "I’ve been so afraid of it [speaking about abortion and supporting pro-choice activism]. Because what if I supported it, then people would probably think I’d had an abortion, and what if they knew I’d had an abortion, what would they think? (. . .) There are many enemies out there that you must be ready for." ^(p.818)^ |
| **Study: Hoggart *et al.* (2017)*.* ‘Repeat abortion’, a phrase to be avoided? Qualitative insights into labelling and stigma** | |
| Finding | Women who had experienced more than one abortion there was a stronger sense of failure and self-blame. (U) |
| Illustration | “once is bad, twice is unforgivable … I just felt like a bit of a wrong ‘un’”. ^(p.28)^ |
| Finding | Would be punished later in her life for having two abortions. (U) |
| Illustration | "felt that she would be punished later in her life for having two abortions: “It’s just what happens, isn’t it? It’s just what ends up happening. You do something like that and then your punishment is basically that you can’t have kids or something goes wrong or miscarriage”" ^(p.28)^ |
| Finding | Recognition of the increased potential for shame, even those who resisted self-blame. (U) |
| Illustration | “Three times, that’s really, really bad … I can’t really blame myself because I’m on contraception and I’ve been using condoms now and again … I use the Cerazette® and I also took the morning-after pill with this one” ^(p.28)^ |
| **Study: Hoggart (2017). Internalised abortion stigma: Young women’s strategies of resistance and rejection** | |
| Finding | Talk generally of not ‘believing’ in abortion, as an explanation for why they felt they had done something wrong. (C) |
| Illustration | "Katie: I didn't believe in abortions.  Interviewer: That's what a lot of people say as well, so I wonder if you can try and explain to me why you don’t believe in abortions, or why you say you didn't believe in abortions?  Katie: Because, I don’t know, you’re getting rid of say like a part of you.  Interviewer: Your friends or the people you hung out with, what did they think about having abortions, do you know?  Katie: My mate [friend] is pregnant, I said to her “Are you having an abortion?” but she don’t believe in them so she’s going through with it. " ^(p.6)^ |
| Finding | Describing themselves as selfish. (U) |
| Illustration | "I suppose it’s a selfish decision on my part but also I didn’t want to bring a child into something where I couldn’t give them everything I wanted." ^(p.6)^ |
| Finding | Deciding to have an abortion in the interests of existing children, thereby drawing on gendered notions of ‘good mothering’ was one of the most strongly expressed justifications for an abortion. (U) |
| Illustration | "And I was like, “I’ve got a three month old baby, what is best for my three month old baby is not for me to be getting very ill, being pregnant again.” Lara described her decision to have an abortion as “completely the right decision” and said she had “no regrets at all”, despite describing herself as personally opposed to abortion, both before and after her own abortion." ^(p.7)^  "I’m already a single parent, I’m 22, my daughter is three and a half now, she’s doing brilliantly. I’ve got a nice home, I’m scraping by, like week on week it is a struggle to get us through. If I had another mouth to feed, it would mean that her quality of life would drop and I know it probably sounds really horrible because that was a baby as well but she’s here and now, and she’s more important than anything … And so I just thought really, it was for the best." ^(p.7)^ |
| Finding | Expresses strong moral opposition to abortion she is able to justify her own abortion by pointing to her extremely poor health. (U) |
| Illustration | "I’m angry at myself for putting myself in the position where I had to go against what I believe in, because I still believe that abortion is wrong. But, on the other hand, I know that, I think the thing that swayed it for me and the only reason I went through with it, I would have kept the baby if it wasn’t going to have killed me." ^(p.7)^ |
| Finding | Participants without children also drew upon notions about the ‘right time’, to become a mother. (U) |
| Illustration | "And I said to him, I don’t think it’s fair to the child right now because I don’t have anything to give anyone, like, emotionally. I don’t think that’s fair. I think a lot of – lots of people, you know, if they feel like they’ve got a hole in their heart or something, they try and fill that by having a child and making them happy, but I think you should be happy within yourself before you can offer a child anything." ^(p.7)^ |
| Finding | Concern was not having to struggle financially. (U) |
| Illustration | “I’d like to be able to give my children what they want and not struggle, I know it can be done, but I don’t want to struggle, I want my kids to just have anything they want. I don’t want them to be spoiled but I want them to have opportunities and have money really. So the reason why I did it was because I thought I need to get a job and make sure that I can have a child when I can support it.” ^(p.8)^ |
| Finding | Driver in decision-making was rejection of unplanned motherhood in the context of what they thought might not be a lasting relationship. (U) |
| Illustration | "I’ve always said to myself, I’m never going to have children until I’m well off or you know I’m in a relationship where I love that person and I know that I’m going to be with that person forever, because what’s the point of having a child and bringing it into a broken home." ^(p.8)^ |
| Finding | Unintended pregnancies that had occurred despite what was described as careful and responsible contraceptive use. (U) |
| Illustration | "If it wasn’t an accident and I’d not been using anything I wouldn’t have gone through with it but as I was trying to prevent myself from getting pregnant and it was obviously breakage of a condom, I think that’s why I did." ^(p.9)^  "“it’s a bad thing to do”. The use of contraception protected her from blame; but not from guilt: Maddie continued, “I feel like I made the right decision but sometimes I do feel a bit guilty”" ^(p.9)^ |
| Finding | No trouble discussing her decision-making with her family and friends. (U) |
| Illustration | "I mean everybody has said they’ll support me whichever decision I make, especially my parents and my partner’s parents. Obviously for them grandchildren would be amazing but they know it was the wrong time. They’d have been there no matter what decision we’ve made. Same as friends really, just helped me make my decision, made it a bit easier as opposed to making me feel bad about it, they’ve supported the fact that this wasn’t the right time for it" ^(p.9)^ |
| Finding | Women who had not been using contraception and became pregnant articulated blame as well as shame. (U) |
| Illustration | "I just felt [pause] like a bit of a slapper. You know? Like, it’s not difficult. Contraception is free. It’s not difficult to come by, and if you don’t want to go to the doctor’s there’s … I mean I know this area really well and there’s places all over the shop where you can access contraception. And you know, I just felt like a bit of a wrong ‘un, you know." ^(p.9)^ |
| Finding | Women who had experienced more than one unintended pregnancy and abortion, however, there was often a stronger voicing of self-blame, particularly for their sexual behaviour. (U) |
| Illustration | “once is bad twice is unforgivable” ^(p.9)^  “It’s just like one time is probably bad enough, but the second time it looks quite irresponsible” ^(p. 9)^ |
| Finding | Narrative thereby contains elements of a stigma narrative she has indirectly rejected internalised abortion stigma. (U) |
| Illustration | "I knew what I had to do. It was an unfortunate situation. I kind of just stayed on the surface, got it done then I don’t really look back. So, I mean, yeah, I did have moments before I actually got it done where I would lie in bed and I mean I couldn’t feel anything inside me, but I did have the moments of sadness where I thought there’s actually my child is in my stomach, you know, that’s amazing and I had them feelings but as I said I just didn’t go there because it would have really upset me. And I, it is always going to be a part of my life and something I had to go through but, life happens, you know." ^(p. 10)^ |
| **Study: Holten *et al.* (2021)*.* Permeability of abortion care in the Netherlands: a qualitative analysis of women’s experiences, health professional perspectives, and the internet resource of Women on Web** | |
| Finding | All protestors use a moralising discourse. (U) |
| Illustration | “When I got there, a man was standing across the street and he was screaming, […] he had a very big sign saying ‘Jesus is lord’ […] and he was yelling at the couple that came outside [the clinic] ‘You are murderers, you go to hell’ […] I was in shock.” ^(p.167)^ |
| Finding | Knowing there could be protestors was a barrier in accessing regular abortion care. (U) |
| Illustration | “I find it too big a step to go to a clinic myself. I have often heard that they are standing outside protesting and that is why I would prefer it if I could do it at home with my partner.” ^(p.167)^ |
| Finding | For migrants, […] keeping the abortion secret was often imperative and was motivation for seeking a self-managed abortion. (U) |
| Illustration | “Unfortunately I cannot talk to anyone about my pregnancy, especially my family. I come from a conservative and religious family where sex before marriage is considered a sin and it would be a huge disappointment for them. I don’t want to go to the clinic because there’s a big risk I might get caught by my parents.” ^(p.167)^ |
| Finding | Difficult to talk with family and friends because they were often afraid of their emotional or judgemental reactions. (U) |
| Illustration | “I deliberately did not want to talk to other people because I was too afraid that someone else’s opinion would influence me in a bad way. That I would feel guilty […] So I kept it to myself.” ^(p.167)^ |
| Finding | Most women were pleased with how they were treated by HPs in the abortion clinic. (U) |
| Illustration | “I had a doctor sitting across from me who was very understanding and very warm in her com-munication and then that was really only very pleasant for me. Because of that warmth and the  amount of information, I could immediately make a well-considered choice.” ^(p.168)^ |
| **Study: Holten *et al.* (2023)*.* The difficult process of autonomous choice: using I-poems to understand experiences of abortion-seekers in The Netherlands** | |
| Finding | Shame had to do with the stigma surrounding abortion, as the poem shows, wherein the abortion seeker felt that an unplanned pregnancy and subsequent abortion was something that only happened to stupid people. |
| Illustration | “Only stupid people  I actually felt even dumber  I felt even dumber  ohh  I need to cry  because I’m thinking about it now  I felt stupid  I already felt very stupid because  I knew too  I thought that  unintentionally getting pregnant  would only happen to stupid people  although  I don’t even know what stupid people are  I find few people stupid  but that image” ^(p.7)^ |
| Finding | But then, external factors such as the partner’s/family’s wishes, stigma, health system restraints, legal factors, missing information, and necessary support intruded on their decision-making pro-cess and complicated it. |
| Illustration | Deciding about my body  I felt very much under pressure  I asked my aunt to support me  and do the talking for me  I had a mother who was determining  you’re not going to keep it  not in my house  [my mother-in-law] started texting me  I was a murderer  I would go to hell  I would get uterine cancer  I felt that she was going to convince him  at first he had no problem with it at all  that I wanted an abortion  I did feel that he started to  change his mind  I felt there were three women who were  deciding about my body  L Holten et al. Sexual and Reproductive Health Matters 2023;31(1):1–17  9 didn’t listen to what I really wanted  I don’t regret it at all  afterwards I didn’t have any regret  that I had it removed  I didn’t want to be pregnant  I didn’t want a child either  not what I wanted for myself  but what I found very difficult is that  so much was determined  around me  what I should and shouldn’t do” ^(p.9)^ |
| **Study: Hutchens (2023)*.* “It Wasn’t Very Public-Clinicy”: Client Experiences at Faith-Based Pregnancy Centers** | |
| Finding | She spoke at length to the stigmatizing conversation she deemed “irrelevant”. |
| Illustration | “When I was talking to that nurse, the more questions she asked—I think you have religious views . . . she made me feel like a slutty person for having sex before marriage with someone that I wasn’t sure about being with. . . . I mean, I was there to get a pregnancy test and that’s it! . . . But she was like, “Well you maybe should consider not having sex until, you know.” And . . . that’s not why I’m here. Because if I want a speech about abstinence, I would go to the church or my mother.” ^(p.497)^ |
| **Study: Kavanaugh *et al.* (2019)*.* “It’s not something you talk about really”: Information barriers encountered by women who travel long distances for abortion care** | |
| Finding | The secrecy surrounding abortion in general, coupled with the inaccessibility of clear information and resources about where to obtain one, created an atmosphere of doubt around  Abortion. (U) |
| Illustration | "We just didn’t know exactly how any of this worked, what kind of place we were going to be coming to. Is it like a dungeon-like place or what the hell is this place? […] We were both worried about it being done in a clean place, that wasn’t like some place that they were just going to have some barbaric way of doing something." ^(p. 8)^ |
| Finding | The barriers of a lack of information, a lack of help from the healthcare community and the  perceived stigma of abortion care are, of course, related. (U) |
| Illustration | "Well I called my regular OB/GYN […] I am pretty sure I want to terminate […] Of course, they were like, “We don’t offer those services.” Okay, well can you refer me? My doctor was not able to. My next stop was [national chain of reproductive healthcare providers] […] They really could not direct me to anybody in the state of Texas. They said that the closest thing that they could recommend would be in Dallas or Fort Worth, and I know that that would probably involve, like I said, a lot of protestors and a lot of judgment that I didn’t really want to deal with […] so that was when I got on Google." ^(p. 8)^ |
| **Study: Kerestes *et al.* (2022)*.* Person-centered, high-quality care from a distance: a qualitative study of patient experiences of TelAbortion, a model for direct-to-patient medication abortion by mail in the United States** | |
| Finding | Stigmatized anti-abortion narratives. (U) |
| Illustration | “you’re basically killing a life” ^(p.182)^  “you’re automatically a slut” ^(p.182)^  “you’re being selfish” ^(p.182)^ |
| Finding | Centered the participant by making the process easy, prioritizing their needs, and treating them with respect (TelAbortion). (U) |
| Illustration | "[I appreciated the] convenience, lack of judgment. And not just lack of judgment, but it was friendlier. It was so much friendlier. It was not just nonjudgmental but just happy to help in the decision that I had had to make, because it’s not a pleasant decision. I don’t think anybody’s like, “Oh, yay. I get to go do this.” It’s shitty. But at the same time, if you’re doing that, you’re doing it for a good reason. And it was a much more pleasant experience. You can tell even from the way that the [TelAbortion website] is worded, they’re there to help people and they have that perspective on it instead of it being a nasty business to be associated with."^(p.182-183)^ |
| Finding | Expectation: Judgmental abortion provider. (U) |
| Illustration | “I did think that there would be a lot more judgment and stigma from the medical people that were operating the center.” ^(p.183)^ |
| Finding | Actual experience: Nonjudgmental abortion provider with TelAbortion. (U) |
| Illustration | “They just kind of trusted you as an adult that you knew that this decision was right for you and they help in a really nonjudgmental manner, which I wasn’t expecting.“ ^(p.183)^ |
| Finding | Expectation: Pressure to make a particular decision. (U) |
| Illustration | “I really thought it would be extremely negative and that I would second guess my decision and that people would try to convince me to not have the process.” ^(p.183)^ |
| Finding | Actual experience: Not feeling pressured to make a particular decision with TelAbortion. (U) |
| Illustration | “I realized that everybody who was participating and helped were really kind and really supportive, and nobody questioned my decision. And if anything, they just wanted me to feel comfortable while I was doing it.” ^(p.183)^ |
| Finding | Expectation: Community stigma. (U) |
| Illustration | “There’s billboards all over my city, just about, how life begins at conception and how babies have heartbeats at two days of—honestly, they’re very much not about abortion. So the idea of when they said that there was protestors outside really scared me because I don’t know how crazy those people are.” ^(p.183)^ |
| Finding | Actual experience: Not experiencing community stigma with TelAbortion. (U) |
| Illustration | “Where I live really played a role in it because of how people are so against it, and so being able to do it at home and nobody knew I was even doing it is very relieving.” ^(p.183)^ |
| Finding | Expectation: Difficulty accessing abortion care. (U) |
| Illustration | “In Tennessee, I would have had to drive to Nashville, a 45-minute drive and sometimes I probably would have even hit a brick wall. You never know.” ^(p.183)^ |
| Finding | Actual experience: Not experiencing difficulty accessing abortion care with TelAbortion. (U) |
| Illustration | “I was surprised at how just-- there was someone available. Someone was able to get me in right away. I didn’t have to jump through hoops or anything to do it… It was pretty pleasant. I was pretty surprised at how easy and simple it was made for me. I really appreciate that.” ^(p.183)^ |
| Finding | Expectation: Protestors |
| Illustration | “A lot of the clinics over there actually do get petitioners and protestors all the time. So a majority of the time when you are going to a Planned Parenthood out there, you are going to run into protestors and things like that. Just having to deal with that makes you feel worse about your decision and makes you feel more guilty about things.” ^(p.183)^ |
| Finding | Actual experience: No protestors with TelAbortion. (U) |
| Illustration | “It was nice because you do everything in the comfort of your own home. You don’t have to go to a clinic and possibly have protestors there waiting. Saying, “You’re going to do X, Y, Z,” or objectifying or looking at you any sort of way just because you’re seen going into a Planned Parenthood. Everything’s at your home. All you have to do is wait for a package. Nobody knows it’s from Planned Parenthood… You don’t have anybody’s views or opinions in the mix of what you’re doing. It’s basically your own personal business and that’s what I liked about it…You’re in the comfort of your own home.” ^(p.183)^ |
| Finding | Expectation: Hostile clinic environment. (U) |
| Illustration | “I went in there and it was weird because a lot of these [abortion] clinics are lockdown style and bulletproof glass. And I remember the one time like, “Man. This is kind of scary.” And I realized it was because they would perform abortions. And so that kind of informed my decision too, just my memory of going into the wrong clinic to try and get birth control and being like, wait, it feels weird.” ^(p.183)^ |
| Finding | Actual experience: No hostile clinic environment with TelAbortion. (U) |
| Illustration | “It was pretty cool, quote that “pretty cool”. No, it was just really nice to have this option to be in my home. I could have done a few things better. But at the end of the day, I was just very grateful that I had access to the service.” ^(p.183)^ |
| Finding | Expectation: Traumatic abortion process. (U) |
| Illustration | “I honestly thought it would just be a terrible process because I had this one friend who was 12, and she got pregnant. But at the time, she was far along, so she had to get surgery. And I don’t know, it just seemed like it really changed her. She just got so depressed, and it was such a painful experience. I thought, “I could never put myself through that.” She made it seem like it was terrible, which is why I was scared.” ^(p.183)^ |
| Finding | Actual experience: Non-Traumatic abortion process with TelAbortion. (U) |
| Illustration | “It just seemed like I overreacted towards the process, I guess. I thought it was going to be something so heavy, something so scary, but it really wasn’t. It was super easy. It was just getting your period and stuff like that. And I just want other girls to know it’s not as scary as you think it is. If it’s really what you want, it’s not scary. It’s just like getting your period.” ^(p.183)^ |
| Finding | Because of abortion stigma, this decision involved weighing risks. (U) |
| Illustration | “Because you had to get a referral for an ultrasound and stuff, and everybody that I talked to, I basically had to explain what I was doing and who it was for because they were just like, “Well, why isn’t your OB prescribing this?” So I had to explain to everybody like, “Because I’m getting a fucking abortion.”” ^(p.184)^ |
| Finding | Participants went to crisis pregnancy centers (CPCs) for their ultrasound, often not realizing that CPCs are designed to convince patients to not obtain an abortion. (U) |
| Illustration | "And I can tell that they’re against abortions and everything like that, so even though…I told her that I wasn’t going to keep it. And she didn’t have a nonchalant attitude about it…But I sucked it up, and all I really needed was the ultrasound, not their opinion…So I just went ahead and got the ultrasound." ^(p.184)^ |
| Finding | Even when accessing traditional medical care, they faced judgment. (U) |
| Illustration | "I had a woman that was doing my vitals…And she was talking about the baby, and talking about the baby, and asking me questions, if I had a name picked out and all of that. And after I said, “Actually, I’m getting an abortion. I just need this stuff done so that I can get the abortion.” And she gave me the meanest look. I told her, I said, “It’s my choice.” And she told me that there was a special place in hell for people like me. So that’s the discrimination that I’m talking about, seeing that people don’t know the situation. And they don’t know that I’m trying to escape an abusive situation." ^(p.184)^ |
| **Study: Kerestes *et al.* (2021)*.* “It was close enough, but it wasn’t close enough”: a qualitative exploration of the impact of direct-to-patient telemedicine abortion on access to abortion care** | |
| Finding | Her past abortion experience was unacceptable to her due to the loss of privacy she experienced while in the clinic and having a direct-to-patient telemedicine abortion provided an appealing alternative. (U) |
| Illustration | "It [the abortion clinic] isn’t too far from me. The issue was more so the privacy. And my past one that I had, it was exposed because I seen somebody there and they’d seen me there for so many hours. And they put two and two together, and then it turned into a really embarrassing experience. I live in this capital city, so I have access near my home. But it’s just such a small area in the waiting room. You just sit there. And people were there for numerous reasons, but it’s easy to guess, I guess.” ^(p.70)^ |
| Finding | The fact that privacy was so important to many participants highlights the internalized and social stigma surrounding abortion that is not a consideration for most other medical procedures or  healthcare services. (U) |
| Illustration | “because of the stigma, it’s not something that I really feel– I don’t feel super comfortable going to a clinic for it. And I … know a lot of people in the area, so it would’ve been very uncomfortable if I’d gone to a clinic specifically for that, like Planned Parenthood, because it would’ve had people asking me questions [laughter], which would have been uncomfortable.” ^(p.70)^ |
| **Study: Killinger *et al.* (2022)*.* Why women choose abortion through telemedicine outside the formal health sector in Germany: a mixed-methods study** | |
| Finding | Self-recrimination and shame for the unwanted pregnancy were internal motivations for requesting telemedicine abortion. (U) |
| Illustration | "[…] I feel very ashamed at the thought of discussing or rather sharing my decision to abort with so many people, that I do not know. I have the feeling that one will indirectly judge me and this, in combination with the thought of having the abortion carried out by the same people, feels unbelievably difficult and wrong to me." ^(p.9)^ |
| **Study: Kimport and Rasidjan (2023). Exploring the emotional costs of abortion travel in the United States due to legal restriction** | |
| Finding | Necessity of going outside of her existing healthcare system to ob-tain an abortion inspired feelings of uncertainty about the morality of her abortion. (U) |
| Illustration | "[T]o have had it done in [my] hospital, for me, I wouldn’t have had such a journey to get the medical validation. Because if you’re in a hospital, you’re kind of automatically assuming that this is a normal situation, because you’re in a facility, and they’re doing a procedure that they’re all trained in and knowledgeable of. And so, that uncertainty would have been off my plate. […] I would have automatically had the validation." ^(p. 3)^  “(I)f it had been an (in-state) hospital situation, I don’t think it would have felt so abnormal.” (p. 3)  "being forced to travel was “inhumane because I couldn’t be treated at a normal hospital with normal, you know, care and attention,” ^(p. 3)^ |
| Finding | Further described how the legal origins of them being denied abortion care were central to the emotional costs of travel. (C) |
| Illustration | "[...] used the language of being “cast out” of her hospital and her state when she needed abortion care." ^(p. 3)^  "her care providers could not offer her an abortion due to state law made her “feel very let down by my state and by the government.” ^(p. 3)^ |
| Finding | The source of the nega-tive emotions she and her husband experienced was state law. (U) |
| Illustration | "her state’s gestational limit law, “It’s forcing shame and stigma on you during what is already the worst moment of your life (of learning your fetus is unhealthy)." (p. 3)  "At no point did anyone that we knew personally try to put shame on to us—it was the state, but no one that we knew and loved did.” ^(p. 3)^ |
| **Study: Kimport (2019). Pregnant women’s experiences of crisis pregnancy centers: when abortion stigmatization succeeds and fails** | |
| Finding | CPC counselors indirectly stigma-tized abortion as a pregnancy outcome. (U) |
| Illustration | “who visited a CPC in Louisiana, spoke with explained that she was not concerned about the prospect of Renita raising her baby as a single mother because “Jesus will be there for the baby. That [he’s] all of our fathers.” ^(p.625)^ |
| Finding | Impact of the video [at CPC] in framing abortion interacted with Camille’s preexisting—and negative—beliefs about abortion. (U) |
| Illustration | "[Of] the actual abortion. Like, a doctor that shows you what it’s like from the inside. So, not like seeing the graphic part, but what it’s like from the inside during an abortion. So, not the bloody stuff and all that, just like a black-and-white screen that’s showing you how they – actually, they show you the utensils that they use and how they prop your uterus, just all that stuff, and how they suck the baby out and all that stuff. ^(p.626)^  “I already was uncertain, and it [the video] made me feel bad, you know. Just a lot. I already was uncertain, and I already know what I’ve been taught.” ^(p.626)^  “it was shocking to me because when they sent me in the room and I’m looking [at the video]. I was, like, well, man, I don’t believe in abortion. I’m not coming here to get an abortion.” ^(p.629)^ |
| Finding | Religious judgment the CPC staff invoked. (U) |
| Illustration | It was how “they throw the God stuff in.” ^(p.628)^  The female ultrasound tech and then the fatherhood coach are both standing at the door physically blocking our exit, and they’re just nailing us with, “God wants you to keep this baby. It’s a baby, not a fetus!” ^(p.628)^ |
| Finding | Insisted that the CPC was not a place that considered abortion a bad thing (Kimport2019, p. 13). (U) |
| Illustration | "I know I was talking for a while [to the counselor], but I don’t remember them saying that it’s [abortion] a bad thing. I don’t think it was somewhere to try to make you keep your child. They only asked if you want to keep [the pregnancy]. You know what I mean? It wasn’t somewhere to force pregnancy on you." ^(p.630)^ |
| Finding | Contributed to her perceiving the CPC itself as not antiabortion. (U) |
| Illustration | "It’s not like a pregnancy crisis center where they’re like crazy people that don’t want you to really have a clear choice. I think it’s just like, you know, like just free options. And they offer like classes that you can use and get points to get free baby stuff. You know, stuff like that. It was actually like a really nice place and the staff were super nice and it was all around like a super, super nice thing. "^(p.631)^ |
| Finding | Rather than express judgment or shock, the counselor seemed to take it in stride. (U) |
| Illustration | “she was just kind of saying, like, the way that things are and the way that the world is, it’s a wonder any of us are born.” ^(p.631)^  “It was helpful in the sense that it started me off the track of the guilt. Because I was feeling really guilty now about what had happened the two weeks before that, you know? And it [talking to the counselor] was just part of helping me get over all that.” ^(p.631)^ |
| Finding | Began sharing “information” about the purported risks of abortion. (U) |
| Illustration | “you could die delivering [a baby],” “I wanted to just be okay with my opinion. I don’t want to second-guess anything. I thought I was doing the right thing and I still think I was.” ^(p.632)^ |
| Finding | Rather than tap into an existing internalized stigmatization of abortion, these religious references had no effect. (U) |
| Illustration | “everybody has a purpose in their life or something. It seemed, like, very religious sounding sort of the way that, you know, every life is precious.” ^(p.632)^ |
| Finding | Contextualizing misinformation through information of risk of pregnancy. (U) |
| Illustration | “were just telling me how far I was and what’s the stages, and what’s the risk [of abortion], both mental risks and physical risks, and all of that stuff. They were just telling me how it was going to be.” “sometimes you can get very depressed from doing this because it’s a big decision to make and some people feel like they made the wrong decision.” “She was just saying, you know, it could be infections which is a small percentage. But, sometimes you can have, you know, an infection. Your cervix can be infected or whatever. Or, you could die. Just like any surgery.” ^(p.632)^  “come out traumatized, they don’t want to have sex, they’re emotionally, you know, messed up for a long time—all these very bad, like, you know, very likely things that could have happened if I were to go through with [the abortion].” ^(p.633)^ |
| Finding | Neither the health “information” nor the religious claims changed Sierra’s mind about abortion as the right choice for her. (U) |
| Illustration | “I was understanding, I think, of what they were trying to tell me, but I wasn’t swayed at all because I understood my situation and what I needed to do for myself.” When she tried to share her thinking and her reasons for seeking an abortion with the CPC counselor, “they said, ‘okay, but you, you know, you could still make it work if you really wanted it.’ And, like, I could, of course. I could also go jump off a bridge if I wanted to. I mean, I don’t know why they were still trying to convince me that I should change my mind.” ^(p.633)^  “I’m prochoice because I don’t feel like my decisions and my viewpoints on the world should have any impact on anybody else’s decisions.” ^(p.633)^ |
| **Study: LaRoche *et al.* (2021*).* “We have to make sure you meet certain criteria”: exploring patient experiences of the criminalisation of abortion in Australia** | |
| Finding | For several others, the criminalised nature of abortion made them feel defiant in the face of what they perceived to be un unfair law. (U) |
| Illustration | “I was very much like, that’s stupid and you’re not stopping me. I’ll figure it out.” ^(p.4)^ |
| Finding | The criminal status of abortion felt like a moral judgment and contributed to the feeling that abortion was something that should not be talked about. (U) |
| Illustration | “I believe that they’re very hush-hush and they have to be because it is illegal to have abortions in NSW.” ^(p.4)^  “Yeah, [the community organisation that helped me find services] said where I was going to [have my abortion] was a legal place, but what I was doing was illegal... They said just not to speak too much about it.” ^(p.4)^ |
| Finding | Participants felt that the current laws were punitive rather than protective. (U) |
| Illustration | "I think it makes no sense and I think it’s completely ridiculous and super stigmatising… I see it as healthcare that people choose to make some kind of a moral playground.” ^(p.6)^ |
| **Study: LaRoche *et al.* (2020)*.* ‘‘We’ve got rights and yet we don’t have access”: exploring patient experiences accessing medication abortion in Australia** | |
| Finding | Judgmental and unhelpful interactions with clinicians. (U) |
| Illustration | ‘‘[The doctor] just sort of told me the risks...Like, you know there’s a risk of infertility and all sort of stuff...So I think she was definitely trying to skew me away from it...But to me, that sort of reaffirms this opinion in my head that like, I’d really stuffed up and I was doing a really bad thing. And it just sort of reaffirmed all this negative. So it’s taken me so long to realize that actually what I did wasn’t a bad thing.” ^(p.3)^  ‘‘I told her I couldn’t have the baby, and she said it was a miracle that it even happened. I was 28. She advised me that at my age [I should] think about it as my one opportunity... .I asked her where I could get [an abortion] and she said they didn’t do that there and she didn’t know anyone locally who [did]. I wanted to understand my options, but she didn’t tell me them which was a lie. There are options.” " ^(p.3)^ |
| Finding | Many participants, abortion stigma influenced if, when, and to whom they felt comfortable disclosing their pregnancy status. (C) |
| Illustration | ‘‘So then I have to lie to people about why I’m not going to be able to pay rent or I’m not going to be able to pay for this. Because I can’t exactly just tell everyone, ‘Oh I’m pregnant’ and get judged." ^(p.3)^ |
| **Study: LaRoche and Foster (2018). Exploring Canadian women’s multiple abortion experiences: implications for reducing stigma and improving patient-centered care** | |
| Finding | She found out that she was pregnant for a second time and made the decision to have an abortion, Miranda travelled to another city for her procedure because she was concerned about being judged. (U) |
| Illustration | “I didn’t want to go back to the same place where I had [the previous] abortion ‘cause I felt they were going to judge me.” ^(p.4)^ |
| Finding | Being surprised about the positive care that she received for her second termination, stating emphatically. (U) |
| Illustration | “I didn’t feel judged even though it was my second abortion.” ^(p.4)^ |
| Finding | Participants explained that they had amplified shame and stigma because of their need for more than one abortion. (U) |
| Illustration | “I guess, for me the thing that I felt sort of weird about was the fact that I had three [abortions]. I have some shame around that, sort of like, you think that I could get it together better on the birth control front" ^(p.4)^  “I felt very stupid for getting pregnant again. And I felt really bad because I was in the same situation. I was trying to focus on myself and still growing up. I just felt very immature, irresponsible, and I felt really guilty. And bad at first. But I felt very relieved after the procedure. And I knew that it was the right choice for me.” ^(p.4^ |
| **Study: Lerma *et al.* (2023). Abortion Clients’ Perceptions of Alternative Medication Abortion Service Delivery Options in Mississippi** | |
| Finding | Abortion stigma: Anticipated guilt and shame from local provider |
| Illustration | “I would have been a bit embarrassed, personally, and that’s because of own my poor decisions in  conceiving and not being married. So, I would have felt bad.” ^(p.4)^ |
| Finding | Many participants described the additional benefits of avoiding perceived stigma and judgment from those at the facility, protesters, and people they needed to involve in accessing care, such as a family member who helped with childcare or a friend they asked for a ride. |
| Illustration | “Not actually having to go to the clinic and people seeing your face, and know[ing] who you are, and what you’re doing, and judging you. Even though they might not say anything, people can still blame and judge you.” ^(p.4)^ |
| Finding | Others described the guilt and shame they would feel disclosing their abortion to their regular provider, such as a 28- year-old Black woman, who did not return for follow-up care because she no longer had pregnancy symptoms. |
| Illustration | “Obviously, abortion has a stigma around it in any way, shape, or form. I think that people probably would not understand. More so for me, I think it would be that [my doctor] will be like, ‘You’ve got six kids, you should know how not to get pregnant, right?’ I didn’t want to have that conversation.” ^(p.5)^ |
| **Study: Mahanaimy *et al.* (2022). What constitutes a good healthcare experience for unintended pregnancy? A qualitative study among young people in California** | |
| Finding | Participants expressed gratitude for empathy, non-judgemental and attention to the unique circumstances they identified in interactions with providers. (U) |
| Illustration | “[The providers] were helpful. I felt they took care of me just like they took care of anyone else who was pregnant.” ^(p.7)^ |
| Finding | Feeling criticised and unsupported by providers that were rude, dismissive or out-right judgemental. (U) |
| Illustration | “I felt like I was treated really harshly by my OB/GYN. So, I went to one visit, and I think I had something to prove, like, “Oh, I’m in a relationship. I’m not a teen mom.” Even though if I was, I think she [should] still treat me with respect. [ …] I was like, “Is there any way we can schedule one of these [check-ups] after normal work hours, so [my boyfriend] can come?” [… ] She goes, “What? Is he invisible?” And she was accusing me of actually not having a boyfriend.” ^(p.7)^ |
| **Study: Makleff *et al.* (2023). Typologies of interactions between abortion seekers and healthcare workers in Australia: a qualitative study exploring the impact of stigma on quality of care** | |
| Finding | Creating barriers to abortion access. The main behaviors were providing insufficient information to abortion seekers, delaying or gatekeeping access to the service, or denying care altogether. |
| Illustration | “I said [to the GP] […] ‘We’d like to access termination services. […] I’ve tried to look into it, but I don’t know who to access, who to talk to. Is that something that you can help me navigate?’ And he just kind of got a bit uncomfortable and just said, ‘we don’t do that here. There’s some GPs that do.’ […] I was literally on the phone with him, for I think three minutes in total. […] I left more confused I think than anything. And then they’re like, right, here’s your bill for $75” ^(p.5)^  “[The receptionist] asked me how far along I was. […] I thought about six weeks. And she said, well, we can’t actually do anything till nine weeks.” ^(p.5)^  “was kind of hesitant to give it to me, and he’s like, ‘No, I’d rather like you think about it and come back Monday’” ^(p.5)^  “As one said, “[when booking] I said, ‘Oh, by the way this is for a termination of pregnancy. That’s why I need the appointment.’ And the receptionist on the phone just said ‘no, we don’t do that.’ But she was very short and very abrupt.” ^(p.5)^ |
| Finding | Judging, blaming, questioning, or punishing abortion seekers. Participants described direct and Implicit communications from healthcare workers that made them feel blamed, judged, or punished for their pregnancy and subsequent abortion. |
| Illustration | “you’ll regret [an abortion] if you’re 30” ^(p.5)^  “or asked, “If I told you [that] you were having twins, would it change your mind?” ^(p.5)^  “[The GP said] ‘you’re not allowed to do this [abortion] again.’ […] Scolding me like I was some young girl who’d been super irresponsible, not like an adult woman who is aware of, like her decisions” ^(p.5)^  “[If] I had adequate information, it would mean I could get better pain medication in advance rather than having to go to the emergency department in the midst of COVID” ^(p.5)^  “I think the GP should have […] had more information and less opinions […], like they would with any other procedure” ^(p.5)^ |
| Finding | Interactions that do not respond to abortion seekers’ emotional and information needs. Participants described interactions with healthcare workers that did not respond to their emotional and information needs at different points in the abortion pathway. |
| Illustration | “[I] would have much preferred surgical but I don’t feel like that was like adequately explained to me at the time. […] I remember seeing on my Medicare bill that the doctor had charged me for like, $180 for counselling. I don’t feel like, you know, I was counselled in any way. In some ways I’m grateful for the fact that she didn’t try to talk me out of [an abortion]. But in other ways, I sort of feel like that [appointment] should have involved an exploration of the process of a medical termination versus a surgical termination” ^(p.6)^  “I feel like they definitely could have done more to comfort me and just make the process a little bit easier for me. […] To have a bit more time with the doctor to explain the proce-dure to me […] I just feel like that would have comforted me” ^(p.6)^  Another concurred that emotional support was “really, really lacking. It was very like, very medical  focused” ^(p.6)^  “Everybody in that clinic was cold […]. It was horrible. From the moment I got there to the moment I left” ^(p.6)^ |
| Finding | Making assumptions about reproductive intentions and related preferences for care. |
| Illustration | “[the sonographer] was acting very excited for me, and asking if I wanted images saved, and asking when my due date was, and that sort of thing. Which was really really hard to deal with” ^(p.7)^  “It was horrible. I faked it and I just played along because I didn’t want to have that conversation [about abortion]. That felt like taking a bullet” ^(p.7)^  “You think you’d read the room and maybe think, the girl might need some options. […] I wouldn’t go back to that GP.” ^(p.7)^  “Maybe there were assumptions made about me, that I’d been really firm on my decision. […] I wish someone had a dug a little bit deeper” ^(p.7)^  “They also were really pushing an IUD onto me, and I found that really opportunistic” ^(p.7)^  “they are reminding [you about contraception] every time, and […] you feel that pressure, feel like you have to do it” ^(p.7)^ |
| Finding | Minimized interactions that compromise the quality and safety of abortion care. Many participants described receiving insufficient procedural or aftercare information, follow up, or time to support the recovery process. |
| Illustration | “I just didn’t really feel like I should have been there, but I didn’t know how else to go about it rather than go to the GP […]. I just don’t feel like they prepared me for just how much [bleeding] it would be” ^(p.7)^  Further, several participants described being “pushed out the door” ^(p.7)^  “They hadn’t called [my partner] or anything, and I was still quite, you know, drowsy, [and] stumbling in the car park. […] My partner [happened to] look up in his rear vision mirror and saw me” ^(p.7)^  “I was supposed to have antibiotics that night […] Why didn’t they give [my partner] the information?” ^(p.7)^  “I didn’t receive any follow-up support. […] I think that they maybe, should have offered me some-thing, or even just a follow-up phone call” ^(p.7)^ |
| Finding | Actively helping people access abortion care. Some providers, who did not themselves provide abortion care because of health systems constraints, proactively provided abortion information, or helped organise the referral and subsequent appointments. |
| Illustration | “[the GP] went through what the process was, and that there were no options available for me in my local area, and that she would have to refer me to somebody else. […] Yeah, she got me in for the bloods next, all straight away, and then over to the ultra-sound, and then she gave me follow-up call. I think she tried to call me four times […]. And I was amazed that, you know, somebody would take so much time out of their day just to check on me, to make sure that I had […] got the referral” ^(p.7)^  “She was like, ‘you know you have options […]. You can go to the hospi-tal tonight, if you want to, or I’ll call your GP and we can book you in.’ […] She made a call to my GPs office, my GP just happened to be there working late and they got me booked in […]. That was like really efficient and a relief, it meant that I came away from the appointment with a clear plan, and I think that was really important to me at the time. Like yeah again, the sense of agency and some sort of picture of what was about to happen” ^(p.7)^ |
| Finding | Actively validating abortion decision. Participants also experienced interactions that actively validated and supported their decision to have an abortion with different types of healthcare workers along the care pathway. |
| Illustration | “Some providers actively verbalised their support, for example saying, “there’s no right or wrong’” (ID18), “we don’t judge anybody” (ID24), and “you have every right to be here” (ID08).” ^(p.8)^  “[the doctor] just listened and she just said, this is your choice. You know what you need, you know your life, you know what you’re capable of, and what you’re doing isn’t wrong. You’re just making a decision. […] She just said all the right things” ^(p.8)^  “[The GP] didn’t ask, like any questions that would even like imply judgment like ‘How did this happen’. […] She was just incredibly supportive and really like outcome [and] action oriented” ^(p.8)^ |
| Finding | Interactions responsive to evolving emotional and information needs. Many participants described interactions in which healthcare workers offered comfort and emotional support during and after the abortion service, supported an informed decision-making process, or ensured the ser-vice aligned with their needs and preferences. |
| Illustration | “[The doctor] gave me the options. We weighed them up together and then made the decision together. I sort of already knew what I wanted to go towards, but he was really good […] in talking to me about […] both options in detail” ^(p.8)^  “I hope everyone gets access to a GP as lovely as mine was. […] Having access to some-one who will take the time to sit with you and go through all the options. Like, we were certainly extending beyond standard appointments. In my time with her we were really going through things and understanding what was going to happen” ^(p.8)^  “nurse ended up actually coming into the procedure with me and staying with me until I woke up. Incredible” ^(p.8)^  “they treat you as a person, not a number” ^(p.8)^ |
| Finding | Aligning abortion provision with client reproductive intentions and service preferences. Participants described positive interactions in which the provider sought to understand their reproductive intentions and service-related preferences, and then aimed to provide care accordingly. |
| Illustration | “I felt like [the GP] was quite kind, you know, and like he asked me what was I wanting to do” ^(p.8)^  “[My partner and I] were trying to decide what to do, which was very scary because obviously we weren’t expecting this [pregnancy]. So, the doctor gave me some time to think. […] Gave me a timeline, if I was to terminate, what sort of termination I would have, and also gave me some vitamins if I was to go ahead with the pregnancy” ^(p.8)^  “I really didn’t want [to] get an ultrasound at all […], especially by myself. […] They were like, ‘some-one will come in with you. You don’t have to like, look at anything [on the screen].’ And they were just really, really good at calming all the things that I was bringing up” ^(p.8)^  “I said, you know I want to have Implanon [the contraceptive implant] put in on my arm. And [we] just sort of talked through that.” ^(p.8)^ |
| Finding | Providing holistic and high-quality care to ensure safety. Some participants described receiving sufficient aftercare information, follow up, and supportive care during recovery, which prepared them to manage their abortion safely. |
| Illustration | “It was [not the doctor but] the pharmacist that went into detail and said, if this happens, then you gotta go into emergency and […] gave me the […] tools for me to make an informed decision at home, or when to escalate something if something was [going] wrong” ^(p.9)^  “There was a nurse like right next to me when I woke up […] I think that was very good and comforting to have someone there immediately upon waking up. And I think it was nice, they had like music playing in the recovery” ^(p.9)^  “I woke up and then had a nice male nurse that was sort of fussing around. I think that [they had] snacks, and they sort of say, have something to eat. So that, that [I] was just feeling as though it was a normal, fine thing to be doing” ^(p.9)^  “because it was [about to be] a long week-end, they called me [on] the Friday, to make sure I was  all okay” ^(p.9)^  “She’s just going to go through, probably do some blood and stuff again, just to make sure that it was successful” ^(p.9)^ |
| Finding | Gestational age limits influenced the experiences of many participants. Those nearing the 63-day gestational age limit for medication abortion had an urgent need to find timely appointments with a willing provider – a particular challenge among rural participants. |
| Illustration | “That really put in perspective, the significance of this medical board. Because even though we had made the decision to terminate the pregnancy, it wasn’t our decision. If that makes sense, it was someone else’s decision” ^(p.9)^ |
| Finding | Some participants lacked a choice in method due to limited provision in rural areas. |
| Illustration | “There was no one who did [surgical termination of pregnancy] in North Queensland. If I wanted the surgery, I was going to have to go [over 1,500 km] to Brisbane” ^(p.9)^ |
| Finding | Healthcare workers were rude, rushed, uncompassionate, and showed little concern for protecting patient privacy. |
| Illustration | “if there were more options […] there’d be a bit more competition. But they’re the only [abortion provider]. So, they really get to pick and choose as they want. The facili-ties were terrible, the support was terrible” ^(p.9)^ |
| **Study: Margo *et al.* (2016)*.* Women’s pathways to abortion care in South Carolina: a qualitative study of obstacles and supports** | |
| Finding | Anticipated stigmatizing reactions from others, which led to self-protective secretive behavior. (U) |
| Illustration | "With appointments like this, it’s more hush-hush…. You don’t really want to go into detail, because you don’t know how some people might react. It’s kind of the same way with mental health care, and everyone’s hush-hush about that.... You don’t want people to know you were here.” ^(p.204)^ |
| **Study: Monchalin *et al.* (2023)*.* A qualitative study exploring access barriers to abortion services among Indigenous Peoples in Canada** | |
| Finding | This resulted in internalized shame amongst participants surrounding their abortion decision. |
| Illustration | “where I’m from, Anishnaabe families … it’s all about having kids and grandkids.” ^(p.3)^  “Being Haudenosaunee … I call it an ‘old value’ … as a woman, one of your jobs is to have children… raise children, bear children, and there’s that old world stigma that’s like… if you don’t have children and have a family and raise a family, what are you doing? … Traditionally, birth control, you’re not supposed to do that because you’re going against the Almighty Creator and trying to play God almost by controlling things. If they’re going to happen, they’re going to happen, so why are you trying to stop it?” ^(p.3)^ |
| **Study: Purcell *et al.*** (Purcell et al., 2020)***.* Toward normalising abortion: findings from a qualitative secondary analysis study** | |
| Finding | Surprise that the experience was not as bad as she might have expected. (U) |
| Illustration | "…the whole experience was actually OK, I wasn’t like traumatised by it and I wasn’t… I was quite practical about it. And then they [abortion providers] made me feel ‘yeah, if that’s what you think is right for you then that’s fine, we’re not going to, you know, be like “no, you’re doing wrong”’. So, you know, they assured me that it’s my decision and if that’s what I want to do then that’s fine." ^(p.1354)^ |
| Finding | Explain that she did not feel abortion should be silenced, and that she was happy to discuss her experience. (U) |
| Illustration | "I freely share my experience with whoever wants to listen. I mean, for me it’s not taboo, I speak with my friends about it, with my friends who are male also. I just tell them: ‘you know what happened? I was pregnant.’ I feel free to speak about it because, you know, it’s happened to other people. And the leaflet I got in the hospital says that at some point one third of women in the UK will have a termination of pregnancy. So plenty of women went through this." ^(p.1355)^ |
| Finding | Not feeling embarrassment. (U) |
| Illustration | "…it was actually quite a good experience, I didn’t feel like, y’know, ashamed of what I was doing. Nobody made me feel kind of bad or guilty for what I was going through."^(p.1357)^  "I’m quite close to [work colleagues] as well, so I don’t, like, I wasn’t embarrassed by the situation, I would rather tell people than try to cover it up. [ …] just in terms of, [I] would rather less people knew than more, not ’cause I’m embarrassed, but I just don’t want it to be something that’s kind of constantly brought up or made a big deal of…" ^(p.1357)^ |
| Finding | Potential for judgement was also apparent in her account of her interactions with health professionals. (C) |
| Illustration | "I didn’t feel kind of judged which I was a bit worried about, I didn’t want people to be like: ’Oh,’ like: ’She’s made the same mistake again’ kind of thing. Which was really nice, it wasn’t – I didn’t feel kind of patronised or like looked down upon." ^(p.1357)^ |
| **Study: Rafferty and Longbons (2021). #AbortionChangesYou: a case study to understand the communicative tensions in women’s medication abortion narratives** | |
| Finding | Women noted feelings of shame, embarrassment, worry, or fear as some of the reasons for not telling others. (U) |
| Illustration | “I never told the father and I don’t intend to” ^(p.1490)^ “I don’t know if I will ever tell my husband and children about what I did” ^(p.1490)^  “I couldn’t talk to my family” ^(p.1490)^  “I feel like I have no one to speak to about it since he doesn’t think about it the way I do” ^(p.1491)^ “I try to talk about it with my family and the baby’s dad but they all tell me it’s in the past” ^(p.1491)^ |
| Finding | Women’s desires to talk about their abortion, others (e.g., the baby’s father, their husband, family members) refused to engage in conversation with them. (U) |
| Illustration | “I love my husband but it is beyond difficult for me to talk to him about this, because I know he wants nothing more than to just move on from this” ^(p.1490-1491)^  “My close friends know here but I don’t really feel I can talk to them about it. I don’t feel like i can talk to anyone about it” ^(p.1491)^  “I feel like I have no one to speak to about it since he doesn’t think about it the way I do” (9-8-15), and “I try to talk about it with my family and the baby’s dad but they all tell me it’s in the past” ^(p.1491)^ |
| Finding | Isolation and silence leading up to her own medication abortion continued to pervade after the abortion. (U) |
| Illustration | “But I haven’t really been able to share the true regret and near constant jealously of my loved ones engagements or pregnancies” ^(p.1491)^  “I knew I had to have an abortion, but these feelings I have right now I never imagined I’d have. I don’t want to go out, I don’t want to tell anyone, all I feel like doing is crying” ^(p.1491)^ |
| Finding | Silence was often described as being frustrating and challenging. (U) |
| Illustration | “I feel like I’m living a lie I get up get ready for work get my family up like normal the days go on like normal but I’m not normal I killed my baby I’m a monster!!” ^(p.1491)^ “As a mom I feel like a monster and I have to act like nothing happened” ^(p.1491)^ |
| Finding | Felt muted by their loved ones because of the centripetal dis-courses of shame and embarrassment associated with abortion. (U) |
| Illustration | “I tried to find an OBGYN that could see me ASAP. I went in and told them I had a miscarriage because I was ashamed of the truth of what I did” ^(p.1491)^  “I am in desperate need of assistance and I am too embarrassed to attend an in person support group” ^(p.1491)^  “And when I got home, I had to hold it all in. I was so ashamed of my choice. I couldn’t let anyone know” ^(p.1491)^ |
| **Study: Warren *et al.* (2022). “I felt like I was a bad person… which I’m not”: Stigmatization in crisis pregnancy center** | |
| Finding | How the clinic, in contrast to the CPC, did not induce guilt through enacted stigma. (U) |
| Illustration | "But at [abortion and medical clinic] when they did the ultrasound, they did it and didn't say anything. They were really, the first thing she said, you want to look at it? And I was like, no…So, yeah, she didn't make me feel uncomfortable at all and they were really supportive…Didn't make me feel like I had to look at the ultrasound or feel guilty for not looking at it, or whatever." ^(p.5)^ |
| Finding | Shift the conversation away and avoid the discomfort of a stigmatized choice. (U) |
| Illustration | "But our conversation in that room, I was so uncomfortable, I almost had to feel like I was, I had to agree with them just in that moment for them to get off my back. Because I felt like they were down my throat. And they kept asking me over, and over, and over again. Are you sure?" ^(p. 5)^ |
| Finding | When clients disclosed their intentions, they were met with criticism and social distancing. (U) |
| Illustration | "I feel like they were trying really hard to make me feel supported, but in the end, whenever I left I just felt manipulated. Because before I was like so sure and after leaving I just felt like I was going to be a bad person for choosing to have an abortion, which I'm not." (Warren2022pdf, S. 5) "She didn’t want to talk to me, she was just like … I abandoned the baby … She was like “yeah you are, you’re just killing a baby, it’s an innocent child”, and just saying stuff like that. Saying stuff you wouldn’t just say. And it’s just like, “why would you say that to me?”" ^(p. 5)^ |
| Finding | Fear of judgement through HPS at CPCs. (U) |
| Illustration | "I was even hesitant when I told the guy, he was just like don't tell them you're getting an abortion. That's how bad they made us kind of feel about it, we were just like we're not going to tell them we're going to get an abortion. We're not going to tell them anything else because we didn't want to them to downgrade us." ^(p. 5)^ |
| Finding | Religious identity as giving her to tools to resist stigmatization by CPC staff. (U) |
| Illustration | "I did express I do have a strong belief in God, and that’s part of the thing I was struggling with, but there was a statement made by the lady where she was like, “Well you know God wouldn’t want …” you know blah, blah. I’m like, “Well, I’m sure I can work that out with God.” … There’s various reasons why I’m choosing this decision I have thought about, this is God’s plan, it was meant to be like this." ^(p. 5)^ |
| Finding | Clients relied on their social networks to offset the stigmatization that occurred at a CPC. (U) |
| Illustration | "After leaving I just felt like I was going to be a bad person for choosing to have an abortion, which I'm not. But-  Interviewer: Yeah. How did you end up resolving that feeling?  Natalie: Talking to my sister a lot and my parents, my boyfriend and just friends that truly supported whatever decision I was going to make and didn't have a hidden agenda like they did." ^(p. 5 - 6)^ |
| **Study: Wilson-Lowe *et al.* (2024). Seeking support for abortion online: a qualitative study of women’s experiences** | |
| Finding | Without this knowledge, viewing online accounts was presented as a way to reduce feelings of loneliness without feeling obliged to make public that they had undergone, or were about to undergo an abortion, thereby avoiding potential stigmatising interaction. |
| Illustration | “Well, I didn’t want to tell my friends, because three quarters of them are Catholic […] and I didn’t want to be judged […] So I think it [the decision to go online] was more, it was all focused around the fact, I didn’t want people that were really close to me to know.” ^(p.3)^  “I wanted real people with real situations and experiences […] rather than the kind of … I suppose the kind of … I don’t know, the official kind of advice or information that they give you kind of … I always felt like you would get more real answers from the people that have been through it.” ^(p.3)^ |
| Finding | Anti-abortion rhetoric encountered online included harmful stereotypes and threats of violence, with some participants expressing that this negatively impacted their abortion experience and self-perception more broadly. |
| Illustration | “Then you hear ‘murderer’, you hear horrible derogatory terms towards women and threats and that sort of thing, they stand out and they’re really scary, and that’s kind of what warps your idea about all these things. […] Because you have seen all these horrible comments, you have convinced yourself that they’re right, that you are doing the worst thing in the world.” ^(p.3)^ |
| **Study: Woodruff *et al.* (2020). Experiences of harassment and empowerment after sharing personal abortion stories publicly** | |
| Finding | Respondents described the benefits of these positive experiences, such as the gratification of helping others who had had abortion. (U) |
| Illustration | “The first time I shared my abortion story on stage, I had women come up to me crying and hugging me. Several of them had never told anyone before that they had an abortion previously because they were too ashamed or felt guilty. They said my story helped them realize that they didn't need to feel shame or guilt.” ^(p.4)^ |
| Finding | Reported the satisfaction of having an impact on policymakers. (U) |
| Illustration | “I was asked to testify in [state] about a 20 week ban. I shared my story, and one lawmaker… later told the representative from the ACLU who was with me that my story made him change his vote.” ^(p.4)^ |
| Finding | Reported that sharing their stories publicly lessened their own feelings of shame or guilt. (U) |
| Illustration | “It has helped me to personally continue to unlearn the internalized shame and stigma associated with my abortion story. The more I have opened up about my story and the more I have gotten positive feedback from others — it reminds me that I am doing the greater good. I no longer feel my internalized shame.” ^(p.4)^ |
| Finding | Harassment actually encouraged them to continue sharing, as it demonstrated that harmful antiabortion attitudes are still prevalent. (U) |
| Illustration | “When I receive harassment, it makes me want to be even more bold to normalize the experience.” ^(p.4)^  “These [negative] experiences have strengthened my resolve to share as often as I am able to do so. As an older person, I feel I have nothing to lose, whereas young people risk so much more because of the stigma. I see the harassment as emotional and psychological violence, and I want to do all I can to stop the brutality.” ^(p.4)^ |

*C= credible, U= unequivocal*
