# Supplementary material for: Abortion stigma among people seeking abortion care in high-income countries: a mixed-methods systematic review
Source: Sex Reprod Health Matters. 2026 Jul 27;33(1):2709964. doi: 10.1080/26410397.2026.2709964 (PMC13660548; doi:10.1080/26410397.2026.2709964)
Supplement: Supplementary file 4 [file ZRHM_A_2709964_SM9490.docx]

| Study | Measurement tool | Prevalence (%) /  Level of Stigma (Mean, SD) | Explored Associations with correlations |
| --- | --- | --- | --- |
| **Biggs et al.** (2020)**^3^**  Country: USA  Sample: n=928 | Adapted ingle-item stigma measure (Major and Gramzow (1999)   - validity/reliability not reported - score range: 0-4 | - community abortion stigma: 60% - abortion stigma from close others: 56% | Baseline perceived abortion stigma   - African American/Black participants reported lower perceived abortion stigma than White participants - People who told two or more people about their abortion-seeking perceived lower overall and community stigma - Encounter with clinic protestors was associated with higher perceived stigma compared to those who did not see protestors   Five-year perceived abortion stigma trajectories   - Stigma decreased significantly over time   Factors significantly associated with higher stigma:   - Married status (vs. never married); β=0.25, 95%CI: 0.03-0.47 - Religion Protestant (vs. none) β=0.20, 95% CI: 0.06-0.35 - Religion Catholic (vs. none): β=0.28, 95% CI: 0.09-0.48 - Southern U.S. residence (vs. West): β =0.29, 95% CI: 0.10-0.47 - No prior abortion: β=0.20, 95% CI: 0.07-0.33 - Greater difficulty deciding to have the abortion: β=0.10, 95% CI: 0.05-0.14 - History of depression or anxiety: β=0.20, 95% CI: 0.06-0.35   Factors significantly associated with lower stigma:   - African American/Black participants (vs. White participants): β= -0.37, 95% CI: -0.54,-0.20 - Higher emotional social support: β = -0.05, 95% CI: -0.06- (-0.03) - Older age (vs. ages 14-19): β= -0.19 - (-0.31)   Psychological distress   - High perceived abortion stigma at baseline was associated with an increase in the odds of psychological distress years later (aOR = 3.98; 95% CI: 1.39-11.37) - Abortion disclosure to others had significantly higher odds of psychological distress than those who told no one (aOR = 2.22 CI: 1.13-4.37) |
| **Chesnokova et al.** (2024)**^1^**  Country: USA  Sample: n=70 | **ILAS scale**   - validity/reliability not reported - score range: 0-4 - Subscales: - Worries about judgment - Community condemnation - Self-judgment - Isolation | **Total experienced stigma**  M=1.21 (SD=0.63)  **Subscales**   - Worries about judgment: M=0.65 (SD=0.97) - Community condemnation: M=1.47 (SD=1.19) - Self-judgment: M=1.87 (SD=0.82) - Isolation: M=0.81 (SD=0.93) | Factors significantly associated with *higher* stigma   - Anxiety: ß = 0.49 (0.078, 0.893), p=0.02)   Factors significantly associated with *lower* stigma   - Trust in the provider: ß= -0.02 (-0.030 -0.004), p=0.013 - Partnered, living together (vs. single): ß = -0.44 (-0.839, -0.041), p=0.031)   Not associated factors   - Reasons for ending a pregnancy (i.e. rape/incest; medical problems; relational problems; economic hardships) (Ref.: Pregnancy timing and spacing) - Age, race/ethnicity, income, BMI, parity, gestational age - Relationship status: partnered but not living together |
| **Hanschmidt, Nagel et al.** (2018)**^1^**  Country: GER  Sample: n=148 | **ILAS scale**   - R (=0.78) - Cronbach’s α (=0.94) - score range: 0-4 - Subscales: - Worries about judgment - Community condemnation - Self-judgment - Isolation | **Subscales:**   - Worries about judgment: M=0.65, SD=0.75 - Community condemnation: M=0.54, SD=0.79 - Self-judgment: M=1.62, SD=1.04 - Isolation: M=0.67, SD=0.74 | Factors significantly associated with *higher* stigma   - Higher gestational age is associated with more self-judgment: β=.20, B=0.05 (0.01– 0.09)) - No perceived low fetal survival chance:   - More worries about judgment: β = -.20, B=-0.30 (-0.54 – -0.08)   - More self-judgment: β = –.28, B= -0.59 (-0.95 – -0.27)) - Lower perceived partner support:   - More isolation: β = –.34, B= -0.23 (-0.38 - (-0.10))   - More community condemnation: β = –.28, B= -0.21 (-0.38 – (-0.04))   Not associated factors   - Age - Income - Religious affiliation - Time since abortion (on any subscale) - Previous abortions or live births |
| **Hanschmidt et al.** (2018)**^1^**  Country: GER  Sample: n=148 | **Subscales of the ILAS scale**   - score range: 0-4 - Subscales: - Worries about judgment, α = 0.93 - Self-judgment, α = 0.85 | NR | Factors significantly associated with worries about judgment (as independent variable):   - Depressive symptomatology (PHQ-9): ß= 0.17 (0.02–1.75)*   Factors significantly associated with self-judgment (as independent variable):   - Grief (ICG): ß= 0.49; 4.22–9.59)* - Subjective posttraumatic stress (IES): ß= 0.40 (4.66–12.08)* - Depressive symptomatology (PHQ-9): ß= 0.27 (0.48–1.86)* |
| **Kerns et al.** (2022)**^3^**  Country: USA  Sample: n=353 | **Single Subscales of the ILAS**   - score range: 0-4 - Subscales: - Self-judgment, α = 0.84 - Community condemnation,α = 0.78 | **Subscales:**   - Self-judgment: M=1.7, SD=1.1 - Community condemnation : M=1.4, SD=0.8 | Self Judgement was significantly associated with grief and mental HRQOL but not with post-traumatic stress   - More grief (B=2.4 (0.4-4.5), p=0.02) - Mental HRQOL (B=2.0 (0.0-4.1), p=0.05) - Post-traumatic stress (B=0.7 (-1.9 to 3.3), p=0.58)   Perception of community condemnation was found to be not associated with grief, mental HRQOL, and post-traumatic stress   - More grief (B=-0.7 (-3.3 to 2.0), p=0.60) - Post-traumatic stress (B=0.4 (-2.9 to 3.6), p=0.83) - Mental health (B=-0.7 (-3.5to 2.1), p=0.60) |
| **Mehta et al.** (2019)**^1^**  Country: USA  Sample: n=587 | **ILAS scale**   - α= 0.8 – 0.9 for each subscale - score range: 0-4 - Subscales: - Worries about judgment - Community condemnation - Self-judgment - Isolation | **Total experienced stigma**  M=1.35, SD=0.63  **Subscales:**   - Worries about judgment: M=0.86, SD=0.86 - Community condemnation: M=1.84, SD=1.05 - Self-judgment: M=2.00, SD=1.03 - Isolation: M=1.22, SD=0.81 | Factors significantly associated with lower stigma   - Total reproductive autonomy (ß= -0.43 (-0.52 to -0.34), p < .001) - Decision Making:   - Worries about judgment (β = –0.20)   - Self-judgment (β = –0.25) - Communication with partner:   - Isolation (β = –0.26)   - Worries about judgment (β = –0.22)   - Self-judgment (β = –0.27)   - Community condemnation (β = –0.33) |
| **O’Donnell et al.** (2018)**^2^**  Country: IRL  Sample: n=155 | **ILAS scale**   - score range: 0-4 - Subscales: - Worries about judgment, α = 0.91 - Community condemnation, α = 0.78 - Self-judgment, α = 0.86 - Isolation, α = 0.83 | **Subscales:**   - Worries about judgment: M=1.74, SD=0.92 - Community condemnation: M=2.39, SD=1.13 - Self-judgment: M=1.54, SD= 0.96 - Isolation: M=1.27, SD=089 | Predicting psychological distress   - Anticipated stigma (ß = 0.08, p= 0.37) - Perceived stigma (ß = -0.06, p= 0.50) - Internalized stigma (ß = 0.41, p= <0.001) - Stigma-related isolation (ß = 0.23, p= 0.004)   **Predicting somatic symptoms**   - Anticipated stigma (ß = 0.08, p= 0.39) - Perceived stigma (ß = -0.04, p= 0.66) - Internalized stigma (ß = 0.42, p= <0.001) - Stigma-related isolation (ß = 0.16, p= 0.061)   **Mediation Analyses**   - Internalized stigma on somatic symptoms: (R2 = 0.21) - Stigma-related isolation on somatic symptoms: (R2 = 0.09) |
| **Sonalkar et al.** (2017)**^3^**  Country: USA  Sample: n=46 | **ILAS scale**   - score range: 0-4 - Subscales: - Worries about judgment - Community condemnation - Self-judgment - Isolation | **Total experienced stigma**  M=1.02, SD=0.60 | **Total experienced abortion stigma among** (unadjusted)   - Unexposed group: Median=1.25, IQR= 0.7–1.9 - Exposed group: Median=0.75, IQR 0.5–1.05 - Significant difference between exposed vs. unexposed group p=0.016   **Factors significantly associated with *higher* stigma**   - Participants with low income (≤ $30,000) or unsure about income had higher ILAS scores (p=0.61) |
| **Steinberg et al.** (2016)**^3^**  Country: USA  Sample: n=353 | Single-item indicator for perceived stigma:   - Believe will be looked down on by others for having abortion - score range: 0-4 | **Perceived abortion stigma**  Prevalence: 37.4 % (n=132)  M=2.89 – 2.92 | Perceived abortion stigma was a strong predictor of worse pre-abortion mental health:   - Depressive Symptoms (r =.43, p<0.0005), - Anxiety (r =.39, p < 0.0005), - Stress symptoms (r =.40, p < 0.0005) - Perceived abortion stigma was associated with pre-abortion depressive symptoms (β = 3.30, SE = 0.41, p < .0005) |
