# Supplementary material for: Abortion stigma among people seeking abortion care in high-income countries: a mixed-methods systematic review
Source: Sex Reprod Health Matters. 2026 Jul 27;33(1):2709964. doi: 10.1080/26410397.2026.2709964 (PMC13660548; doi:10.1080/26410397.2026.2709964)
Supplement: Supplementary file 5 [file ZRHM_A_2709964_SM9491.docx]

Supplementary Table 5. Main characteristics of the included studies

| **Study** | **Description of main results** | **Methodology** | **Goal of the study/ Phenomena of interest** | **Methods for data collection and analysis (QUAL only)** | **Country** | **Participant characteristics and sample size** |
| --- | --- | --- | --- | --- | --- | --- |
| **Quantitative Studies** | | | | | | |
| Biggs et al. (2020) | The study found that abortion stigma is widespread and can lead to psychological distress, particularly among those who were denied an abortion and later had a miscarriage or an abortion elsewhere. The effects of stigma can persist for up to two years but may diminish over time as individuals become more accepting or engage in experiences that reduce perceptions of stigma. | Quantitative Approach | The study aimed to prospectively assess perceptions of abortion stigma after receiving or being denied an abortion over 5 years, the factors associated with perceived abortion stigma, and the effects of perceived abortion stigma on psychological well-being. | **/** | USA | Sample size: 928 women  Age range: 14-46 Time since abortion: Seeking abortion |
| Chesnokova et al. (2024) | The study found that trust in an abortion provider and cohabitation were associated with lower abortion stigma, while anxiety and depression were associated with higher stigma. Other factors such as age, race, income, BMI, parity, gestational age, and reasons for ending the pregnancy were not significantly associated with stigma. | Quantitative Approach | To determine whether trust in the provider and sociodemographics are associated with individual-level abortion stigma. | **/** | USA, Pensilvenia | Sample size: 70 participants* Age range: <25->=35 Gestational age: second trimester abortions |
| Hanschmidt et al. (2018) | Women with higher gestational age and those who did not perceive their fetus to have a low chance of survival reported higher levels of stigma, while higher perceived partner support was associated with lower levels of stigma. Women who have abortions after a diagnosis of fetal abnormality experience less stigma than those who have abortions for socioeconomic reasons. | Quantitative Approach | The aim of this study was to investigate the psychometric properties of the ILAS scale in a German sample of women who had had an abortion after diagnosis of fetal anomaly. We further sought to explore socioeconomic and abortion-related factors associated with women‘s experience of stigma. | **/** | Germany | Sample size: 148 women  Age: at least 18 years old at assessment Gestational age: abortions after fetal anomaly |
| Hanschmidt et al. (2017) | The study found that internalized stigma is associated with long-term psychological distress following TOPFA, and that perceived stigma at the time of TOPFA may contribute to increased trauma and grief symptoms. Women's experience of stigma was associated with increased psychological distress. However, the cross-sectional design limits causal interpretation of the findings. | Quantitative Approach | The second aim of the study was to explore the association among perceived stigma, internalized stigma, and women’s psychological health following a TOPFA. | **/** | Germany | Sample size: 148 women  Age: at least 18 years old at assessment Gestational age: abortions after fetal anomaly |
| Kerns et al. (2022) | Higher self-judgment at the time of abortion was significantly associated with increased postabortion grief, but not with posttraumatic stress or mental health. Perceived community judgment was not associated with psychological outcomes. Abortion stigma contributes to psychological distress, but there was no significant association between stigma and mental health. Self-judgment as a predictor of post-traumatic stress was sensitive to trait anxiety. | Quantitative Approach | The study aimed to examine how retrospective measures of self-judgment and perception of community condemnation at the time of abortion impact postabortion grief, post-traumatic stress, and overall mental health in women who had an abortion for maternal or fetal complications in the second trimester. | **/** | USA | Sample size: 353 women  Age: 18 years and older Time since abortion: Who had an abortion within 5 years of survey completion. |
| Metha et al. (2019) | The study found that higher levels of reproductive autonomy were associated with lower levels of abortion stigma among women who had abortions. Specifically, the decision-making and communication components of reproductive autonomy were negatively associated with abortion stigma. This suggests that a woman's level of power and control in her intimate relationship is related to the stigma she experiences with abortion. | Quantitative Approach | This is the first study, to our knowledge, to evaluate the relationship between reproductive autonomy and abortion stigma among U.S. women who have ever had an abortion. | **/** | USA | Sample size: 587 women Age range: 15-60 Time since abortion: Women who had ever had an abortion. |
| O’Donnell et al. (2018) | The study found that internalized stigma and stigma-related isolation were predictors of higher levels of psychological distress and somatic symptoms among women who had abortions. The effects were driven by the contributions of internalized stigma and stigma-related isolation rather than anticipated or perceived stigma. The findings suggest that internalized shame and intentional isolation from others are associated with impaired well-being. | Quantitative Approach | The study aims to advance existing knowledge by investigating the link between abortion stigma and psychological distress and symptoms of poor physical health. | **/** | Republic of Ireland | Sample size: 155 women  Age range: 18-68 Time since abortion: The time since their last abortion varied from 12 months to 47 years ago. |
| Sonalkar et al. (2017) | Exposure to mandated abortion counseling had some effect on stigma scores, but this effect was confounded by income category and the results were not statistically significant. However, the pilot study suggests that further research is needed to fully understand the impact of mandated counseling on patient stigma. | Quantitative Approach | The study aimed to investigate the effect of mandated abortion counseling requirements on patients' individual-level abortion stigma. | **/** | USA, Pennsylvania | Sample size: 23 women per group  Age range: 18 - <40 Time since abortion: English-speaking women seeking their first induced abortion. |
| Steinberg et al. (2016) | Perceived abortion stigma was strongly associated with negative pre-abortion mental health symptoms and explained the most additional variance in depressive, anxiety, and stress symptoms compared with other predictors. | Quantitative Approach | The study aimed to examine which, of a variety of psychosocial factors—childhood adversities, adverse experience with an intimate partner, relationship context, pregnancy desires, and perceived abortion stigma—were independently associated with pre-abortion mental health. | **/** | USA | Sample size: 353 women Age: 18 or older Time since abortion: Seeking abortion service |
| **Qualitative studies** | | | | | | |
| Aiken et al. (2017) | Over a 4-month period, 519 women sought medication abortion from WoW. 49% of the 209 reasons given for seeking abortion outside formal healthcare settings were access barriers, 30% were privacy concerns, and 18% were controlling circumstances. | Descriptive Qualitative | Examine the reasons why women in Britain might look for other avenues to abortion care outside the formal healthcare system and explore: 1) any barriers women report to accessing services; and 2) their perspectives on self-sourcing their own medication abortions to carry them out at home. | WoW online consultation forms (emails sent to the helpdesk). Content was analyzed by two researchers independently and coded according to an "iteratively developed coding guide" (Aiken2018, p. 3). A summative content analysis approach was used in group discussions to resolve differences. | Great Britain | 180 women 77% reported a gestational age of fewer than 7 weeks, with the remaining 23% reporting between 7- and 10-week’s gestation Mean age 28 years |
| Aiken et al. (2018) | The study identified four key themes: 1) preference for self-managing medication abortion at home using telemedicine, 2) lack of pre- and post-abortion support in the Irish healthcare system, 3) desperation leading to dangerous methods, and 4) impacts of Irish abortion law and attitudes on shame and stigma. | Descriptive Qualitative | 1) to examine the factors affecting whether women in Ireland choose to access abortion by travelling or by using online telemedicine; and 2) to explore their experiences in accessing care through each pathway. | Semi-structured in-depth interviews were conducted. Detailed field notes were recorded and discussed in the research team. Interviews were described and coded by two team members. Principles of the Grounded Theory was used for analysis. | Great Britain | 38 women who had an abortion within the last 8 years Age range: 18 to 42 years |
| Altshuler et al. (2017) | Based on interviews with twenty women, three themes emerged: to be affirmed as moral decision-makers, to be able to determine their degree of awareness during the abortion, and to have care provided in a discreet manner to avoid being judged by others for having an abortion | Grounded Theory | Examine ways in which women's needs and preferences in abortion care differ from intrapartum care. | Interviews and field observations of abortion and birth facilities. The interviews were professionally transcribed verbatim. Codes were developed from Lyerly's birth framework and emerged from the collected data. Data was first categorized using Lyerly's birth framework and then further analyzed using grounded theory. | USA, Northern California | 20 women who had an abortion in the last 5 years  Experiences of 34 induced abortions 31 births and 6 miscarriages  Inclusion age 18 to 49 years |
| Beynon-Jones (2017) | The article discusses the differences between discourse analysis and research on abortion stigma. It suggests that research on abortion stigma can reify it by not considering how identities are renegotiated through language use. Discursive psychology emphasizes the speaker's capacity to construct non-stigmatized identities while acknowledging the constraints of the context. | Discursive psychology | Drawing on insights from discursive psychology [...] to explore how women in England talk about having had an abortion during research interviews. | Telephone interviews were analyzed using a discourse analysis. | Great Britain | 28 women Time since abortion was three weeks to 37 years |
| Broussard (2020) | The study explores the experiences of women seeking abortion in Northern Ireland and the Republic of Ireland, where social stigma and restrictive laws were major barriers to care. Women either traveled abroad for clinical care or self-managed a medication abortion at home. Structural stigma shaped perceptions of pain, the fetus, and the environment in which care was sought. Medication self-management allowed women to redefine their beliefs about pregnancy and gain greater experiential knowledge. Preferences and attitudes about the environment of care were informed by stigma and perceptions of risk. | (De)medicalization theory, descriptive qualitative, an "integrated understanding of the relationship between the biological and the social” (Bendelow, 2000). | Describe the ways structural stigma influences women’s embodied experience of abortion at three levels of analysis. | Semi-structured in-depth interviews were conducted and detailed field notes taken. Audios were transcribed and analyzed according to the principles of grounded theory. | Republic of Ireland and Northern Ireland | 68 women who had an abortion in the last 8 years  Age range: 18–44 years 27 were mothers Gestational age: Majority of participants in sample terminated a pregnancy that would be classified as an ‘embryo’ due to a gestational age of less than 11 weeks, |
| Broussard (2024) | The findings suggest that disclosure can be strategically used by people with stigmatized needs to confront and evade stigma and surveillance from their networks, the state, and the formal health care system. | Network analysis | The study examines how women navigate their social networks to seek information and support for ending unwanted pregnancies. It explores the use of strategic (non)disclosure and partial disclosure as a means of resistance, autonomy, and protection for marginalized populations. | The author conducted in-depth interviews. She used a flexible approach to coding and analyzing the interviews, transcribing four interviews to develop a coding guide and using broad codes to reflect the research concepts and questions. | USA | 55 women who attempted a self-managed abortion within the past 10 years  Age range: 14-49 years |
| Carroll et al. (2022) | Most participants expected to see protesters at their visits but were surprised at the number present and how close they were able to get to patients, which made some feel unsafe. Participants also reported that protesters' offers of adoption and financial support were unwanted. Participants credited clinic escorts and security with helping mitigate negative protester encounters. Nearly all felt the city ordinance would have improved their experience by making them feel safer and more at ease. | Descriptive Qualitative | People’s experience seeking abortion care who had obtained services before the ordinance was passed when protesters were a daily presence outside the clinic. | Interviews were transcribed and analyzed with inductive and deductive approaches for content and themes. | USA, Mississippi | 30 participants* were recruited two to six months after a consultation visit Age range: 18 to >35 |
| Cashman et al. (2021) | Accessing MToP through a sexual health service was considered positive, non-judgmental, discrete, and low-cost despite challenges of fitting in with appointment times and obtaining off-site ultrasound. GPs did not always provide referral; some women described experiences of stigma, discrimination, and judgmental care during consultation and when obtaining ultrasounds | Descriptive Qualitative | This study explores experiences of women accessing Medical termination of pregnancy through a regional sexual health service. | The interviews were followed by an iterative analysis of data, where the researcher met after each interview for debriefing. This was followed by an "in-depth thematic analysis  guided by Braun and Clarke (Cashman et al. 2021, p. 3)" after the completed data collection. | Australia, Queensland | 11 women 4–6 weeks after the abortion, ten had medical pregancy termination, one woman had a surgical termination Age range: 23-42 years, mean: 32 years |
| Chandrasekaran et al. (2023) | The paper explores how cultural and community views affect medication abortion access and experiences among Asian-Americans, filling gaps in research. | Descriptive Qualitative | Religion negatively affects the acceptance of abortion in families and communities. Lack of openness about sexual and reproductive health leads to stigma and isolation for those who have had abortions. | The study used a community-based participatory research approach to understand abortion knowledge, attitudes, and experiences among Asian-Americans. Semi-structured interviews were conducted via phone or Zoom, and were audio recorded and transcribed. Trained researchers used an iterative coding process to analyze the data. Ethnic identities were described using the language participants used to describe themselves. | USA | 29 Asian American or mixed race including Asian American, Native Hawaiian, and/or Pacific Islander (AANHPI)* who had a medication abortion between January 2016 and March 2021 Age range: 20-44 |
| Chor et al. (2019) | Women were selective about whom they spoke to regarding their abortion decision. Reasons to discuss included seeking information, guidance, and support. Reasons not to discuss included concerns about judgment and privacy. Most women who spoke about their decision experienced a positive response. | Descriptive Qualitative | The nature of the conversations that women have prior to obtaining an abortion, to understand who women turn to within their social network as sources of support, whom they regard as sources of stigma in abortion decision-making, and how these conversations influence women’s feelings about their abortion decision. | Semi-structured interviews were conducted and analyzed using a "modified template approach" (Chor et al. 2019, p. 3). Interrater reliability of five transcripts was 84.5%. | USA, Chicago | 30 women Age range: 18 to >36 Gestational age: ≤ 13 6/7 weeks gestation |
| Dickey et al. (2022) | Social support and economic support are important for both abortion access and parenting, but poverty, unstable partnerships, structural inequality, and abortion stigma can create barriers to accessing economic support. Women may feel constrained in their reproductive autonomy and have no alternative but abortion. | Descriptive qualitative, theoretical framework: Social support and social capital | Explore the dynamics of social support and social capital within people’s abortion-seeking experiences by describing the roles of partners, family, and friends in navigating abortion decision-making, identifying how to obtain abortion care, paying for and accessing care, and dealing with the emotional experiences of unintended pregnancy and abortion. | Semi-structured, in-depth interviews were conducted to collect the data. The transcripts were analyzed using "Sort, Sift, Think, Shift protocol (Maietta et al., 2021), utilizing a combination of approaches from traditional qualitative methods, including grounded theory, narrative analysis, and phenomenology". | USA, Georgia | 18 cisgender women were recruited on the day of their procedure Mean age: 28.78 Time of interview: shortly after a six-week gestational age |
| Doran and Hornibrook (2016) | Five themes were identified: finding information about the provider; stigma, shame and secrecy; logistics involved in accessing the clinic related to travel, money and support; medical and surgical abortion; and ways rural women could be better supported in this process | Descriptive Qualitative | Identify factors that impact the experience of rural New South Wales women in accessing abortions and suggestions about how rural women could be better supported when seeking an abortion. | In-depth interviews were conducted and analyzed thematically. | Australia, New South Wales | 13 women who had an abortion in the previous 5 months to 15 years prior to interview  Age range: 18 years to 46 years, mean age of 27.5 years |
| Foster et al. (2020) | The study found that interactions with protesters outside abortion clinics in certain Canadian provinces were unsettling, stigmatizing, and frustrating for participants. These encounters were especially upsetting for those who struggled with the decision to have an abortion or made the decision in the context of health issues or violence. Participants wished that the protesters had not been there and that their experiences accessing abortion care were more like accessing other types of healthcare. | Descriptive Qualitative | Aimed to understand better Canadian abortion patients’ experiences with protesters. | The researchers conducted semi-structured interviews and analyzed the transcripts with women who encountered protesters, notes and memos of content and themes. | Canada | 305 women who had an abortion in the previous five years Age range: 18–25 and 26 and above |
| Frohwirth et al. (2018) | In-depth interviews conducted with 78 women having abortions at nine sites in the United States found religion to permeate abortion stigma manifestations and management strategies identified in previous research, for religious and religiously affiliated respondents as well as those who did not claim a religious affiliation. | Descriptive Qualitative | Explore how religion influences religiously affiliated, and non-religiously affiliated women’s abortion decision making and experience. | Semi-structured in-depth interviews and analyzed inductively and deductively using a systematic analytical approach. | USA, Texas | 78 women were recruited on the day of abortion or approx. 2 weeks later Recruitment age: 18 years or older |
| Gelman et al. (2017) | Women face antiabortion attitudes from partners, family members, and themselves, leading to negative emotions and concealment or delay of abortions. These attitudes communicate that abortion is morally wrong, a rejection of motherhood, rare and potentially deviant, detrimental to future fertility, and an irresponsible choice. Women distinguish themselves from other women who obtain abortions. | Descriptive Qualitative | A more thorough understanding of the sources and range of attitudes that could reflect or contribute to abortion stigma among low-income women and their social networks is still needed to help inform strategies designed to improve women’s abortion experiences. | The researchers conducted semi-structured interviews. They analyzed the transcripts using content analysis. | USA, Western Pennsylvania | 19 women Age range: 18-24 Time since abortion: Planning to terminate their pregnancy (n=10), Had an abortion two weeks before (n=9) Gestational age: majority of women either had had an abortion in the first trimester or were in their first trimester of pregnancy |
| Grindlay et al. (2017) | Women who visited military treatment facilities during pregnancy felt upset and abandoned due to the lack of options counseling and referral. The military's abortion policy had negative consequences for servicewomen's health, emotions, finances, and logistics. Most women did not have accurate knowledge of the abortion policy. | Descriptive Qualitative | Better understand servicewomen’s abortion experiences, and their knowledge and beliefs about the military’s abortion policy. | In-depth interviews were conducted and analyzed inductively and deductively using thematic analysis. | USA | 21 women who had an abortion in the prior two years Age range: 18 to >30 |
| Hatcher et al. (2018) | Participants were open to discussing abortion services during women's wellness exams or contraception visits. Appropriate communication included using sensitive language, respecting patient beliefs, and contextualizing abortion services within reproductive health. Discussing available abortion services can strengthen the patient-provider relationship and reduce stigma surrounding abortion care. Routine inclusion of abortion services counseling may help educate patients and improve awareness of available services. | Descriptive Qualitative | Explore qualitatively when and how to best inform patients of available abortion services in a primary care setting. | The In-depth interviews were followed by iterative and collaborative process to identify emerging patterns and themes. | Not specified | 21 women Age range: 19-24 years, one participant was less than 18 years |
| Heller et al. (2016) | Four themes emerged relating to barriers to access and experience: (1) the impact of travel for TOP, (2) temporal factors unique to this population and how they affected women, (3) the attitude of health professionals, notably general practitioners, because of local culture, and (4) stigma surrounding TOP and the expectation that abortion will be traumatizing. | Descriptive Qualitative | Explore women’s perceptions of their pathway to TOP and to examine any impact of delays in obtaining treatment. | The individual semi-structured interviews were analyzed thematically. | Great Britain, Scotland | 16 women who had an abortion 5 weeks to 6 months (approx. 8 weeks after) after the abortion prior to the interview Age range: 18-41 years Gestational age at scan: 7-13 weeks |
| Hermannsdóttir (2022) | The author discusses women's silent maneuverings in relation to belonging and power, proposing the concept of performed belonging as a navigational strategy. | Anthropology, ethnography, theoretical framework of “choice as belonging” (Gammeltoft 2014) | To contribute to the theoretical discussion of abortion silence manifested in women’s lived experiences. | Individual narrative interviews, participant-observations (informal conversations), participation in mother's group and development of sexual health education programme for all first-year high school students, participation and following informal debates on social media. The interviews were analyzed using a combination of narrative analysis and the non-narrated elements of the interview (e.g. Issues or feelings). | Faroe Islands (Danish Kingdom) | 19 women (13 interviews involved abortion narratives) Age range: 19-67 years |
| Hoggart et al. (2017) | The study found that women's contraceptive histories and reproductive lives are complex, making it difficult to establish causal patterns for multiple abortions. Women who had multiple abortions expressed intensified abortion shame. | Descriptive Qualitative | Discussing young women’s experiences of seeking fertility control following an abortion; and assessing whether categorizing women who have abortions depending on number may be overly simplistic and unnecessarily stigmatizing. | In-depth qualitative interviews were conducted and analyzed using thematic and case-based approaches. | Great Britain, England and Wales | 36 women  Age range: 16-24 years |
| Hoggart (2017) | Participants in the study felt stigmatized by their abortions, but many resisted stigma through different strategies based on their socio-economic situation, family and relationship situations, circumstances of pregnancy, and beliefs/values. Constructing their abortion decision as morally sound was important for stigma resistance. | Critical realism, theoretical framework: abortion-related stigma | How individual women’s views on abortion, their abortion decision-making, and their feelings about their own abortion, are articulated. | The secondary qualitative analysis was based on "two qualitative research projects on young women’s experiences of abortion" (Hoggart et al. 2017, p. 4). A thematic top-down analysis, adopted from Braun and Clarke's approach, was used to analyze the data. | Great Britain, England and Wales | 46 women Age range: 16-24 years |
| Holten et al. (2021) | The main results show that women in the Netherlands lack autonomy in accessing abortion care due to institutionalized taboo, complex regulations, lack of permeability for marginalized groups, and inability to speak openly. To increase autonomy, legislators and policymakers must trust women, avoid stigmatizing abortion, and develop systemic support for vulnerable groups. | Grounded theory method, constructivist medical anthropological approach, reproductive justice theoretical framework | Women’s experiences with (online) abortion services and relevant HPs’ experiences delivering care, to identify key barriers for women seeking an abortion in the Netherlands | Semi-structured in-depth interviews were conducted and analyzed "using an abbreviated grounded theory method". In addition, WoW abortion-seekers’ Emails were deductively coded using the codes that emerged from the interviews. | Netherlands | 200 emails of women seeking abortion care, 20 women & 14 health professionals  Age range: 15-35 years |
| Holten et al. (2023) | The aim of this study was to portray the experiences of individual abortion-seekers in the Netherlands. The research question for this study was: What experiences do abortion seekers in the Netherlands have with abortion care and what new insights about these experiences can the I-poem method of analysis provide? | Constructivist feminist approach Reproductive justice approach | Abortion-seekers face obstacles in policy and care, causing fear and anxiety. Autonomous choice in abortion care is socially constructed. Providers must consider external factors like partner discordance and waiting times. Normalization of information is necessary for informed choice and reduction of stigma. | The I-poem method was used to analyze interview data. The method involved creating poems from interviewees' statements using the first person "I". The poems were analyzed using abbreviated grounded theory to gain new insights and counteract stereotypes and stigma surrounding abortion. The I-poems were coded inductively and deductively to identify themes and validate previous research findings. | Netherlands | 101 I-poems from 20 interviews*  Age range: 19-35 Gestational age: Before 12 weeks |
| Hutchens (2023) | To analyze the experience at two pregnancy centers, with particular attention to the ways in which gender, race, and class shape their experiences within and perceptions of pregnancy centers. | Descriptive Qualitative,  Intersectional framework | Clients prefer pregnancy centers over clinical health care providers due to the emotional care they receive, which is important in maintaining the centers' legitimacy. These evaluations are influenced by clients' reproductive histories, which are shaped by gender, racism, and economic inequalities. | The data was analyzed inductively, organizing it by category and source, and developing open and focused codes to identify themes and connections. | USA | 800 hours of fieldwork (off-site events & 68 appointments) and 29 interviewees (28 Female/women; 1 Gender fluid) |
| Kavanaugh et al. (2019) | The study found two logistical information-related barriers: 1) the general lack of reproductive-related knowledge and unhelpfulness on the part of perceived members of the healthcare community and 2) one broader barrier of perceived stigma within respondents’ narratives. | Descriptive Qualitative | Identify barriers to accessing information about abortion among individuals who traveled out of state or substantial distances and presented for care at an abortion facility, and to identify shared characteristics and/or experiences among abortion patients who did not encounter these barriers. | In-depth interviews conducted and analyzed for themes using an inital coding scheme, which was updated throughout the coding process. | USA, Michigan and New Mexico | 29 women  Majority age: 20s Gestational age: 0-7 to 20-25 weeks |
| Kerestes et al. (2022) | Participants perceived abortion stigma which often led them to avoid traditional  care and experienced enacted stigma during encounters with non-study healthcare  workers. | Descriptive Qualitative, theoretical framework: The person-centered framework for reproductive health  equity (PCFRHE) | Aim to show that the experience of TelAbortion is person-centered care and can be a model for other direct-to-patient telemedicine abortion care. | The researchers conducted semi-structured telephone interviews and met throughout the data collections process to discuss transcripts and interview process. The transcripts were analyzed using qualitative content analysis. | USA, New York, Georgia, Illinois, Minnesota, Colorado, Hawai’i, and Oregon | 45 participants* who had an TelAbortion in the previous six months Median age: 29 Gestational age: 8 (4–10) |
| Kerestes et al. (2021) | Stigma led to a prioritization of privacy, and by going to a clinic, participants feared a loss of privacy whereas obtaining a medication abortion by mail made it easier to maintain confidentiality. | Descriptive Qualitative | Explored how this method impacted participants’ ability to access abortion care outside of the Risk Evaluation and Mitigation Strategy restrictions and what their alternatives would have been if they had not been able to find this care. | Semi-structured interviews were conducted and analyses using a qualitative content analysis. | USA, New York, Georgia, Illinois, Minnesota, Colorado, Hawai’i, and Oregon | 45 participants* who had an TelAbortion in the previous six months Median age: 29 Gestational age:8 (4–10) |
| Killinger et al. (2022) | The study identified two main themes: internal motivations for seeking telemedicine abortion (autonomy, perception of external threat, shame, and stigma) and external barriers to formal abortion care (financial stress, logistic barriers to access, provider attitudes, and vulnerability of foreigners). | Descriptive Qualitative | Understand the motivations, and perceived barriers to access, for women in Germany choosing telemedicine abortion over formal services | During a mixed methods study, responses to email telling the women „That abortion is available in the formal healthcare sector followed by this question: If you feel you are unable to access abortion services in Germany, can you please tell us a bit more about  why. […] We will let you know as soon as possible if we can help you in any way." (Killinger et al. 2020, p. 2). These responses were analyzed using a content analysis. | Germany | 255 emails of women following online consultations from Germany |
| Kimport (2019) | The research found two primary ways CPC counselors stigmatized abortion and describe variation in their impact: counselors’ efforts were “successful,” were misrecognized as ideologically neutral, or were resisted. | Descriptive Qualitative | Explore Crisis pregnancy centers (CPCs)—antiabortion organizations that offer one-on-one “prolife counseling”— for how and when interactions matter for abortion stigmatization. | The research team conducted in-depth interviews. Elaborative coding was used to analyze in-depth interviews related to visiting a CPC and abortion stigma. | USA, Louisiana and Baltimore | 25 women Age range: 20-43 years Gestational age: after 24 weeks of gestation |
| Kimport & Rasidjan (2023) | The main results show that traveling for abortion care causes emotional burden, distress, stress, anxiety, and shame for interviewees. It also leads to disclosure of abortion to others, obtaining care in an unfamiliar place, and feeling excluded due to the denial of in-state abortion care. | Descriptive Qualitative | Examine the emotional costs abortion travel exacts and the mechanisms underlying them. | Semi-structured interviews were conducted. Excerpts related to travel were exported and reviewed by the authors. The remaining excerpts were sub coded using grounded theory techniques, with emotional effects as a sensitizing concept. | USA | 30 cisgender women Age range: 18-43 years Gestational age: after 24 weeks gestation |
| LaRoche et al. (2021) | Participants who had abortions in criminalized settings experienced negative emotional impacts and felt they had to fit their story into a state-mandated narrative. They also feared being denied care if they were honest with clinicians. Participants supported decriminalization of abortion and consistent legal status across Australia. | Descriptive Qualitative | Explore differences in the experiences of patients who accessed care in both criminalized and decriminalized settings across Australia. And to document participants’ thoughts about these policies | The conducted in-depth interviews and reviewed data as they were collected to identify common themes and establish thematic saturation. They performed content and thematic analyses of the interactions using both predetermined codes and inductive analysis techniques. | Australia | 22 women, transgender and gender non-binary people Age range: 19-45 years |
| LaRoche et al. (2020) | The study found that participants encountered variable and often significant financial costs when obtaining their abortion. Many expressed that their interactions with general practitioners when trying to locate an abortion provider were uninformative and stigmatizing. | Descriptive Qualitative | To explore the experiences of Australian abortion patients obtaining mifepristone through different service delivery models in different geographic areas. | The researchers used in-depth interviews to collect data. They reviewed data as they collected them and identified common themes. Content and thematic analyses were conducted using predetermined codes and inductive analysis techniques. | Australia | 22 participants (women, transgender and gender non-binary people) Age range: 19-45 years |
| LaRoche & Foster (2018) | Women described intensified feelings of shame and both internalized and externalized stigma surrounding their decision to have more than one abortion. | Descriptive Qualitative | Document women’s lived experiences and the interplay between multiple abortions and abortion-related stigma. | The study used semi structured in-depth telephone/Skype interviews for data collection. Data was reviewed and common themes were identified as data was collected. Content and thematic analyses were conducted using priori codes and inductive analysis techniques. | Canada | 41 participants who had obtained at least one abortion in the 5 years preceding the interview Age range: 19-43 years, mean age: 26.7 years |
| Lerma et al. (2023) | The study explores Mississippi abortion clients' interest in alternative medication abortion service delivery options that are restricted under state law but available elsewhere, including telemedicine, medications by mail, and flexible follow-up. | Descriptive Qualitative | Participants had mixed opinions about using telemedicine for abortion, with some concerned about privacy and postal errors. Those who did not use alternative options cited concerns about missing out on in-person care. Many did not return for follow-up care because of financial and logistical barriers and were not interested in receiving care in their community because of concerns about provider judgment, stigma, and privacy. | The study used in-depth interviews and thematic analysis was used to organize codes into common themes. A codebook was developed. The themes related to the advantages and disadvantages of remote medication abortion models and alternative follow-up options. | USA, Mississippi | 25 participants* who had a medication and procedural abortions Age range: 21-38 years |
| Mahanaimy et al. (2022) | Regarding abortion, participants described an unmet need for accurate information and frequent stigmatizing experiences with dismissive and judgmental care providers. | Descriptive Qualitative | Young people’s experiences obtaining healthcare during an unintended pregnancy at or before age 25. | In-depth interviews were conducted, and thematic analysis was used to organize responses across interviews. | USA, California | 25 women who had at least one unintended pregnancy when they were 25 years old or younger Age range: 18-30 years |
| Makleff et al. (2023) | The study aims to understand the impact of abortion stigma on the quality of care and the experience of abortion seekers. It identifies the need to address stigma at multiple levels to achieve equitable access to quality abortion care. | Phenomenological qualitative study  Theoretical Framework: Person-centered care for reproductive health equity | The study developed typologies of stigmatizing care and identified macro-level factors contributing to poor experiences in care related to abortion. Positive experiences were linked to patient-centered care and a lack of stigma. The study found that decriminalization of abortion had a positive impact on access to care | The study collected data through in-depth interviews with abortion seekers in Australia. Thematic analysis was used to identify typologies of interactions between abortion seekers and health care workers, focusing on stigma and person-centered care. | Australia | 24 women with medication and surgical abortions  Age range: 20-40 years Gestational age: 5 - 28 weeks |
| Margo et al. (2016) | Women reported experiencing emotional strain, stress, and stigma, and described the value of receiving social support. | Descriptive Qualitative | Women’s experiences with health professionals prior to seeking abortion; whether and how abortion referrals are offered; and how women seek abortion care without professional referrals. Study with women who successfully obtained abortion care Pathways to Abortion Care in South Carolina in South Carolina. | Semi-structured qualitative interviews were conducted. A codebook was developed, and inter-coder reliability was evaluated. Thematic analysis approach was used with domains specified a priori and unexpected themes allowed to emerge. | USA, South Carolina | 45 women were recruited on day of abortion on-site Age range: 18-35 |
| Monchalin et al. (2023) | The paper reports on preliminary findings from an exploratory study on access to and experiences with abortion services among Indigenous Peoples in Canada. The study aims to advance knowledge on the topic and is Indigenous led. The research question being explored is "What shapes Indigenous experiences of accessing abortion care in Canada?” | Indigenous feminism  Exploratory qualitative study with an indigenous methodology | Indigenous Peoples in Canada face unique challenges in accessing abortion due to logistical barriers, poor treatment, stigma, colonialism, and lack of traditional knowledge and follow-up care. These barriers are closely linked to the impacts of colonialism. | The study utilized conversational interviews with an Indigenous Advisory Committee to explore abortion service access and support in Canada. Data analysis followed a six-stage DEPICT model, including dynamic reading of transcripts, codebook development, participatory coding, reviewing and summarizing categories, collaborative analyzing, and translation. The research team and Advisory Committee reviewed themes and revisited project objectives to develop a knowledge translation strategy. | Canada | 15 indigenous people* who had a procedural abortion between 5 and 15 years prior to their interview Age range: 16-29 |
| Purcell et al. (2020) | Drawing on qualitative secondary analysis of five UK datasets using a narrative inquiry approach, the study identified: the use of non-negative language around abortion, potential components of a normalizing narrative, and constraints on non-negativity | Phenomenological-sociological perspective and qualitative secondary analysis | Exploring absences of stigma, instances in which participants potentially challenged or rejected stigma, by framing this as ‘non-negativity’. | Authors conducted a qualitative secondary analysis. A flexible and systematic approach was used to analyze data within and across datasets on non-negative talk about abortion. | Great Britain, Scotland and England | 25 women (sub-sample) |
| Rafferty & Longbons (2021) | Each site of struggle characterized a different noteworthy moment within a woman’s medication abortion experience: the decision, the medication abortion process, identity after abortion, and managing the stigmatizing silence before and after the abortion. | Case study, theoretical framework: Relational Dialectics Theory (RDT) | Explore the complexity and consequentiality of women’s language choices with anonymously telling their own medication abortion story, as well as offer the potential to capture the interplay of individual, organizational, and social discourses surrounding the abortion debate. | The research performed a case study of the nonpartisan website, Abortion Changes You. The case study approach was used to develop a thick description of the Abortion Changes You website. Baxter's contrapuntal analysis was used to study the meanings circulating around individual and relational identities evidenced within the language choices of the women blogging about their own medication abortion. Thematic analysis was conducted to identify the discourses evident within each blog post. | English website | 98 blogs from women who have had a medication abortion and shared their story on the website |
| Warren et al. (2022) | The researchers found enacted and anticipated stigmatization and document how women who are considering an abortion mitigate stigmatization through impression management and other responsive mechanisms. | Descriptive Qualitative | Examine how Crisis Pregnancy Centers (CPC) serve as sites where people seeking abortion care are directly stigmatized by the CPC as an institution and by CPC staff members as individuals. | The study used semi-structured qualitative interviews and field notes to collect data from CPCs. Two phases of coding were conducted in NVIVO, with initial analysis focusing on themes related to why clients choose CPCs and how staff understand their purpose. Secondary analysis revealed new themes related to stigmatization and health misinformation. | USA, Ohio | 22 women that visited a CPC Age range: 19-33 years |
| Wilson-Lowe (2024) | The article explores women's use of online spaces to explore abortion experiences and addresses three research questions: why women seek abortion-related content online, how they access it, and their experience of online anti-abortion rhetoric. | Descriptive qualitative | The study found that many participants sought online support for abortion but struggled to find relevant and supportive content due to stigma. Those who received guidance towards specific resources had positive experiences. Key themes included obtaining unavailable support, preparation for abortion, and reducing isolation. | The interviews were conducted remotely and analyzed using reflexive thematic analysis. The analysis generated common themes, which were refined and interpreted collaboratively by the authors. | Great Britain, Scotland | 23 cisgender women that sought an abortion (no time limit) Age range: 20-54 |
| Woodruff et al. (2020) | Many respondents experienced harassment and negative incidents after sharing their story publicly, leading to emotional stress and difficulties at work/school. However, positive experiences were also reported by the majority, motivating them to continue sharing their story. | Descriptive Qualitative | Understanding the experiences of people who have chosen to share their abortion stories publicly. | An Anonymous online survey used for data collection. The qualitative research strand focused on open-ended question responses categorized into themes based on survey items and emerging data. | USA | 88 people who have shared their personal abortion story in a public setting  Age: 18 years or older |

QUAL: Qualitative studies; *sex/gender not specific
