# Supplementary material for: Abortion stigma among people seeking abortion care in high-income countries: a mixed-methods systematic review
Source: Sex Reprod Health Matters. 2026 Jul 27;33(1):2709964. doi: 10.1080/26410397.2026.2709964 (PMC13660548; doi:10.1080/26410397.2026.2709964)
Supplement: Supplementary file 6 [file ZRHM_A_2709964_SM9492.docx]

Supplementary Table 4. Critical Appraisal of the qualitative studies

| **Citation** | **Q1** | **Q2** | **Q3** | **Q4** | **Q5** | **Q6** | **Q7** | **Q8** | **Q9** | **Q10** |
| --- | --- | --- | --- | --- | --- | --- | --- | --- | --- | --- |
| Aiken et al. (2018) | N | (Y) | Y | Y | Y | N | Y | Y | Y | Y |
| Aiken et al. (2017) | N | (Y) | Y | Y | Y | N | N | Y | Y | Y |
| Altshuler et al. (2017) | N | Y | Y | Y | Y | N | Y | Y | Y | Y |
| Beynon-Jones (2017) | Y | Y | Y | Y | Y | Y | Y | Y | Y | Y |
| Broussard (2020) | N | (Y) | Y | Y | Y | Y | Y | Y | Y | Y |
| Broussard (2024) | N | Y | Y | Y | Y | N | N | Y | U | Y |
| Carroll et al. (2022) | N | (Y) | Y | Y | Y | N | N | Y | Y | Y |
| Cashman et al. (2021) | N | (Y) | Y | Y | Y | N | U | Y | Y | Y |
| Chandrasekaran et al. (2023) | N | (Y) | Y | Y | Y | N | N | Y | Y | Y |
| Chor et al. (2019) | N | (Y) | Y | Y | Y | N | U | Y | Y | Y |
| Dickey (2022) | N | (Y) | Y | Y | Y | N | N | Y | Y | Y |
| Doran & Hornibrook (2016) | N | (Y) | Y | Y | Y | N | N | Y | Y | Y |
| Foster et al. (2020) | N | (Y) | Y | Y | Y | Y | N | Y | Y | Y |
| Frohwirth et al. (2018) | N | (Y) | Y | Y | Y | N | U | Y | Y | Y |
| Gelman et al. (2017) | N | (Y) | Y | Y | Y | N | U | Y | Y | Y |
| Grindlay et al. (2017) | N | (Y) | Y | Y | Y | N | U | Y | Y | Y |
| Hatcher et al. (2018) | N | (Y) | Y | Y | Y | N | N | Y | Y | Y |
| Heller et al. (2016) | N | (Y) | Y | Y | Y | N | N | Y | Y | Y |
| Hermannsdóttir (2022) | Y | Y | Y | Y | Y | Y | Y | Y | N | Y |
| Hoggart et al. (2017) | Y | Y | Y | Y | Y | Y | Y | Y | Y | Y |
| Hoggart (2017) | N | (Y) | Y | Y | Y | N | N | Y | Y | Y |
| Holten et al. (2021) | Y | Y | Y | Y | Y | N | U | Y | Y | Y |
| Holten et al. (2023) | Y | Y | Y | Y | Y | Y | Y | Y | Y | Y |
| Hutchens (2023) | Y | Y | Y | Y | Y | N | N | Y | Y | Y |
| Ireland et al. (2020) | N | Y | Y | Y | Y | N | N | N | Y | Y |
| Kavanaugh et al. (2019) | N | (Y) | Y | Y | Y | N | N | Y | Y | Y |
| Kerestes et al. (2021) | N | (Y) | Y | Y | Y | N | N | Y | Y | Y |
| Kerestes et al. (2022) | N | (Y) | Y | Y | Y | N | N | Y | Y | Y |
| Killinger et al. (2022) | N | (Y) | Y | Y | Y | N | N | Y | Y | Y |
| Kimport & Rasidjan (2023) | N | (Y) | Y | Y | Y | N | N | Y | Y | Y |
| Kimport (2019) | N | (Y) | Y | Y | Y | N | Y | Y | Y | Y |
| LaRoche & Foster (2018) | N | (Y) | Y | Y | Y | N | N | Y | Y | Y |
| LaRoche & Foster (2020) | N | (Y) | Y | Y | Y | Y | Y | Y | Y | Y |
| LaRoche & Foster (2021) | N | (Y) | Y | Y | Y | N | Y | Y | Y | Y |
| Lerma et al. (2023 | N | (Y) | Y | Y | Y | N | N | Y | Y | Y |
| Makleff et al. (2023) | N | Y | Y | Y | Y | N | N | Y | Y | Y |
| **Citation** | **Q1** | **Q2** | **Q3** | **Q4** | **Q5** | **Q6** | **Q7** | **Q8** | **Q9** | **Q10** |
| Mahanaimy et al. (2022) | N | (Y) | Y | Y | Y | N | N | Y | Y | Y |
| Margo et al. (2019) | N | (Y) | Y | Y | Y | N | N | Y | Y | Y |
| Monchalin et al. (2023) | Y | Y | Y | Y | Y | Y | Y | Y | Y | Y |
| Purcell et al. (2020) | N | Y | Y | Y | Y | N | N | Y | Y | Y |
| Rafferty & Longbons (2021) | N | Y | Y | Y | Y | N | Y | Y | NN | Y |
| Warren et al. (2022) | N | (Y) | Y | Y | Y | N | N | Y | Y | Y |
| Wilson-Lowe (2024) | N | (Y) | Y | Y | Y | N | N | Y | Y | Y |
| Woodruff et al. (2020) | N | (Y) | Y | Y | Y | N | N | Y | Y | Y |

Y= Yes, (Y) = Descriptive qualitative methodology assigned by reviewers, U = Unclear, NN= Not Needed, JBI critical appraisal checklist for qualitative research

**Note:** Nearly all studies did not state the philosophical perspectives that informed their methodology (Q1). In addition, most studies did not state their methodological approach (e.g., phenomenology) (Q2). We assigned a “descriptive qualitative methodology” to the studies that did not specify a methodology (marked as (Y)). We rated Q2 as N when assessing the ConQual score. Most studies lacked the authors' cultural and theoretical context (Q6) and did not disclose the influence of researchers on the research, or vice versa (Q7). We excluded this study from further analysis. All studies reported that their research was ethical (Q9), and their conclusions originated from the analysis and/or interpretation of the data (Q10).

**Questions:** Q1 = Is there congruity between the stated philosophical perspective and the research methodology?; Q2 = Is there congruity between the research methodology and research question or objectives?; Q3 = Is there congruity between the research methodology and methods used to collect data?; Q4 = Is there congruity between research methodology and the representation and analysis of data?; Q5 = Is there congruity between the research methodology and the interpretation of results?; Q6 = Is there a statement locating the researcher culturally or theoretically?; Q7 = Is the researcher’s influence on the research, and vice versa?; Q8 = Are participants, and their voices, adequately represented?; Q9 = Is the research ethical according to the current criteria or, for recent studies, is there evidence of ethical approval by an appropriate body?; Q10 = Do the conclusions drawn in the research report flow from the analysis or interpretation of data?
