# Supplementary material for: Abortion stigma among people seeking abortion care in high-income countries: a mixed-methods systematic review
Source: Sex Reprod Health Matters. 2026 Jul 27;33(1):2709964. doi: 10.1080/26410397.2026.2709964 (PMC13660548; doi:10.1080/26410397.2026.2709964)
Supplement: Supplementary file 7 [file ZRHM_A_2709964_SM9493.docx]

Supplementary Table 2. Synthesized qualitative findings organized by stigma category and thematic setting

| **Synthesized finding 1 – Experienced abortion stigma**  People seeking abortion care experienced abortion stigma through encounters and events involving healthcare providers, anti-abortion protesters, and members of their social networks. Participants described situations in which they were directly judged, pressured, dismissed, unsupported, harassed, or confronted with stigmatizing comments and behavior. These experiences included emotionally upsetting encounters with protesters, negative or distancing reactions from family members or others close to them, and interactions with healthcare providers who were experienced to be rude, judgmental, coercive, or unhelpful. In some cases, healthcare workers created barriers to care by withholding information, delaying referrals, denying support, or failing to respond to participants’ emotional and informational needs. Legal and structural conditions also became tangible in participants’ experiences when criminalization, restricted access, lack of clear information, or the need to travel shaped abortion care as isolating, burdensome, and morally marked. Together, these findings show that abortion stigma was not only experienced as a general social climate but was lived through specific interactions, institutional practices, and structural barriers that directly affected participants’ abortion experiences. | |
| --- | --- |
| **Findings** | **Categories** |
| Shift the conversation away and avoid the discomfort of a stigmatized choice. (U) | Direct social judgement and pressure |
| When clients disclosed their intentions, they were met with criticism and social distancing. (U) |  |
| Abortion was commonly framed as an immoral act and frequently depicted as “murder.” (social network) (U). |  |
| But then, external factors such as the partner’s/family’s wishes, stigma, health system restraints, legal factors, missing information, and necessary support intruded on their decision-making process and complicated it. (U) | Stigmatizing reactions from family and social networks |
| Family members had antiabortion views. (U) |  |
| Sofia's parents pleaded with her to allow them to adopt this potential child, as she recounted. (U) |  |
| Some who disclosed their decision to family or friends, however, received upsetting, negative responses. (U) |  |
| Faced stigmatization when they disclosed intentions to terminate their pregnancy. (U) |  |
| Experiences of enacted stigma were also explicitly linked with religion. (U) |  |
| Abortion specifically violated their own or their family members’ religious belief. (U) |  |
| Navigating antiabortion protests outside of clinics. (U) | Harassment and intimidation by anti-abortion protestors |
| Most participants said that they had to find street parking for at least one of their visits, and (as noted above) they frequently reported that protestors would follow patients’ cars down the road and then approach them as they started walking toward the clinic. (U) |  |
| Many participants described being ‘bombarded,’ ‘bum rushed’ and feeling overtaken by protestors, who were standing in the road and on the sidewalk leading to the parking lot as patients turned on to the street where the clinic is located. (U) |  |
| They overwhelmingly described protesters as “persistent,” “pushy and determined,” and “aggressive” as they made their way to and from the clinic entrance. (U) |  |
| Participants reported feeling unsafe in the presence of protestors, particularly when protestors surrounded the car and they could hear protestors yelling even though they were inside the vehicle. (U) |  |
| Some participants were also bothered to see children in the crowd of protestors. (U) |  |
| Found their encounters with protestors more emotionally difficult. (U) |  |
| Most protestors’ behaviors were not acceptable. (C) |  |
| External stigma was exacerbated by protestors. (C) |  |
| Many participants found encounters with protesters to be emotionally upsetting and stigmatizing. (U) |  |
| All protestors use a moralising discourse. (U) |  |
| Personal experience with an unplanned pregnancy during which she felt judged. (U) | Judgmental, coercive, or obstructive healthcare interactions |
| Although the doctor did not actually tell her she was “nothing,” she felt that his behavior was judgmental and dehumanizing. (U) |  |
| The doctor’s negative response to her age and the lack of compassionate care led her to conclude that she was, indeed, doing something morally wrong. (U) |  |
| Women’s experiences of obtaining an ultrasound were varied and depended on the individual provider’s assumptions about the pregnancy and scan. (U) |  |
| One woman decided to go directly to CSHS after a previous experience with a local GP. (U) |  |
| Even when accessing traditional medical care, they faced judgment. (U) |  |
| Religious judgment the CPC staff invoked. (U) |  |
| Judgmental and unhelpful interactions with clinicians. (U) |  |
| Began sharing “information” about the purported risks of abortion. (U) |  |
| One respondent described being stigmatized by a provider who, like this respondent, was also South Asian. (U) |  |
| She spoke at length to the stigmatizing conversation she deemed “irrelevant”. (U) |  |
| Creating barriers to abortion access. The main behaviors were providing insufficient information to abortion seekers, delaying or gatekeeping access to the service, or denying care altogether. (U) |  |
| Judging, blaming, questioning, or punishing abortion seekers. Participants described direct and Implicit communications from healthcare workers that made them feel blamed, judged, or punished for their pregnancy and subsequent abortion. (U) |  |
| Minimized interactions that compromise the quality and safety of abortion care. Many participants described receiving insufficient procedural or aftercare information, follow up, or time to support the recovery process. (U) |  |
| Further described how the legal origins of them being denied abortion care were central to the emotional costs of travel. (C) |  |
| Healthcare workers were rude, rushed, uncompassionate, and showed little concern for protecting patient privacy. (U) |  |
| Contextualizing misinformation through information of risk of pregnancy. (C) |  |
| Anti-abortion rhetoric encountered online included harmful stereotypes and threats of violence, with some participants expressing that this negatively impacted their abortion experience and self-perception more broadly. (U) | Online and public anti-abortion hostility |
| Stigma engendered by the law. (C) | Legal and structural barriers experienced as stigmatizing |
| The source of the negative emotions she and her husband experienced was state law. (U) |  |
| Gestational age limits influenced the experiences of many participants. Those nearing the 63-day gestational age limit for medication abortion had an urgent need to find timely appointments with a willing provider – a particular challenge among rural participants. (U) |  |
| Some participants lacked a choice in method due to limited provision in rural areas. (U) |  |

| **Synthesized finding 2 – Perceived abortion stigma**  People seeking abortion care perceived abortion as socially, morally, institutionally, and legally devalued, even when they did not necessarily report direct verbal attacks or explicit discrimination. This perception was shaped by disapproval in family and social networks, anti-abortion climates in communities and public spaces, stigmatizing healthcare and abortion-related services, restrictive legal contexts, and the gendered norms of motherhood and sexuality. Participants described their social networks as unsupportive or broadly disapproving of abortion, and some perceived their community and physical environments as saturated with anti-abortion messages. In healthcare contexts, perceived stigma emerged when participants felt criticized, unsupported, or not taken seriously by providers, when abortion was framed as an undesirable outcome, or when a lack of information, referral pathways, and emotional support made abortion care feel different from ordinary healthcare. Legal and structural conditions also contributed to perceived stigma: secrecy, criminalization, punitive laws, and limited access to information or services created an atmosphere of doubt and conveyed that abortion was morally questionable. Perceived stigma was further shaped by gendered expectations, including the idea that abortion represented a rejection of motherhood or violated cultural norms regarding sexuality and pregnancy outside marriage. Overall, perceived abortion stigma emerged as an awareness of a disapproving social, institutional, and legal climate in which abortion was treated as unsupported, morally contested, taboo, or deviant behaviour. | |
| --- | --- |
| **Findings** | **Categories** |
| Most women described abortion stigma – and their social network not being “supportive of [my decision]” (Destiny, 20, Black) – as a barrier to support and access to abortion services. (C) | Perceived disapproval in family and social networks |
| Experienced enacted stigma […] in their communities and physical environments as well. (U) | Perceived anti-abortion climate in communities and public spaces |
| Perceived their broader social networks to be disapproving of abortion. (U) |  |
| Consequences of stigma and the lack of respect or women to make reproductive decisions concerning their own bodies. (U) | Perceived stigma in healthcare and abortion-related services |
| Feeling judged outside the abortion clinic by anti-abortion protesters and she continued to feel uncomfortable after she entered the clinic. (U) |  |
| CPC counselors indirectly stigmatized abortion as a pregnancy outcome. (U) |  |
| Feeling criticised and unsupported by providers that were rude, dismissive or out-right judgmental. (U) |  |
| Participants went to crisis pregnancy centers (CPCs) for their ultrasound, often not realizing that CPCs are designed to convince patients to not obtain an abortion. (U) |  |
| The barriers of a lack of information, a lack of help from the healthcare community and the perceived stigma of abortion care are, of course, related. (U) |  |
| Necessity of going outside of her existing healthcare system to obtain an abortion inspired feelings of uncertainty about the morality of her abortion. (U) |  |
| Some respondents shared feeling stigmatized by healthcare professionals when seeking an abortion, for getting pregnant, choosing to abort, and/or having had prior abortions. (U) |  |
| Interactions that do not respond to abortion seekers’ emotional and information needs. Participants described interactions with healthcare workers that did not respond to their emotional and information needs at different points in the abortion pathway. (U) |  |
| Making assumptions about reproductive intentions and related preferences for care. (U) |  |
| Because of abortion stigma, this decision involved weighing risks. (U) |  |
| The secrecy surrounding abortion in general, coupled with the inaccessibility of clear information and resources about where to obtain one, created an atmosphere of doubt around Abortion. (U) | Legal and structural stigma |
| For several others, the criminalised nature of abortion made them feel defiant in the face of what they perceived to be un unfair law. (U) |  |
| The criminal status of abortion felt like a moral judgment and contributed to the feeling that abortion was some-thing that should not be talked about. (U) |  |
| Participants felt that the current laws were punitive rather than protective. (U) |  |
| Abortion represented a rejection of motherhood. (U) | Gendered norms of motherhood and sexuality |
| Many participants also explained that sex before marriage was unacceptable in their family and community and often equated the stigma of getting pregnant out of wedlock with the stigma of having an abortion. (U) |  |

| **Synthesized finding 3 – Internalized abortion stigma**  People seeking abortion care frequently internalize stigma, in the form of shame, and self-blame, particularly if they have undergone multiple abortions or belong to religious or marginalized communities. They may opt to avoid sharing their decision with others, leading to feelings of isolation and judgment. Misinformation and anti-abortion attitudes further exacerbate their negative emotions. The decision to undergo an abortion often depends on life-stage considerations and sound reasoning. However, stigma and mistrust of medical providers can pose difficulties in this process. | |
| --- | --- |
| **Findings** | **Categories** |
| Conflicted thoughts on having an abortion while acknowledging that proceeding was the best choice for them. (U) | Internalized shame, guilt, and self-blame |
| Silence was often described as being frustrating and challenging. (U) |  |
| Abortion was perceived as a rare and thus potentially deviant event. (U) |  |
| Internalised stigma was linked to feelings of shame and secrecy. (U) |  |
| Participants themselves were the most common source of disapproving attitudes toward abortion. (U) |  |
| Inconsistency between their antiabortion views and their behavior, several women described negative emotions. (U) |  |
| Spoke of emotions of loneliness and othering. (U) |  |
| Describing themselves as selfish. (U) |  |
| The fact that privacy was so important to many participants highlights the internalized and social stigma surrounding abortion that is not a consideration for most other medical procedures or healthcare services. (U) |  |
| Isolation and silence leading up to her own medication abortion continued to pervade after the abortion. (U) |  |
| Shame had to do with the stigma surrounding abortion, as the poem shows, wherein the abortion seeker felt that an unplanned pregnancy and subsequent abortion was something that only happened to stupid people. (U) |  |
| This resulted in internalized shame amongst participants surrounding their abortion decision. (U) |  |
| Women who had not been using contraception and became pregnant articulated blame as well as shame. (U) | Shame and self-blame related to Moral and religious conflict |
| Self-recrimination and shame for the unwanted pregnancy were internal motivations for requesting telemedicine abortion. (U) |  |
| Participants often felt internal stigma and worried that others would view them as irresponsible. (U) |  |
| Talk generally of not ‘believing’ in abortion, as an explanation for why they felt they had done something wrong. (C) |  |
| Religion Within the Experience of Internalized Abortion Stigma. (U) |  |
| Recognition of the increased potential for shame, even those who resisted self-blame. (U) |  |
| Women who had experienced more than one abortion there was a stronger sense of failure and self-blame. (U) | Shame and self-blame related to multiple abortions |
| Women who had experienced more than one unintended pregnancy and abortion, however, there was often a stronger voicing of self-blame, particularly for their sexual behaviour. (U) |  |
| Participants explained that they had amplified shame and stigma because of their need for more than one abortion. (U) |  |
| Would be punished later in her life for having two abortions. (U) |  |
| Valued female citizen has children at the right time and in the right way. (U) | Internalized norms of motherhood and femininity |
| Describing abortion as being tabooer than any other health condition. (U) | Internalized concerns about body, fertility, and reproductive health |
| Misperceptions about abortion’s jeopardizing their future fertility. (U) |  |
| Stigmatized anti-abortion narratives. (U) | Internalized effects of anti-abortion narratives |

| **Synthesized finding 4 – Anticipated abortion stigma**  Individuals seeking abortion anticipate stigmatization and criticism from their communities, families, and HPs. They are anxious about unfavorable reactions, social exclusion, and harm to their reputation. Many keep their abortions confidential and feel muted by their loved ones. Stigma is frequently interwoven with religion and law. Certain individuals prefer traveling for the procedure to avoid judgment. Lack of privacy and fear of protesters also serve as obstacles to accessing abortion services. | |
| --- | --- |
| **Findings** | **Categories** |
| Many women wished to keep their abortion secret because of either perceived or experienced stigma around abortion (U) | Anticipated judgement by healthcare providers and services |
| Expectation: Judgmental abortion provider. (U) | Anticipated harassment by protestors |
| Surprise that the experience was not as bad as she might have expected. (U) |  |
| Expectation: Pressure to make a particular decision. (U) |  |
| Expectation: Difficulty accessing abortion care. (U) |  |
| Expectation: Hostile clinic environment. (U) |  |
| Expectation: Traumatic abortion process. (U) |  |
| Potential for judgement was also apparent in her account of her interactions with health professionals. (C) |  |
| Fear of judgement through HPS at CPCs. (U) |  |
| Expectation: Protestors. (U) |  |
| Being surprised about the positive care that she received for her second termination, stating emphatically. (U) |  |
| Knowing there could be protestors was a barrier in accessing regular abortion care. (U) |  |
| Abortion stigma: Anticipated guilt and shame from local provider. (U) |  |
| Many participants described the additional benefits of avoiding perceived stigma and judgment from those at the facility, protesters, and people they needed to involve in accessing care, such as a family member who helped with childcare or a friend they asked for a ride. (U) |  |
| Experiencing stress and anxiety relating to the cultural and temporal factors. (U) | Fear of judgement by family, partners, and social networks |
| Many women had reduced social support because they were unable to tell individuals in their network, or their entire network, about their abortion due to concerns of stigmatization. (U) |  |
| Negative views in partners with whom they did not discuss the abortion. (U) |  |
| Many participants, abortion stigma influenced if, when, and to whom they felt comfortable disclosing their pregnancy status. (C) |  |
| Women noted feelings of shame, embarrassment, worry, or fear as some of the reasons for not telling others. (U) |  |
| Others described the guilt and shame they would feel disclosing their abortion to their regular provider, such as a 28- year-old Black woman, who did not return for follow-up care because she no longer had pregnancy symptoms. (U) |  |
| Some women perceived that people writ large would judge them negatively about their choice to have an abortion. (U |  |
| One such participant felt that she would have disappointed her family if they knew she was having an abortion. (U) |  |
| Concerns about being judged, women also expressed a desire to maintain privacy. (U) |  |
| Difficult to talk with family and friends because they were often afraid of their emotional or judgemental reactions. (U) |  |
| Women’s desires to talk about their abortion, others (e.g., the baby’s father, their husband, family members) refused to engage in conversation with them. (U) |  |
| Felt muted by their loved ones because of the centripetal discourses of shame and embarrassment associated with abortion. (U) |  |
| Anticipated stigmatizing reactions from others, which led to self-protective secretive behavior. (U)  Not telling someone in their social circle about their abortion specifically to avoid arguments, social isolation or damage to their reputation. (U)  Other reasons participants chose not to tell their family about their abortion included: fear of judgement, stigma related to having sex outside of marriage and getting pregnant, as well as stigma surrounding abortion. (U)  For these women, nondisclosure was a tool to manage stigma and prevent what they perceived to be irreparable damage to the most important relationships in their lives. (U) |  |
| Living in smaller communities often posed the risk of seeing familiar people, which compounded fears of judgment. (U) | Fear of community exposure and loss of privacy |
| Concerned about the opinions of others in her community. (U) |  |
| Fear of judgment extended to the larger community. (U) |  |
| Expectation: Community stigma. (U) |  |
| For migrants, […] keeping the abortion secret was often imperative and was motivation for seeking a self-managed abortion. (U) |  |
| Concerns about stigma and the impact on their career should anyone in the military find out about their pregnancy or abortion. (U) | Anticipated stigma in institutional or career contexts |
| She found out that she was pregnant for a second time and made the decision to have an abortion, Miranda travelled to another city for her procedure because she was concerned about being judged. (U) | Anticipated stigma related to repeat abortion |
| Avoided discussing her difficult decision with her counselor due to concerns about her counselor’s religious convictions. (U) | Anticipated religious condemnation |
| Felt stigma was entwined with religion for our respondents; women specifically feared religiously based condemnation if they revealed that they had had abortions. (U) |  |

| **Synthesized finding 5 – Mitigating factors of abortion stigma**  People seeking abortion care reported several beneficial factors, including positive interactions with medical personnel, medication abortions to ensure privacy, supportive environments with fellow women, and the use of telemedicine to sidestep judgement and stigma. Moreover, participants discussed their strategies for coping with religious-based stigma and provided rationales for their decision to undergo an abortion, such as the well-being of their existing children or their financial stability. Non-judgmental behavior from healthcare providers and support from family and friends were significant factors. Although there was a possibility of harassment, sharing abortion stories publicly was viewed as a positive experience. | |
| --- | --- |
| **Findings** | **Categories** |
| Participants without children also drew upon notions about the ‘right time’, to become a mother. (U) | Support, openness, and shared abortion experiences |
| Free of the stigma associated with abortion, she received the sympathy and support of her friends and partner that she desired. (U) |  |
| Openness about abortion is positioned as entirely possible within the context of close friendships, but it is not something that can reasonably be mentioned in casual interaction. (U) |  |
| People’s reactions to hearing about a woman’s decision to have an abortion ranged from positive/supportive. (U) |  |
| Women described receiving some form of social support during their abortion. (U) |  |
| When they shared their experience it became evident how many other women had not only had an abortion but had kept it secret. (U) |  |
| No trouble discussing her decision-making with her family and friends. (U) |  |
| Explain that she did not feel abortion should be silenced, and that she was happy to discuss her experience. (U) |  |
| Not feeling embarrassment. (U) |  |
| Clients relied on their social networks to offset the stigmatization that occurred at a CPC. (U) |  |
| They described the supportive environment created when women could all be together in one room, before and after the abortion. (U) |  |
| Respondents described the benefits of these positive experiences, such as the gratification of helping others who had had abortion. (U) | Public storytelling and stigma resistance |
| Reported the satisfaction of having an impact on policymakers. (U) |  |
| Without this knowledge, viewing online accounts was presented as a way to reduce feelings of loneliness without feeling obliged to make public that they had undergone, or were about to undergo an abortion, thereby avoiding potential stigmatising interaction. (U) |  |
| Reported that sharing their stories publicly lessened their own feelings of shame or guilt. (U) |  |
| Harassment actually encouraged them to continue sharing, as it demonstrated that harmful antiabortion attitudes are still prevalent. (U) |  |
| Women’s positioning of themselves as ‘‘open’’ has to be carefully navigated in relation to admissions of non-disclosure in which they risk positioning themselves as ‘‘ashamed’’ or ‘‘embarrassed.’’. (U) | Non-judgmental and supportive healthcare |
| Helpful interaction with a medical assistant prior to the abortion. (U) |  |
| Reported a non-judgemental approach by both clinical and non-clinical staff at CSHS. (U) |  |
| The appointment for the MToP procedure was overall a positive, personal experience. (U) |  |
| Participants emphasized the importance of a non-judgmental approach. (U) |  |
| Emphasized the importance of using kind language and compassion to address the conversation in a sensitive manner. (U) |  |
| Most women were pleased with how they were treated by HPs in the abortion clinic. (U) |  |
| Actual experience: Nonjudgmental abortion provider with TelAbortion. (U) |  |
| Found that telemedicine offered support and information while simultaneously protecting her from judgement and social stigma. (U) |  |
| Centered the participant by making the process easy, prioritizing their needs, and treating them with respect (TelAbortion). (U) |  |
| Her past abortion experience was unacceptable to her due to the loss of privacy she experienced while in the clinic and having a direct-to-patient telemedicine abortion provided an appealing alternative. (U) |  |
| Insisted that the CPC was not a place that considered abortion a bad thing. (U) |  |
| Contributed to her perceiving the CPC itself as not antiabortion. (U) |  |
| Rather than express judgment or shock, the counselor seemed to take it in stride. (U) |  |
| Participants expressed gratitude for empathy, non-judgemental and attention to the unique circumstances they identified in interactions with providers. (U) |  |
| How the clinic, in contrast to the CPC, did not induce guilt through enacted stigma. (U) |  |
| Actively helping people access abortion care. Some providers, who did not themselves provide abortion care because of health systems constraints, proactively provided abortion information, or helped organise the referral and subsequent appointments. (U) |  |
| Interactions responsive to evolving emotional and information needs. Many participants described interactions in which healthcare workers offered comfort and emotional support during and after the abortion service, supported an informed decision-making process, or ensured the ser-vice aligned with their needs and preferences. (U) |  |
| Aligning abortion provision with client reproductive intentions and service preferences. Participants described positive interactions in which the provider sought to understand their reproductive intentions and service-related preferences, and then aimed to provide care accordingly. (U) |  |
| Providing holistic and high-quality care to ensure safety. Some participants described receiving sufficient aftercare information, follow up, and supportive care during recovery, which prepared them to manage their abortion safely. (U) |  |
| Unintended pregnancies that had occurred despite what was described as careful and responsible contraceptive use. (U) |  |
| Religious identity as giving her to tools to resist stigmatization by CPC staff. (U) |  |
| Credited clinic escorts and security with preventing more involved encounters with protestors. (U) | Validation of the abortion decision |
| Clinic escorts also played music and covered the fence around the clinic perimeter with a dark tarp to further shield patients from hearing and seeing protestors […] participants found these strategies to be helpful. (U) |  |
| Sofia was grateful that her decision to end the pregnancy was validated and understood as moral by her providers. (U) |  |
| Actual experience: Not feeling pressured to make a particular decision with TelAbortion. (U) | Empowerment, justification, and positive reframing |
| Actively validating abortion decision. Participants also experienced interactions that actively validated and supported their decision to have an abortion with different types of healthcare workers along the care pathway. (U) |  |
| Distinguish the circumstances of their abortions from those of other women’s abortions. (U) |  |
| Valid reason they pursued one. (U) |  |
| Deciding to have an abortion in the interests of existing children, thereby drawing on gendered notions of ‘good mothering’ was one of the most strongly expressed justifications for an abortion. (U) |  |
| Expresses strong moral opposition to abortion she is able to justify her own abortion by pointing to her extremely poor health. (U) |  |
| Concern was not having to struggle financially. (U) |  |
| Driver in decision-making was rejection of unplanned motherhood in the context of what they thought might not be a lasting relationship. (U) |  |
| Women position themselves as making a personal choice/decision, which does not impact on the agency of other people. (U) | Religious reinterpretation and resistance to stigma |
| Narrative thereby contains elements of a stigma narrative she has indirectly rejected internalised abortion stigma. (U) |  |
| Neither the health “information” nor the religious claims changed Sierra’s mind about abortion as the right choice for her. (U) |  |
| Strategies for Managing Religiously Based Abortion Stigma: Personal Exceptionalism. (U) |  |
| Strategies for Managing Religiously Based Abortion Stigma: Rejecting Religion. (U) |  |
| Strategies for Managing Religiously Based Abortion Stigma: Revising Beliefs. (U) |  |
| Strategies for Managing Religiously Based Abortion Stigma: Challenging a Stigmatized Status. (U) |  |
| Rather than tap into an existing internalized stigmatization of abortion, these religious references had no effect. (U) |  |
| Pointing to the fact that they had had previous abortions and had not been punished by God. (U) | Telemedicine and medication abortion as stigma-reducing options |
| The only true judge of their behavior was God. (U) |  |
| Several participants commented that physical distance from protestors would have provided them with the privacy they desired. (U) |  |
| Actual experience: No protestors with TelAbortion. (U) |  |
| To decrease the possibility of feeling exposed, some participants […] had a medication abortion instead of a procedure. (U) |  |
| Apprehension at “the thought of having to go to a clinic and speak to a nurse.” (C) |  |
| Actual experience: No hostile clinic environment with TelAbortion. (U) |  |
| Actual experience: Non-Traumatic abortion process with TelAbortion. (U) |  |
| Actual experience: Not experiencing community stigma with TelAbortion. (U) |  |
| Actual experience: Not experiencing difficulty accessing abortion care with TelAbortion. (U) |  |
